# Clinical outcomes in patients with atrial fibrillation and a history of falls using non-vitamin K antagonist oral anticoagulants: A nationwide cohort study

**DOI:** 10.1016/j.ijcha.2023.101223

**Published:** 2023-05-18

**Authors:** Maxim Grymonprez, Mirko Petrovic, Tine L. De Backer, Stephane Steurbaut, Lies Lahousse

**Affiliations:** a Department of Bioanalysis, Pharmaceutical Care Unit, Faculty of Pharmaceutical Sciences, Ghent University, Ottergemsesteenweg 460, 9000 Ghent, Belgium; b Department of Geriatrics, Ghent University Hospital, C. Heymanslaan 10, 9000 Ghent, Belgium; c Department of Cardiology, Ghent University Hospital, C. Heymanslaan 10, 9000 Ghent, Belgium; d Centre for Pharmaceutical Research, Research group of Clinical Pharmacology and Clinical Pharmacy, Vrije Universiteit Brussel, Laarbeeklaan 103, 1090 Jette, Belgium; e Department of Hospital Pharmacy, UZ Brussel, Laarbeeklaan 101, 1090 Jette, Belgium; f Department of Epidemiology, Erasmus Medical Center, PO Box 2040, Rotterdam 3000, CA, the Netherlands

**Keywords:** Atrial fibrillation, Fall, Anticoagulation, Thromboembolism, Bleeding, Mortality

## Abstract

**Background:**

Data on non-vitamin K antagonist oral anticoagulant (NOAC) use in patients with atrial fibrillation (AF) and a history of falls are limited. Therefore, we investigated the impact of a history of falls on AF-related outcomes, and the benefit-risk profiles of NOACs in patients with a history of falls.

**Methods:**

Using Belgian nationwide data, AF patients initiating anticoagulation between 2013 and 2019 were included. Previous falls that occurred ≤ 1 year before anticoagulant initiation were identified.

**Results:**

Among 254,478 AF patients, 18,947 (7.4%) subjects had a history of falls, which was associated with higher risks of all-cause mortality (adjusted hazard ratio (aHR) 1.11, 95%CI (1.06–1.15)), major bleeding (aHR 1.07, 95%CI (1.01–1.14)), intracranial bleeding (aHR 1.30, 95%CI (1.16–1.47)) and new falls (aHR 1.63, 95%CI (1.55–1.71)), but not with thromboembolism. Among subjects with a history of falls, NOACs were associated with lower risks of stroke or systemic embolism (aHR 0.70, 95%CI (0.57–0.87)), ischemic stroke (aHR 0.59, 95%CI (0.45–0.77)) and all-cause mortality (aHR 0.83, 95%CI (0.75–0.92)) compared to vitamin K antagonists (VKAs), while major, intracranial, and gastrointestinal bleeding risks were not significantly different. Major bleeding risks were significantly lower with apixaban (aHR 0.77, 95%CI (0.63–0.94)), but similar with other NOACs compared to VKAs. Apixaban was associated with lower major bleeding risks compared to dabigatran (aHR 0.78, 95%CI (0.62–0.98)), rivaroxaban (aHR 0.78, 95%CI (0.68–0.91)) and edoxaban (aHR 0.74, 95%CI (0.59–0.92)), but mortality risks were higher compared to dabigatran and edoxaban.

**Conclusions:**

A history of falls was an independent predictor of bleeding and death. NOACs had better benefit-risk profiles than VKAs in patients with a history of falls, especially apixaban.

## 1 Introduction

The prevalence of atrial fibrillation (AF) increases with age and is an independent risk factor for falls [1], [2], [3]. Physicians tend to be reluctant to initiate anticoagulation therapy in AF patients with a history of falls due to the perception that fall-related intracranial bleeding risks outweigh benefits of anticoagulation [4]. Consequently, a history of falls has been associated with non-initiation [4], [5], delayed initiation [6], discontinuation [7], [8] and inappropriate underdosing [9] of oral anticoagulants (OACs) in patients with AF. Although a history of falls should not generally be viewed as a reason to withhold anticoagulation according to guidelines [10], [11], higher risks of death and major bleeding have been observed among AF patients with versus without a history of falls [8], [12], [13], [14], [15]. The evidence on whether or not a history of falls in AF patients may impact thromboembolic and intracranial bleeding risks is however scarce and more conflicting. Previous studies were often limited by small sample sizes, inadequate inclusion criteria (e.g., inclusion of non-anticoagulated or OAC-experienced patients), or did not provide data regarding the use of non-vitamin K antagonist oral anticoagulants (NOACs) [13], [14], [15], [16].

NOACs are now the preferred and most frequently used option for stroke prevention in the general population with AF [10], [17]. However, whether or not the benefit-risk profile of NOACs is maintained in real-life patients with a history of falls is largely unknown [11]. To the best of our knowledge, only two post-hoc analyses of randomized controlled trials (RCTs) [8], [12] and one observational cohort study [18] have investigated outcomes with rivaroxaban, apixaban and/or edoxaban compared to vitamin K antagonists (VKAs) in AF patients with a history of falls. However, small sample sizes (e.g., 753 and 900 AF patients at risk of falling were investigated in post-hoc analyses of the ARISTOTLE and ENGAGE AF-TIMI 48 trial, respectively) [8], [12], short follow-up durations and lack of data on dabigatran limited the generalization of results. Moreover, no head-to-head comparisons between NOACs have yet been performed in AF patients with a history of falls. Consequently, there is an urgent need for a critical appraisal of the benefit-risk profile of all marketed NOACs in patients with a history of falls to guide physicians in clinical practice.

Therefore, in the present study, we aimed to investigate whether patients with AF and a history of falls had worse clinical outcomes than patients without a history of falls. Moreover, whether or not the benefit-risk profile of individual NOACs was preserved compared to VKAs in patients with AF and a history of falls, was examined.

## 2 Methods

### 2.1 Source population

Details on the study methodology have been reported before [7], [19], [20]. In brief, two nationwide databases, the InterMutualistic Agency (IMA) database and Minimal Hospital Dataset (MHD), provided the source population. The IMA centralizes the claims data from Belgian health insurance funds on reimbursed ambulatory and hospital care, including demographic characteristics, medical procedures, and drug prescription claims, and represents all legal residents in Belgium [21]. The MHD aggregates hospital discharge diagnoses of every hospital admission (hospitalizations, day-care stays, and emergency room contacts), coded in International Classification of Diseases (ICD) codes (ICD-9 up to 2014, ICD-10 from 2015 onwards) [22]. Every case of the study population was identified in both databases. This study was approved by the Belgian Commission for the Protection of Privacy (approval code IVC/KSZG/20/344), waiving the need for individual informed consents [23]. The Strengthening the Reporting of Observational Studies in Epidemiology (STROBE) reporting guideline was followed (eTable 1) [24].

### 2.2 Study population

Patients ≥ 45 years old with ≥ 1 year coverage by health insurance funds were included from the IMA database on the first date of filling an OAC prescription (=index date) from January 1st, 2013, to January 1st, 2019 (eFigure 1). NOAC users, namely dabigatran (approved in Belgium since August 2012), rivaroxaban (approved since September 2012), apixaban (approved since September 2013) and edoxaban (approved since October 2016), and VKA users (warfarin, acenocoumarol, phenprocoumon) were included [19]. Only OAC-naïve subjects were considered, excluding subjects with an OAC prescription filled ≤ 1 year before the index date. Subjects were not required to have an ICD-coded hospital discharge diagnosis of AF to be included, as this would create selection bias due to limiting the study population to hospitalized AF subjects and excluding AF subjects treated exclusively in primary or ambulatory care [20], [25].

Persons were excluded in case of (1) total hip or knee replacement, or diagnosis of deep vein thrombosis or pulmonary embolism ≤ 6 months before the index date, (2) mechanical prosthetic heart valve or moderate/severe mitral stenosis, (3) end-stage renal disease (chronic kidney disease stage V and/or dialysis), (4) ≥ 2 prescription claims of different OAC types or doses on the index date, or (5) use of NOAC doses not approved for stroke prevention in AF (e.g., rivaroxaban 10 mg) (eTable 2, eFigure 1).

### 2.3 History of falls

In line with previous research [8], [13], [16], a history of falls in patients initiating anticoagulation was defined as at least one fall ≤ 1 year before the index date, identified using ICD-coded hospital discharge diagnoses such as ICD-10 codes Z91.81 (‘history of falls’), R29.6 (‘repeated falls’) and W19 (‘unspecified fall’) (eTable 2).

### 2.4 Outcomes

Primary outcomes included stroke or systemic embolism (stroke/SE), ischemic stroke, all-cause mortality, major bleeding, intracranial bleeding, and gastrointestinal bleeding. As a secondary outcome, new falls during follow-up were assessed. Stroke/SE was defined as a composite of ischemic, hemorrhagic or unspecified stroke, and non-cerebral systemic embolism (eTable 3). Major bleeding was defined as a hospitalized bleeding event in a critical area or organ (e.g., intracranial), fatal bleeding or bleeding event with a medical procedure code for blood transfusion ≤ 10 days after admission, which is adapted from the International Society on Thrombosis and Haemostasis definition due to a lack of data on haemoglobin levels or number of blood transfusion units [20], [26], [27]. Outcomes were identified using ICD-coded hospital discharge diagnoses and medical procedure codes (eTable 3) [7]. The incident date of outcomes was defined as the date of hospital admission or date of registration for medical procedures, whichever occurred first [20].

### 2.5 Follow-up

Subjects were followed from OAC initiation until the first occurrence of the investigated outcome, discontinuation (>60-day gap of drug supply) or switch of treatment, death, emigration or end of the study period (January 1st, 2019), whichever came first (on-treatment analysis) [7].

### 2.6 Covariates

Baseline characteristics were assessed on the index date and included age, sex, comorbidities, medication history and clinical risk scores. Comorbidities were identified with specific ICD-coded diagnoses, medical procedure codes and/or medication prescription claims ≤ 1 year before the index date (eTable 2). Medication history was identified with medication prescription claims, considering recent use ≤ 6 months before the index date. The CHA_2_DS_2_-VASc score, modified HAS-BLED score (without the ‘labile INR’ criterion) and age-adjusted Charlson comorbidity index were calculated [10], [28].

### 2.7 Statistical analyses

Mean and standard deviation (SD) and counts and percentages were presented for continuous and categorical variables, respectively. Crude event rates were calculated as the total number of events per 100 person-years at risk. Primary and secondary outcomes were compared in AF patients initiating anticoagulation with and without a history of falls using Cox proportional hazard regression models. Additionally, models were adjusted for age and sex (age- and sex-adjusted model); and for age, sex, type of OAC used, baseline comorbidities and medication history (multivariable adjusted model with covariates described in Table 1). Only statistically significant factors using a two-sided p-value of < 0.05 were retained in the multivariable adjusted model with backward elimination.

**Table 1:** Baseline characteristics.

**Patient characteristics**		**No history of falls** **(n = 235,531)**	**History of falls**			SMD*	
			**Overall history of falls (n = 18,947)**	**VKA** **(n = 3676)**	**NOAC** **(n = 15,271)**	**Before IPTW**	**After IPTW**
	Age (years)	74.5 ± 10.8	80.9 ± 9.6	78.3 ± 11.3	81.5 ± 9.0	0.317	0.057
	Female	109,597 (46.5%)	11,177 (59.0%)	2042 (55.5%)	9135 (59.8%)	0.087	0.041
	Follow-up (years)	1.3 ± 1.5	0.9 ± 1.1	0.7 ± 1.1	0.9 ± 1.1	NA	NA
**Comorbidities**							
	Hypertension	149,520 (63.5%)	15,356 (81.0%)	2993 (81.4%)	12,363 (81.0%)	0.001	0.013
	Coronary artery disease	42,023 (17.8%)	5820 (30.7%)	1440 (39.2%)	4381 (28.7%)	0.201	0.012
	Congestive heart failure	33,720 (14.3%)	6173 (32.6%)	1262 (34.3%)	4911 (32.2%)	0.021	0.013
	Valvular heart disease	30,599 (13.0%)	5563 (29.4%)	1303 (35.4%)	4260 (27.9%)	0.139	0.033
	Peripheral artery disease	17,714 (7.5%)	3222 (17.0%)	822 (22.4%)	2400 (15.7%)	0.125	0.006
	Dyslipidemia	132,975 (56.5%)	10,940 (57.7%)	2242 (61.0%)	8698 (57.0%)	0.082	0.002
	Chronic kidney disease	23,445 (10.0%)	6050 (31.9%)	1434 (39.0%)	4616 (30.2%)	0.155	0.009
	Chronic liver disease	6570 (2.8%)	1887 (10.0%)	457 (12.4%)	1430 (9.4%)	0.052	0.015
	Chronic lung disease	27,087 (11.5%)	4958 (26.2%)	1137 (30.9%)	3821 (25.0%)	0.091	0.016
	Obstructive sleep apnea	7955 (3.4%)	818 (4.3%)	211 (5.7%)	607 (4.0%)	0.050	0.028
	Cancer	21,733 (9.2%)	3455 (18.2%)	667 (18.1%)	2788 (18.3%)	0.036	0.035
	Upper GI tract disorder^**^	15,410 (6.5%)	3768 (19.9%)	852 (23.2%)	2916 (19.1%)	0.065	0.019
	Lower GI tract disorder^**^	15,247 (6.5%)	2410 (12.7%)	527 (14.3%)	1883 (12.3%)	0.011	0.002
	Diabetes mellitus	68,182 (28.9%)	14,521 (76.6%)	3113 (84.7%)	11,408 (74.7%)	0.229	0.067
	Anemia	15,393 (6.5%)	5720 (30.2%)	1388 (37.8%)	4331 (28.4%)	0.164	0.039
	Thyroid disease	32,686 (13.9%)	4222 (22.3%)	901 (24.5%)	3321 (21.7%)	0.035	0.030
	Depression	49,609 (21.1%)	7627 (40.3%)	1523 (41.4%)	6104 (40.0%)	0.030	0.056
	Dementia	9323 (4.0%)	4238 (22.4%)	865 (23.5%)	3373 (22.1%)	0.003	0.052
	Parkinson’s disease	6166 (2.6%)	1390 (7.3%)	266 (7.2%)	1124 (7.4%)	0.005	0.015
	Frailty	58,879 (25.0%)	13,605 (71.8%)	2422 (65.9%)	11,183 (73.2%)	0.191	0.058
	Prior stroke/SE	29,566 (12.6%)	5829 (30.8%)	1253 (34.1%)	4576 (30.0%)	0.042	0.031
	Prior MB/CRNMB	10,759 (4.6%)	3520 (18.6%)	815 (22.2%)	2704 (17.7%)	0.060	0.017
**Medication history**							
	Number of concomitant drugs	6.4 ± 4.0	9.7 ± 5.3	10.1 ± 5.5	9.6 ± 5.2	0.084	0.045
	Beta blockers	139,070 (59.0%)	12,747 (67.3%)	2266 (61.6%)	10,481 (68.6%)	0.147	0.025
	Verapamil, diltiazem	9142 (3.9%)	761 (4.0%)	145 (3.9%)	616 (4.0%)	0.005	0.002
	Digoxin	19,665 (8.3%)	2866 (15.1%)	389 (10.6%)	2477 (16.2%)	0.166	0.019
	Class I AAD	22,195 (9.4%)	1106 (5.8%)	153 (4.2%)	953 (6.2%)	0.094	0.017
	Class III AAD	56,106 (23.8%)	5345 (28.2%)	966 (26.3%)	4379 (28.7%)	0.054	0.014
	Acetylsalicylic acid	92,229 (39.2%)	7752 (40.9%)	1493 (40.6%)	6259 (41.0%)	0.008	0.011
	P2Y12 inhibitor	13,302 (5.6%)	1379 (7.3%)	273 (7.4%)	1106 (7.2%)	0.007	0.026
	Proton pump inhibitor	91,448 (38.8%)	10,800 (57.0%)	2179 (59.3%)	8621 (56.5%)	0.057	0.035
	NSAID	58,503 (24.8%)	4479 (23.6%)	921 (25.1%)	3558 (23.3%)	0.041	0.011
	Oral corticosteroids	46,571 (19.8%)	5568 (29.4%)	1167 (31.7%)	4401 (28.8%)	0.064	0.012
	SSRI/SNRI	27,293 (11.6%)	4034 (21.3%)	830 (22.6%)	3204 (21.0%)	0.039	0.054
**Clinical risk score**							
	CHA_2_DS_2_-VASc score	3.3 ± 1.8	5.2 ± 1.7	5.2 ± 1.8	5.2 ± 1.6	0.002	0.070
	HAS-BLED score	2.4 ± 1.2	3.6 ± 1.4	3.7 ± 1.5	3.5 ± 1.4	0.080	0.007
	Charlson Comorbidity Index	4.1 ± 2.1	6.7 ± 2.8	6.9 ± 3.2	6.7 ± 2.7	0.034	0.027

Table 1: Baseline characteristics of OAC-naïve AF patients with and without a history of falls at baseline.

Data shown as mean ± standard deviation or counts and percentages. NOAC users without a history of falls (35.8% reduced dose) included 26,439 dabigatran, 69,525 rivaroxaban, 60,389 apixaban and 21,448 edoxaban users; NOAC users with a history of falls (53.2% reduced dose) included 1705 dabigatran, 4896 rivaroxaban, 6536 apixaban and 2134 edoxaban users. VKA users without a history of falls included 27,672 acenocoumarol, 15,839 warfarin and 14,219 phenprocoumon users; VKA users with a history of falls included 1978 acenocoumarol, 1020 warfarin and 678 phenprocoumon users.

*Absolute SMDs illustrated for comparison of NOACs versus VKAs in patients with a history of falls before and after stabilized inverse probability of treatment weighting (IPTW). ^**^Upper and lower gastrointestinal tract disorders were defined as gastroesophageal reflux disease or peptic ulcer disease; and diverticulosis, angiodysplasia, colorectal polyposis or hemorrhoids, respectively. AAD: antiarrhythmic drug; AF: atrial fibrillation; CRNMB: clinically relevant non-major bleeding; GI: gastrointestinal; MB: major bleeding; NA: not applicable; NOAC: non-vitamin K antagonist oral anticoagulant; NSAID: non-steroidal anti-inflammatory drug; OAC: oral anticoagulant; SE: systemic embolism; SMD: standardized mean difference; SNRI: serotonin and norepinephrine reuptake inhibitor; SSRI: selective serotonin reuptake inhibitor; VKA: vitamin K antagonist.

Moreover, primary outcomes were compared between NOACs and VKAs, and between individual NOACs in patients with AF and a history of falls using stabilized inverse probability of treatment weighting (IPTW). In comparisons with apixaban and edoxaban, the study population was restricted to subjects having initiated treatment from September 2013 onwards and from October 2016 onwards respectively, to avoid violations of the positivity assumption [18], [29]. Propensity scores (PS) were calculated with logistic regression models including the 39 confounding covariates described in Table 1 (demographics, comorbidities, medication history, risk scores), stratified by calendar year. Based on the PS, stabilized weights were calculated and truncated at the 0.5th and 99.5th percentile. Covariate balance before and after weighting was checked using standardized mean differences with a ≥ 0.1 threshold to indicate imbalance. Weighted Cox proportional hazard regression models were used to calculate adjusted hazard ratios (aHRs) with 95% confidence intervals (CIs). The proportional hazard assumption was assessed using scaled Schoenfeld residuals. A two-sided p-value of < 0.05 was considered statistically significant. All analyses were performed in R (R version 3.6.0).

### 2.8 Sensitivity analyses

Sensitivity analyses were performed to check the robustness of results on the benefit-risk profile of OACs in AF patients with a history of falls. To examine whether estimates were affected by differential censoring between treatment groups, analyses were repeated using an intention-to-treat approach defining the end of follow-up as the first occurrence of an outcome, death, emigration, or end of study period, whichever occurred first. To verify the AF population and reduce misclassification bias, only subjects with an ICD-coded hospital discharge diagnosis of AF before or up to 90 days after the index date were investigated [25]. To evaluate outcomes when all NOACs were commercially available in Belgium, the study population was restricted to subjects having initiated treatment between October 1st, 2016 and January 1st, 2019.

## 3 Results

### 3.1 Baseline characteristics

A total of 254,478 AF patients initiating anticoagulation were included, among whom 18,947 (7.4%) subjects had a history of falls in the previous year (eFigure 2). AF patients with a history of falls were older (80.9 (SD 9.6) versus 74.5 (SD 10.8) years) and more frequently female (59.0% versus 46.5%), had higher CHA_2_DS_2_-VASc (5.2 (SD 1.7) versus 3.3 (SD 1.8)) and HAS-BLED scores (3.6 (SD 1.4) versus 2.4 (SD 1.2)), had more cardiovascular comorbidities and used more drugs concomitantly (9.7 (SD 5.3) versus 6.4 (SD 4.0)) than patients without a history of falls (Table 1). Baseline characteristics of the 15,271 NOAC users (1705 dabigatran, 4896 rivaroxaban, 6536 apixaban and 2134 edoxaban users) and 3676 VKA users with a history of falls before weighting are summarized in Table 1 and eTable 4. After weighting, covariate balance was achieved (Table 1, eFigure 3).

### 3.2 History of falls versus no previous falls

AF patients with and without a history of falls had a mean follow-up of 0.9 (SD 1.1) years (16,587 person-years) and 1.3 (SD 1.5) years (312,209 person-years), respectively. The unadjusted number of events and event rates are summarized in eTable 5. Crude, age- and sex-adjusted, and multivariable aHRs of outcomes are summarized in Table 2. After multivariable adjustment, a history of falls was associated with significantly higher risks of all-cause mortality (aHR 1.11, 95%CI (1.06–1.15), p-value < 0.001), major bleeding (aHR 1.07, 95%CI (1.01–1.14), p-value < 0.001), intracranial bleeding (aHR 1.30, 95%CI (1.16–1.47), p-value < 0.001) and new falls (aHR 1.63, 95%CI (1.55–1.71), p-value < 0.001), whereas the risks of stroke/SE (aHR 1.06, 95%CI (0.97–1.16), p-value 0.173), ischemic stroke (aHR 1.07, 95%CI (0.96–1.20), p-value 0.238) and gastrointestinal bleeding (aHR 1.02, 95%CI (0.93–1.11), p-value 0.704) were not significantly different.

**Table 2:** History of falls versus no previous falls.

	**History of falls versus no previous falls**		
	**Crude HR (95%CI)**	Age- and sex-adjusted HR (95%CI)*	Multivariable adjusted HR (95%CI)^**^
**Primary outcomes**			
Stroke/SE	1.78 (1.64–1.93)	1.60 (1.48–1.73)	1.06 (0.97–1.16)
Ischemic stroke	1.95 (1.76–2.17)	1.63 (1.47–1.82)	1.07 (0.96–1.20)
All-cause mortality	3.01 (2.90–3.12)	2.06 (1.99–2.14)	1.11 (1.06–1.15)
Major bleeding	1.75 (1.65–1.85)	1.59 (1.50–1.68)	1.07 (1.01–1.14)
Intracranial bleeding	1.65 (1.47–1.85)	1.65 (1.47–1.85)	1.30 (1.16–1.47)
Gastrointestinal bleeding	1.79 (1.65–1.94)	1.55 (1.43–1.68)	1.02 (0.93–1.11)
**Secondary outcomes**			
New fall	3.18 (3.04–3.32)	2.35 (2.25–2.46)	1.63 (1.55–1.71)

Table 2: Crude, age- and sex-adjusted, and multivariable adjusted hazard ratios with 95% confidence intervals of outcomes compared between anticoagulated AF patients with versus without a history of falls using Cox proportional hazard regression models.

* Adjusted for age and sex. ^**^ Adjusted for age, sex, OAC type, baseline comorbidities and medication history with backward elimination. AF: atrial fibrillation; CI: confidence interval; HR: hazard ratio; OAC: oral anticoagulant; SE: systemic embolism.

### 3.3 NOACs versus VKAs in patients with a history of falls

**Fig. 1:**
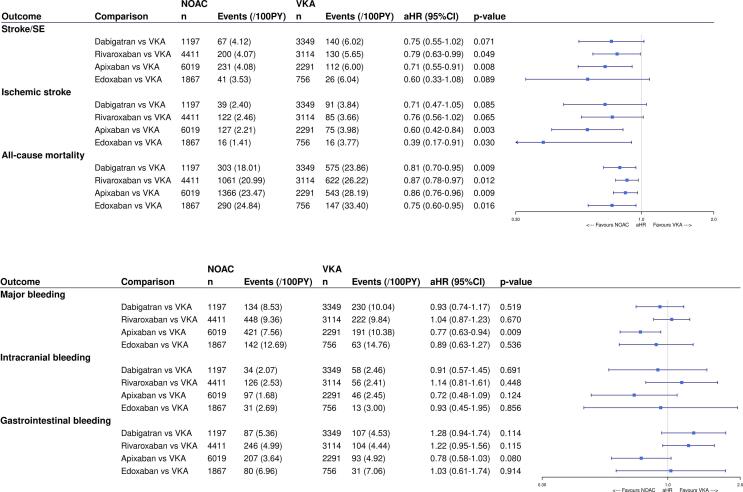
The **A)** effectiveness and **B)** safety of NOACs versus VKAs in AF patients with a history of falls after IPTW. The weighted number of subjects at risk in the pseudopopulation, weighted number of events, weighted event rates per 100 PY and aHRs with 95%CIs after IPTW are illustrated. aHR: adjusted hazard ratio; CI: confidence interval; IPTW: inverse probability of treatment weighting; NOAC: non-vitamin K antagonist oral anticoagulant; PY: person-years; Ref: reference category; SE: systemic embolism; VKA: vitamin K antagonist; vs: versus.

Among AF patients with a history of falls, NOACs were associated with significantly lower risks of stroke/SE (aHR 0.70, 95%CI (0.57–0.87), p-value < 0.001), ischemic stroke (aHR 0.59, 95%CI (0.45–0.77), p-value < 0.001) and all-cause mortality (aHR 0.83, 95%CI (0.75–0.92), p-value < 0.001) compared to VKAs after multivariable adjustment (eTable 6, Fig. 1). However, the risks of major bleeding (aHR 0.89, 95%CI (0.76–1.05), p-value 0.158), intracranial bleeding (aHR 0.86, 95%CI (0.62–1.19), p-value 0.376) and gastrointestinal bleeding (aHR 1.03, 95%CI (0.81–1.30), p-value 0.809) were not significantly different between NOACs and VKAs in this patient subgroup. Consistent trends were demonstrated with dabigatran, rivaroxaban, apixaban and edoxaban compared to VKAs. However, the risk of major bleeding was significantly lower with apixaban (aHR 0.77, 95%CI (0.63–0.94), p-value 0.009) compared to VKAs, while not significantly different with other NOACs.

### 3.4 Comparisons between NOACs in patients with a history of falls

**Fig. 2:**
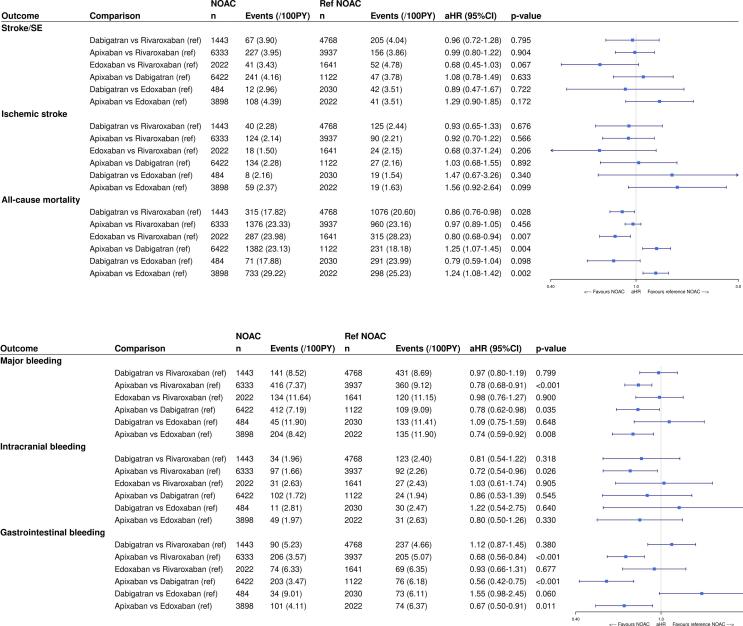
The **A)** effectiveness and **B)** safety compared between individual NOACs types in AF patients with a history of falls after IPTW. The weighted number of subjects at risk in the pseudopopulation, weighted number of events, weighted event rates per 100 PY and aHRs with 95%CIs after IPTW are illustrated. aHR: adjusted hazard ratio; CI: confidence interval; IPTW: inverse probability of treatment weighting; NOAC: non-vitamin K antagonist oral anticoagulant; PY: person-years; Ref: reference category; SE: systemic embolism; vs: versus.

The risks of stroke/SE and ischemic stroke were not significantly different between individual NOACs in patients with a history of falls (eTable 7, Fig. 2). Significantly lower risks of all-cause mortality were observed with dabigatran (aHR 0.86, 95%CI (0.76–0.98), p-value 0.028) and edoxaban (aHR 0.80, 95%CI (0.68–0.94), p-value 0.007) compared to rivaroxaban, whereas significantly higher mortality risks were observed with apixaban compared to dabigatran (aHR 1.25, 95%CI (1.07–1.45), p-value 0.004) and edoxaban (aHR 1.24, 95%CI (1.08–1.42), p-value 0.002).

Significantly lower risks of major bleeding were observed with apixaban compared to dabigatran (aHR 0.78, 95%CI (0.62–0.98), p-value 0.035), rivaroxaban (aHR 0.78, 95%CI (0.68–0.91), p-value < 0.001) and edoxaban (aHR 0.74, 95%CI (0.59–0.92), p-value 0.008), driven by significantly lower gastrointestinal bleeding risks (aHR 0.56, 95%CI (0.42–0.75), p-value < 0.001; aHR 0.68, 95%CI (0.56–0.84), p-value < 0.001; and aHR 0.67, 95%CI (0.50–0.91), p-value 0.011, respectively). Apixaban was also associated with a significantly lower risk of intracranial bleeding compared to rivaroxaban (aHR 0.72 (0.54–0.96), p-value 0.026).

### 3.5 Sensitivity analyses

Trends were consistent with an intention-to-treat approach (mean follow-up of 1.8 (SD 1.5) years; 33,263 person-years), although NOACs were associated with a significantly lower risk of intracranial bleeding compared to VKAs (aHR 0.78 (0.64–0.96), p-value 0.017), and significantly higher gastrointestinal bleeding risks were observed with dabigatran (aHR 1.31 (1.05–1.63), p-value 0.019) and rivaroxaban (aHR 1.23 (1.04–1.45), p-value 0.014) compared to VKAs (eTable 8, eFigure 4). Findings were also consistent when restricting the study population to subjects with an ICD-coded hospital discharge diagnosis of AF (n = 14,980) (eTable 9, eFigure 5) or to subjects having initiated treatment between October 2016 and January 2019 (n = 9618) (eTable 10, eFigure 6).

## 4 Discussion

Main findings of this nationwide cohort study including more than 250,000 AF patients during 328,796 person-years of on-treatment follow-up are: (1) a history of falls in anticoagulated AF patients was an independent risk factor for all-cause mortality, major bleeding, intracranial bleeding and new falls, but not for thromboembolism; (2) NOACs were associated with significantly lower risks of stroke/SE and all-cause mortality, and a similar major bleeding risk compared to VKAs in AF patients with a history of falls; and (3) thromboembolic risks of individual NOACs were comparable, while regarding bleeding risks, apixaban appeared to be associated with less major bleeding in AF patients with a history of falls due to a lower gastrointestinal bleeding risk. Higher observed mortality risks with apixaban compared to dabigatran and edoxaban warrants however caution and further research. To the best of our knowledge, this is the first nationwide cohort study with long-term follow-up investigating the benefit-risk profile of individual NOAC in AF patients with a history of falls, addressing an important research gap [11].

A history of falls, identified in 7.4% of AF patients initiating anticoagulation, is relatively common in real-world patients with AF and is a useful and easy to assess predictor of future falls in clinical practice [8], [13]. Exemplary, in the present study, the annual crude incidence of new falls during follow-up was 15.7% among patients with a history of falls as compared to 4.8% in patients without a history of falls, corresponding with a 63% increased risk of new falls after multivariable adjustment. A history of falls is also a general prognostic factor for adverse outcomes in anticoagulated patients with AF, as illustrated by the 11%, 7% and 30% increased risks of death, major bleeding, and intracranial bleeding respectively, which is in line with previous research [8], [12], [13], [14]. These findings highlight the considerable vulnerability of AF patients with a history of falls. Therefore, patients should be closely monitored and modifiable bleeding risk factors should be tackled [11], [30]. Fall prevention is essential, which should include strength, balance and gait training, use of walking aids, correction of environmental hazards, correction of footwear or structural impairments of the feet, vision assessment and treatment, and supplementation of vitamin D and calcium [11], [31], [32], [33]. Likewise, a thorough medication review as part of a comprehensive geriatric assessment [34] should be performed, to switch or discontinue unnecessary, interacting or contraindicated comedication (e.g., fall-risk-increasing drugs) [11], [30], [35], [36], [37]. Lastly, therapy adherence should be optimized [7].

A history of falls should not be viewed as a general reason to withhold anticoagulation [1], [10], [11]. Although RCTs investigating the net clinical benefit of (N)OACs compared to withholding anticoagulation in AF patients with a history of falls are lacking due to obvious ethical considerations, other studies have demonstrated the overall benefit of anticoagulation in this subgroup [12], [14], [38], [39]. Exemplary, warfarin was associated with a 25% relative risk reduction in the composite outcome of stroke, intracranial haemorrhage and myocardial infarction in AF patients with a CHADS_2_ score of ≥ 2 and a history of falls compared to non-anticoagulated patients [14]. Likewise, when weighing the increased risk for fall-related subdural hematomas against the substantial reduction in ischemic stroke risk among warfarin-treated AF patients as compared to non-anticoagulated patients in a historic Markov decision analytic model, a person would have to fall about 295 times in one year for warfarin not to be the preferred therapy over no anticoagulation [38]. Findings were replicated in a more recent Markov model taking into account the risks of subdural and intracerebral haemorrhage with NOACs and VKAs, illustrating that AF patients ≥ 75 years old would need to fall over 35 (warfarin), 45 (rivaroxaban) and 458 (apixaban) times per year for the net clinical benefit of anticoagulation to be lower than that of no anticoagulation [39]. Therefore, AF patients with a history of falls still appear to benefit from anticoagulation, although an individualized benefit-risk assessment with shared decision making is still essential in each patient [1].

The benefit-risk profile of NOACs was maintained in AF patients with a history of falls, in line with observations from post-hoc analyses of the ARISTOTLE [8] and ENGAGE AF-TIMI 48 trial [12] and one observational cohort study [18]. However, differences in stroke/SE, all-cause mortality or major bleeding risks were not significantly different between NOACs and VKAs in these studies [8], [12], [18] nor after pooling results in meta-analyses [40], [41], due to the limited number of subjects at risk and events. By including a large number of AF patients with a history of falls during long-term follow-up, we could demonstrate that NOACs were associated with a 30%, 41% and 17% significantly reduced risk of stroke/SE, ischemic stroke and all-cause mortality compared to VKAs respectively, while the risks of major and gastrointestinal bleeding were similar, which should be considered as an important contribution to the current evidence and also as a helpful guidance for clinical practice. Regarding the most feared bleeding complication, trends towards lower (non-significantly different) risks of intracranial bleeding with NOACs compared to VKAs were observed in AF patients with a history of falls. However, analyses may have been underpowered due to the low number of events (n = 337). When using an intention-to-treat approach with a longer mean follow-up and higher number of events (n = 578), a 22% significantly lower risk of intracranial bleeding was observed with NOACs compared to VKAs, rendering more reassurance on the preserved benefit of NOACs over VKAs in AF patients with a history of falls.

Apixaban appeared to be associated with less major bleeding among NOACs in AF patients with a history of falls, driven by lower gastrointestinal bleeding risks. Comparable differences in (gastrointestinal) bleeding risks between individual NOACs have been observed in the general AF population [20], [42], [43], [44] and among older geriatric patients with AF [11], [45], [46], [47]. However, significantly higher risks of all-cause mortality were observed with apixaban compared to dabigatran and edoxaban, despite similar thromboembolic and intracranial bleeding risks, and lower major and gastrointestinal bleeding risks [20], [47]. Although confounding by indication was reduced using IPTW, unmeasured confounding and selective prescribing may have impacted results, since apixaban users were older, used more drugs concomitantly and had higher CHA_2_DS_2_-VASc and HAS-BLED scores than other NOAC users (eTable 4), which can be interpreted as a proxy of a more severe, geriatric profile. Therefore, results should be considered as hypothesis-generating and interpreted with caution, while awaiting further evidence.

### 4.1 Strengths and limitations

Strengths of this nationwide cohort study include the long-term follow-up duration up to 6 years, use of an on-treatment analysis to reduce exposure misclassification, and adjustment for several confounders using IPTW.

Several limitations should be acknowledged. First, coding errors and misclassification bias may be present due to the observational design using healthcare databases, although missing data and misclassification of characteristics were reduced by identifying comorbidities based on ICD, medical procedure codes and/or medication prescription claims assessed in ambulatory and hospital care. Second, due to the specific inclusion of AF patients initiating anticoagulation, we could not assess the influence of a history of falls in non-anticoagulated AF patients, nor estimate the net clinical benefit of initiating compared to withholding anticoagulation. Third, although we thoroughly adjusted for confounders, there is a risk of unmeasured confounding due to missing lifestyle characteristics (e.g., weight, smoking) and laboratory values (e.g., renal function, INR). Likewise, (in)appropriate NOAC dosing and time in therapeutic range of VKA users could not be assessed. Fourth, anticoagulant use was assessed based on dispensing data to account for discontinuation or switch of treatment, not on the patients’ actual intake. However, findings were consistent using an intention-to-treat approach. Fifth, the follow-up duration of edoxaban users was considerably shorter than other NOACs due to variable approval dates. Nevertheless, effect estimates were consistent when restricting the study population to subjects having initiated treatment since October 2016. Sixth, data were lacking on the specific causes of death, which would have been of interest to explore why differences in the risk of all-cause mortality between individual NOACs were observed. Lastly, although persons with competing treatment indications were excluded, subjects were not required to have an ICD-coded hospital discharge diagnosis of AF to be included, as this would have limited the study population to hospitalized AF subjects and excluded AF subjects treated exclusively in primary or ambulatory care [20], [25]. Nevertheless, trends were consistent when specifically investigating subjects with an ICD-coded diagnosis of AF ≤ 1 year before or ≤ 90 days after the index date.

## 5 Conclusion

In conclusion, a history of falls was an independent risk factor for major bleeding, intracranial bleeding, all-cause mortality and new falls in anticoagulated AF patients, but not for thromboembolism. Among patients with a history of falls, NOACs were associated with significantly lower risks of stroke/SE and all-cause mortality, and a similar risk of major bleeding compared to VKAs. Although thromboembolic risks were similar, bleeding risks differed between NOACs in patients with a history of falls, as apixaban was associated with a significantly lower risk of major bleeding, driven by lower gastrointestinal bleeding risks. However, more research is needed to reassess findings on the potentially increased mortality risk with apixaban compared to dabigatran and edoxaban in this subgroup.

## Funding

This work was supported by grants from the Research Foundation Flanders (FWO) (Grant number 11C0820N to Maxim Grymonprez). The sponsor had no role in the study design; in the collection, analysis and interpretation of data; and preparation, review, or approval of the manuscript.

## CRediT authorship contribution statement

**Maxim Grymonprez:** Conceptualization, Methodology, Software, Validation, Formal analysis, Investigation, Data curation, Writing – original draft, Visualization. **Mirko Petrovic:** Writing – review & editing. **Tine L. De Backer:** Writing – review & editing. **Stephane Steurbaut:** Writing – review & editing. **Lies Lahousse:** Conceptualization, Methodology, Validation, Investigation, Resources, Data curation, Supervision, Project administration, Funding acquisition.

## Declaration of Competing Interest

The authors declare the following financial interests/personal relationships which may be considered as potential competing interests: ‘Outside this manuscript, TDB has served as a chairperson during a lecture for Bayer and Daiichi Sankyo and participated in an expert meeting for Pfizer. Outside this manuscript, LL has been consulted as expert for AstraZeneca. Outside this manuscript, MP and SS have given a lecture sponsored by BMS, LL a lecture sponsored by Chiesi, and SS, LL and MG lectures sponsored by IPSA vzw, a non-profit organization facilitating lifelong learning for pharmacists. No author has received any fees personally.’
