# Supplementary material for: Clinical outcomes in patients with atrial fibrillation and a history of falls using non-vitamin K antagonist oral anticoagulants: A nationwide cohort study
Source: Int J Cardiol Heart Vasc. 2023 May 18;47:101223. doi: 10.1016/j.ijcha.2023.101223 (PMC10209699; doi:10.1016/j.ijcha.2023.101223)
Supplement: Supplementary data 1 [file mmc1.docx]

**Supplementary materials**

Table of contents

[Supplemental tables 3](#_Toc134110994)

[eTable 1: STROBE reporting guideline 3](#_Toc134110995)

[eTable 2: Definition of in- and exclusion criteria, comorbidities, medication history and clinical risk scores 5](#_Toc134110996)

[eTable 3: Definition of outcomes 13](#_Toc134110997)

[eTable 4: Baseline characteristics of NOAC users with history of falls 14](#_Toc134110998)

[eTable 5: Number of events and crude event rates (main analysis) 15](#_Toc134110999)

[eTable 6: NOAC versus VKA (main analysis) 16](#_Toc134111000)

[eTable 7: NOAC versus NOAC (main analysis) 17](#_Toc134111001)

[eTable 8: Intention-to-treat analysis (sensitivity analysis) 18](#_Toc134111002)

[eTable 9: ICD-coded hospital discharge diagnosis of AF (sensitivity analysis) 20](#_Toc134111003)

[eTable 10: October 1^st^, 2016 – January 1^st^, 2019 (sensitivity analysis) 22](#_Toc134111004)

[Supplemental figures 24](#_Toc134111005)

[eFigure 1: Overview of study design 24](#_Toc134111006)

[eFigure 2: Flowchart of study population 25](#_Toc134111007)

[eFigure 3: Love plots (main analysis) 26](#_Toc134111008)

[eFigure 4: Forest plot (intention-to-treat analysis) 32](#_Toc134111009)

[eFigure 5: Forest plot (patients with ICD-coded hospital discharge diagnosis of AF) 34](#_Toc134111010)

[eFigure 6: Forest plot (October 1^st^, 2016 – January 1^st^, 2019) 36](#_Toc134111011)

[References 38](#_Toc134111012)

# Supplemental tables

## eTable 1: STROBE reporting guideline

|  | **Item No.** | **Recommendation** | **Page No.** |
| --- | --- | --- | --- |
| **Title and abstract** | 1 | (*a*) Indicate the study’s design with a commonly used term in the title or the abstract | 1-2 |
|  |  | (*b*) Provide in the abstract an informative and balanced summary of what was done and what was found | 2 |
| **Introduction** | | |  |
| Background/rationale | 2 | Explain the scientific background and rationale for the investigation being reported | 3-4 |
| Objectives | 3 | State specific objectives, including any prespecified hypotheses | 3-4 |
| **Methods** | | |  |
| Study design | 4 | Present key elements of study design early in the paper | 5-6 |
| Setting | 5 | Describe the setting, locations, and relevant dates, including periods of recruitment, exposure, follow-up, and data collection | 5-6 |
| Participants | 6 | (*a*) Give the eligibility criteria, and the sources and methods of selection of participants. Describe methods of follow-up | 5-6 |
|  |  | (*b*) For matched studies, give matching criteria and number of exposed and unexposed | 5-6 |
| Variables | 7 | Clearly define all outcomes, exposures, predictors, potential confounders, and effect modifiers. Give diagnostic criteria, if applicable | 6-7, eTable 2-3 |
| Data sources/ measurement | 8* | For each variable of interest, give sources of data and details of methods of assessment (measurement). Describe comparability of assessment methods if there is more than one group | 6-7 |
| Bias | 9 | Describe any efforts to address potential sources of bias | 7-8 |
| Study size | 10 | Explain how the study size was arrived at | 5-6 |
| Quantitative variables | 11 | Explain how quantitative variables were handled in the analyses. If applicable, describe which groupings were chosen and why | 7-8 |
| Statistical methods | 12 | (*a*) Describe all statistical methods, including those used to control for confounding | 7-8 |
|  |  | (*b*) Describe any methods used to examine subgroups and interactions | 7-8 |
|  |  | (*c*) Explain how missing data were addressed | 7-8 |
|  |  | (*d*) If applicable, explain how loss to follow-up was addressed | / |
|  |  | (*e*) Describe any sensitivity analyses | 8 |
| **Results** | | |  |
| Participants | 13* | (a) Report numbers of individuals at each stage of study—eg numbers potentially eligible, examined for eligibility, confirmed eligible, included in the study, completing follow-up, and analysed | 9, Table 1, eTable 4 |
|  |  | (b) Give reasons for non-participation at each stage | / |
|  |  | (c) Consider use of a flow diagram | Figure 1 |
| Descriptive data | 14* | (a) Give characteristics of study participants (eg demographic, clinical, social) and information on exposures and potential confounders | Table 1, eTable 4 |
|  |  | (b) Indicate number of participants with missing data for each variable of interest | Table 1 |
|  |  | (c) Summarise follow-up time (eg, average and total amount) | Table 1, eTable 4 |
| Outcome data | 15* | Report numbers of outcome events or summary measures over time | 9, Table 2 |
| Main results | 16 | (*a*) Give unadjusted estimates and, if applicable, confounder-adjusted estimates and their precision (eg, 95% confidence interval). Make clear which confounders were adjusted for and why they were included | 9-11, Table 3, eTable 5-6, Figure 2-3 |
|  |  | (*b*) Report category boundaries when continuous variables were categorized | 9-11, Table 1 |
|  |  | (*c*) If relevant, consider translating estimates of relative risk into absolute risk for a meaningful time period | / |
| Other analyses | 17 | Report other analyses done—eg analyses of subgroups and interactions, and sensitivity analyses | 11, eTable 7-9, eFigure 3-5 |
| **Discussion** | | |  |
| Key results | 18 | Summarise key results with reference to study objectives | 12-14 |
| Limitations | 19 | Discuss limitations of the study, taking into account sources of potential bias or imprecision. Discuss both direction and magnitude of any potential bias | 14-15 |
| Interpretation | 20 | Give a cautious overall interpretation of results considering objectives, limitations, multiplicity of analyses, results from similar studies, and other relevant evidence | 12-14 |
| Generalisability | 21 | Discuss the generalisability (external validity) of the study results | 12-14 |
| **Other information** | | |  |
| Funding | 22 | Give the source of funding and the role of the funders for the present study and, if applicable, for the original study on which the present article is based | 17 |

*Give information separately for exposed and unexposed groups.

**eTable 1:** Compliance to the STROBE (Strengthening the Reporting of Observational Studies in Epidemiology) reporting guideline.^1^

## eTable 2: Definition of in- and exclusion criteria, comorbidities, medication history and clinical risk scores

| **VARIABLE** | | **ICD, ATC AND MEDICAL PROCEDURE CODES** |
| --- | --- | --- |
| **INCLUSION CRITERIA** | | |
| OAC use (NOAC or VKA) | | **ATC:** B01AE07, B01AF01, B01AF02, B01AF03, B01AA03, B01AA04, B01AA07 |
| ≥45 years | | Age ≥45 years on index date |
| ≥1 year coverage | | ≥1 year coverage by a Belgian health insurance fund |
| History of falls | | **ICD-9:** E804, E833, E834, E835, E843, E880-E886, E888, E917.5-E917.9, E987, V15.88  **ICD-10:** R29.6, V00.141, V00.811, V00.831, V81.5, V81.6, V82.5, V82.6, V92.0, V93.3, V94.0, V97.0, W00, W01, W03, W05-W15, W16.0-W16.4, W17, W18, W19, Y21.1, Y21.3, Y30, Z91.81 |
| Atrial fibrillation (sensitivity analysis) | | **ICD 9:** 427.3  **ICD 10:** I48 |
| **EXCLUSION CRITERIA** | | |
| Recent total hip/knee replacement surgery | | **Medical procedure group code:** N32 (≤6 months before index date) |
| Recent venous thromboembolism  (deep vein thrombosis or pulmonary embolism) | | **ICD-9:** 451.1, 451.2, 451.81, 451.89, 451.9, 452, 453.2, 453.3, 453.4, 453.5, 453.77, 453.79, 453.87, 453.89, 671.3, 671.4 (≤6 months before index date)  **ICD-10:** I80.1, I80.2, I80.3, I80.8, I80.9, I81, I82.2, I82.3, I82.4, I82.5, I82.89, I82.9, O22.3, O22.5, O87.1, O87.3 (≤6 months before index date) |
| Valvular atrial fibrillation | |  |
|  | Moderate-severe mitral stenosis | **ICD-9:** 394.0, 394.2, 396.0, 396.1, 746.5  **ICD-10:** I05.0, I05.2, I34.2, Q23.2 |
|  | Mechanical prosthetic heart valve | **ICD-9:** V43.3  **ICD-10:** Z95.2  **Medical procedure code:** 159110, 159121, 159132, 159143, 159154, 159165 |
| End-stage renal disease | |  |
|  | CKD stage V (without dialysis) | **ICD-9:** 403.01, 403.11, 403.91, 404.02, 404.12, 404.92, 585.5, 585.6, 586  **ICD-10:** N18.5, N18.6, N19, I12.0, I13.11 |
|  | Dialysis | **ICD-9:** V45.11, V56  **ICD-10:** Z49, Z99.2  **Medical procedure group code:** N81 |
| **DEMOGRAFICS (on index date)** | | |
| Age | | Age on index date based on the year and month of birth, not the exact date due to patient privacy. |
| Sex | | Sex on index date |
| **COMORBIDITIES (≤1 year before index date)**^2-4^ | | |
| Hypertension | | **ICD-9:** 401-405, 437.2  **ICD-10:** I10-I13, I15, I16, I67.4  **ATC:** combination treatment with ≥2 of the following drug classes:  **I) Cardioselective beta blocker:** C07AB, C07AG, C07BB, C07BG, C07CB, C07CG, C07DB, C07FB, C07FX03, C07FX04, C07FX05, C07FX06, C09BX02, C09BX04, C09BX05, C09DX05  **II) ACE inhibitor or angiotensin II receptor blocker:** C09A, C09B, C09C, C09D, C10BX04, C10BX06, C10BX07, C10BX10, C10BX11, C10BX12, C10BX13, C10BX14, C10BX15, C10BX16, C10BX17, C10BX18  **III) Calcium channel blocker:** C07FB, C08C, C08G, C09BB, C09DB, C09BX01, C09BX03, C09BX04, C09DX01, C09DX03, C09DX06, C09DX07, C09XA53, C09XA54, C10BX03, C10BX07, C10BX09, C10BX11, C10BX14, C10BX18  **IV) Non-loop diuretic:** C02L, C03A, C03BA, C03BB, C03EA, C07B, C07C, C07D, C08GA, C09BA, C09BX01, C09BX03, C09DA, C09DX01, C09DX03, C09DX06, C09DX07, C09XA52, C09XA54, C10BX13, C03D, C03EA, C03EB  **V) Other antihypertensive (alpha adrenergic blocker, vasodilator)**: C02A, C02B, C02C, C02DB, C02DD, C02DG, C02L |
| Coronary artery disease  (stable or recent myocardial infarction) | | **ICD-9:** 410, 411, 412, 413, 414, 429.2, 429.7, V45.81, V45.82  **ICD-10:** I20, I21, I22, I23, I24, I25, Z95.1, Z95.5, Z98.61  **Medical procedure code:** 158992, 159003, 159014, 159025, 159036, 159040, 229515, 229526, 229574, 229585, 229611, 229622, 229633, 229644, 589013, 589024, 589153, 589164, 589934, 589945, 589956, 589960, 680315, 680326, 680352, 680363, 687875, 687886 |
| Congestive heart failure | | **ICD-9:** 398.91, 402.01, 402.11, 402.91, 404.01, 404.03, 404.11, 404.13, 404.91, 404.93, 425.4–425.9, 428  **ICD-10:** I09.81, I11.0, I13.0, I13.2, I42.0, I42.6-I42.9, I43, I50  **ATC:** combination treatment of all of the following drug classes:  **I) Cardioselective beta blocker:** C07AB, C07AG, C07BB, C07BG, C07CB, C07CG, C07DB, C07FB, C07FX03, C07FX04, C07FX05, C07FX06, C09BX02, C09BX04, C09BX05, C09DX05  **II) ACE inhibitor, angiotensin II receptor blocker or ARNI:** C09A, C09B, C09C, C09D, C10BX04, C10BX06, C10BX07, C10BX10, C10BX11, C10BX12, C10BX13, C10BX14, C10BX15, C10BX16, C10BX17, C10BX18  **III) Potassium-sparing diuretic:** C03D, C03EA, C03EB  **IV) Loop diuretic:** C03C, C03EB |
| Valvular heart disease  (aortic, mitral or other/non-specific valve disease, valve repair, bioprosthetic heart valve) *(except for moderate-severe mitral stenosis or mechanical prosthetic heart valve)* | | **ICD 9:** 036.42, 074.22, 093.2, 098.84, 112.81, 391.1, 394.1, 394.2, 394.9, 395.0, 395.1, 395.2, 395.9, 396, 397, 421, 424.0, 424.1, 424.2, 424.3, 424.9, 746.0, 746.1, 746.3, 746.4, 746.6, V42.2  **ICD 10:** A32.82, A39.51, A52.03, A54.83, B33.21, B37.6, I01.1, I05.1, I05.2, I05.8, I05.9, I06.0, I06.1, I06.2, I06.8, I06.9, I07, I08, I09.1, I09.89, I33, I34.0, I34.1, I34.8, I34.9, I35.0, I35.1, I35.2, I35.8, I35.9, I36, I37, I38, I39, M32.11, Q22, Q23.0, Q23.1, Q23.3, Q23.8, Q23.9, Z95.3, Z95.4  **Medical procedure code:** 159176, 159180, 159191, 159202, 159213, 159224, 159235, 159246, 159250, 159261, 159272, 159283, 159294, 159305, 170634, 170645, 172491, 172502, 172513, 172524, 172734, 172745, 172756, 172760, 172771, 172782, 172955, 172966, 172970, 172981, 172992, 173003, 229515, 229526, 229596, 229600, 589190, 589201, 680153, 680164, 680175, 680186, 680993, 681004, 684736, 684740, 688192, 688203, 691950, 691961, 704616, 704620, 704631, 704642, 704653, 704664 |
| Peripheral artery disease | | **ICD-9:** 440, 441, 443.89, 443.9, 444, 447.1, 557.1, 557.9, V43.4  **ICD-10:** I70, I71, I73.8, I73.9, I74, I77.1, I79.0, K55.1, K55.8, K55.9, Z95.82, Z98.62  **Medical procedure code:** 229294, 229305, 229316, 229320, 229331, 229342, 235071, 235082, 235093, 235104, 235115, 235126, 235196, 235200, 235211, 235222, 236014, 236025, 236036, 236040, 236051, 236062, 237016, 237020, 237031, 237042, 237053, 237064, 237075, 237086, 237090, 237101, 237171, 237182, 589050, 589061, 589094, 589105, 589175, 589186, 589595, 589606, 589610, 589621, 589632, 589643, 589654, 589665 |
| Dyslipidemia | | **ICD-9:** 272.0, 272.1, 272.3, 272.4, 272.5, 272.8, 272.9  **ICD-10:** E78  **ATC:** C10 |
| Chronic kidney disease  (CKD stage III-IV, renal transplant, other/non-specific CKD) *(except for CKD stage V or dialysis)* | | **ICD-9:** 249.4, 250.4, 403.00, 403.10, 403.90, 404.00, 404.10, 404.90, 580, 581, 582, 583, 584, 585.3, 585.4, 585.9, 586, 588, 590.0, 753.12-753.15, 996.81, V42.0  **ICD-10:** E08.2, E09.2, E10.2, E11.2, E13.2, I12.9, I13.10, M32.14, M32.15, N00, N01, N02, N03, N04, N05, N07, N11, N14, N15.0, N17, N18.3, N18.4, N18.9, N19, N25, Q61.1-Q61.4, T86.1, Z94.0  **Medical procedure code:** 107096, 107111, 107133, 107155, 318010, 318021, 318290, 318301, 754294, 757433, 757492 |
| Chronic liver disease  (mild, moderate-severe, cirrhosis) | | **ICD-9:** 070.0, 070.2, 070.3, 070.4, 070.51, 070.52, 070.54, 070.6, 070.70, 070.71, 155.0, 155.1, 155.2, 197.7, 456.0, 456.1, 456.2, 567.23, 570, 571, 571.2, 571.5, 571.6, 572.2, 572.3, 572.4, 572.8, 573.0, 573.5, 573.8, 573.9, 789.59, V42.7  **ICD-10:** B15.0, B16.0, B16.2, B17.0, B17.10, B17.11, B18, B19.0, B19.10, B19.11, B19.20, B19.21, C22, C78.7, I85, I86.4, K65.2, K70.0, K70.1, K70.2, K70.3, K70.4, K70.9, K71.1, K71.3, K71.4, K71.5, K71.6, K71.7, K71.8, K71.9, K72, K73, K74, K75.3, K75. 4, K75.8, K75.9, K76.0, K76.1, K76.2, K76.5, K76.6, K76.7, K76.81, K76.89, K76.9, K77, R18.8, Z94.4  **ATC:** J05AB04, J05AF05, J05AF07, J05AF08, J05AF10, J05AE11, J05AE12, J05AE14, J05AX15, J05AX65, J05AP  **Medical procedure code:** 318076, 318080, 318334, 318345, 472113, 472124, 556754, 556765, 589352, 589363 |
| Chronic lung disease  (COPD, asthma, other) | | **ICD-9**: 416, 491-496, 500-505, 506.4, 508.1, 515, 516.3, 516.9, 518.1, 518.2, 518.83, 518.84  **ICD-10**: I27, J41-J45, J47, J60-J67, J68.4, J70.1, J70.3, J84.1, J84.9, J96.1, J96.2, J98.2, J98.3  **ATC:** R03DC, R03DX |
| Obstructive sleep apnea | | **ICD-9:** 327.23  **ICD-10**: G47.33  **Medical procedure code:** 765951, 779870, 779881, 779892, 779903, 779914, 779925, 779936, 779951, 788012, 788023 |
| Cancer | | **ICD-9:** 140-209, 223, 230-239, 258.0, V58.0, V58.11, V58.12  **ICD-10:** C00-C96, D00-D09, D37-D49, E31.2, Z51.0, Z51.11, Z51.12  **ATC:** L01  **Medical procedure code:** 154873, 154884, 154895, 154906, 157231, 157242, 201191, 201202, 201213, 201224, 220275, 220286, 220371, 220382, 201213, 201224, 226914, 226925, 226936, 226940, 227216, 227220, 227275, 227286, 227636, 227640, 227651, 227662, 227673, 227684, 227695, 227706, 227710, 227721, 227732, 227743, 227754, 227765, 227776, 227780, 227791, 227802, 227813, 227824, 227835, 227846, 228012, 228023, 228174, 228185, 228233, 228244, 228255, 228266, 228270, 228281, 228292, 228303, 228314, 228325, 228336, 228340, 230473, 230484, 231033, 231044, 241231, 241242, 241415, 241426, 241430, 241441, 241452, 241463, 241555, 241566, 242012, 242023, 242034, 242045, 242292, 242303, 242314, 242325, 242830, 242841, 242852, 242863, 242874, 242885, 242896, 242900, 243051, 243062, 243073, 243084, 243235, 243246, 243736, 243740, 243751, 243762, 243773, 243784, 244016, 244020, 244031, 244042, 244075, 244086, 244790, 244801, 244856, 244860, 244893, 244904, 244915, 244926, 244930, 244941, 244952, 244963, 244974, 244985, 245512, 245523, 245534, 245545, 246050, 246061, 246072, 246083, 247111, 247122, 247133, 247144, 251753, 251764, 251775, 251786, 254892, 254903, 256115, 256126, 256336, 256340, 256572, 256583, 257191, 257202, 258355, 258366, 258370, 258381, 258392, 258403, 258451, 258462, 258554, 258565, 258856, 258860, 258871, 258882, 258893, 258904, 259033, 259044, 259114, 259125, 260190, 260201, 260411, 260422, 260433, 260444, 260551, 260562, 260654, 260665, 260750, 260761, 261111, 261122, 261391, 261402, 261472, 261483, 261671, 261682, 261774, 261785, 261796, 261800, 262334, 262345, 262570, 262581, 277756, 277760, 277771, 277782, 278795, 278806, 278810, 278821, 281831, 281842, 281956, 281960, 282310, 282321, 282671, 282682, 284056, 284060, 288455, 288466, 288470, 288481, 289892, 289903, 291056, 291060, 310494, 310505, 311312, 311323, 312550, 312561, 312572, 312583, 312594, 312605, 312653, 312664, 312970, 312981, 350114, 350125, 350136, 350140, 350276, 350280, 350291, 350302, 350372, 350383, 350674, 350685, 350696, 350700, 431174, 431185, 431336, 431340, 431351, 431362, 432294, 432305, 444113, 444124, 444135, 444146, 444150, 444161, 444172, 444183, 444194, 444205, 444216, 444220, 444231, 444242, 444253, 444264, 444275, 444286, 444290, 444301, 444312, 444323, 444334, 444345, 444474, 444485, 444592, 444603, 473970, 473981, 474795, 474806, 565073, 565084, 565095, 565106, 565110, 565121, 565132, 565143, 565154, 565165, 587834, 587845, 587871, 587882, 587893, 587904, 587915, 587926, 588431, 588442, 588453, 588464, 588475, 588486, 588490, 588501, 588512, 588523, 588534, 588545, 588556, 588560, 588571, 588582, 588593, 588604, 588770, 588781, 588976, 588980, 589691, 589702, 589713, 589724, 589831, 589842, 589875, 589886, 594016, 594020, 594031, 594042, 594053, 594064, 594075, 594086, 594090, 594101, 594112, 594123, 594252, 594263, 594274, 594285, 594296, 594300, 594311, 594322, 594333, 594344, 594355, 594366, 594370, 594381, 594392, 594403, 594414, 594425, 594436, 594440, 594451, 594462, 594495, 594506, 594510, 594521, 594532, 594543, 594554, 594565, 594576, 594580, 594591, 594602, 594613, 594624, 594635, 594646, 594694, 594705, 594716, 594720, 594753, 594764, 594775, 594786, 594790, 594801, 594812, 594823, 594834, 594845, 594856, 594860, 594871, 594882, 594893, 594904, 594915, 594926, 594930, 594941, 598581, 682636, 682640, 682732, 682743, 687934, 687945, 698051, 698062, 698095, 698106, 698390, 698401, 698456, 698460, 698471, 698482, 698493, 698504, 698530, 698541, 745010, 745021, 745032, 745043, 745113, 745124, 745135, 745146, 745150, 745161 |
| Upper gastrointestinal tract disorder (gastroesophageal reflux disease or peptic ulcer disease) | | **ICD-9:** 041.86, 530.1, 530.2, 530.81, 530.85, 531, 532, 533, 534, 535, V12.71  **ICD-10:** B96.81, K20, K21, K22.1, K22.7, K25, K26, K27, K28, K29, Z87.11  **ATC:** A02BD04, A02BD11  **Medical procedure code:** 172616, 172620, 172631, 172642, 172653, 172664, 474854, 474865, 550093, 550104, 552370, 552381 |
| Lower gastrointestinal tract disorder (polyposis, diverticulosis, angiodysplasia, hemorrhoids) | | **ICD-9:** 211.3, 211.4, 448.0, 455, 537.82, 537.83, 537.84, 562, 569.84, 569.85, 569.86, V12.72  **ICD-10:** D12, I78.0, K31.81, K31.82, K55.2, K57, K63.5, K64, K63.81, Z86.010  **Medical procedure code:** 112313, 112324, 243294, 243305, 244311, 244322, 244355, 244366, 244370, 244381, 244554, 244565, 244576, 244580, 244591, 244602, 472150, 472161, 473211, 473222, 473476, 473480, 473675, 473686, 473955, 473966, 473970, 473981, 474795, 474806 |
| Diabetes mellitus | | **ICD-9:** 249, 250, 357.2, 362.0, 366.41, V45.85, V53.91, V65.46  **ICD-10:** E8, E9, E10, E11, E13, Z46.81, Z96.41  **ATC:** A10  **Medical procedure code:** 102852, 107015, 107030, 107052, 107074, 109594, 174370, 174381, 174392, 174403, 174414, 174425, 174436, 174440, 174451, 174462, 174473, 174484, 174495, 174506, 174510, 174521, 653671, 653682, 697093, 697104, 754176, 754191, 754250, 754272, 754736, 757352, 757374, 757396, 757411, 757514, 757536, 757551, 770070, 773393, 773496, 784630, 784641, 784652, 784663, 785735, 785750, 785772, 785794, 785816, 785831, 785853, 785875, 785890, 785912, 785934, 785956, 786015, 786030, 786100, 788756, 788771, 788793, 788815, 788830, 788852, 788874, 788896, 788911, 788933, 788955, 789751, 789773, 789795, 789810, 789832, 789854, 789876, 789891, 789913, 789935, 794032, 794113, 794135, 794150, 794194, 794216, 794231, 794253, 794275, 794290, 794312, 794334, 794356, 794371, 794393, 794415, 794430, 794452, 961295, 961306, 961332, 961343 |
| Anemia | | **ICD-9:** 280-285  **ICD-10:** D46.0-D46.4, D50-D53, D56-D64 |
| Dementia  (Alzheimer’s disease, vascular, other) | | **ICD-9:** 046.11, 046.19, 290.0, 290.1, 290.2, 290.3, 290.4, 291.1, 294, 331.0, 331.11, 331.19, 331.82  **ICD-10:** A81.0, F01, F02, F03, F10.27, F10.97, G30, G31.0, G31.83, G31.85  **ATC:** N06D |
| Frailty | | John Hopkins Claims-based Frailty Indicator^5^ (see below) |
| Prior stroke or systemic embolism | | **ICD-9:** 431, 432.9, 433.01, 433.1, 433.21, 433.31, 433.81, 433.91, 434.01, 434.11, 434.91, 436, 438, 444, 557.0, 593.81, V12.54  **ICD-10:** D73.5, I61, I62.9, I63.0, I63.1, I63.2, I63.3, I63.4, I63.5, I63.8, I63.9, I67.89, I69.1, I69.2, I69.3, I69.8, I69.9, I74, K55.01, K55.02, N28.0, Z86.73  **Medical procedure code:** 182136, 182140, 182151, 182162, 182173, 182184, 235130, 235141, 237112, 237123, 477724, 477746, 477761, 477783, 589175, 589186 |
| Prior major or clinically relevant non-major bleeding | | **ICD-9:** 285.1, 287.8, 287.9, 336.1, 360.43, 362.43, 362.81, 363.6, 363.72, 364.41, 372.72, 376.32, 377.42, 379.23, 388.69, 423.0, 430, 431, 432.0, 432.1, 432.9, 455.8, 456.0, 456.20, 459.0, 530.21, 530.7, 530.82, 531.00, 531.20, 531.40, 531.60, 532.00, 532.20, 532.40, 532.60, 533.00, 533.20, 533.40, 533.60, 534.00, 534.20, 534.40, 534.60, 535.01, 535.11, 535.21, 535.31, 535.41, 535.51, 535.61, 535.71, 537.83, 537.84, 562.02, 562.03, 562.12, 562.13, 568.81, 569.3, 569.85, 569.86, 578.0, 578.1, 578.9, 596.7, 599.7, 602.1, 620.7, 621.4, 623.6, 626.2, 626.5, 626.6, 626.7, 626.8, 626.9, 627.0, 627.1, 719.1, 729.92, 784.7, 784.8, 786.30, 786.39, 852.0, 852.2, 852.4, 853.0, 958.2  **ICD-10:** D62, D68.32, D69.8, D69.9, G95.19, H05.23, H11.3, H21.0, H31.3, H31.41, H35.6, H35.73, H43.1, H44.81, H47.02, H92.2x, I23.0, I31.2, I60, I61, I62.0, I62.1, I62.9, I85.01, I85.11, J94.2, J95.01, K22.11, K22.6, K22.8, K25.0, K25.2, K25.4, K25.6, K26.0, K26.2, K26.4, K26.6, K27.0, K27.2, K27.4, K27.6, K28.0, K28.2, K28.4, K28.6, K29.01, K29.21, K29.31, K29.41, K29.51, K29.61, K29.71, K29.81, K29.91, K31.811, K31.82, K50.011, K50.111, K50.811, K50.911, K51.011, K51.211, K51.311, K51.411, K51.511, K51.811, K51.911, K55.21, K57.01, K57.11, K57.13, K57.21, K57.31, K57.33, K57.41, K57.51, K57.53, K57.81, K57.91, K57.93, K62.5, K63.81, K64.9, K66.1, K92.0, K92.1, K92.2, K94.01, K94.11, K94.21, K94.31, M25.0, M79.81, N02, N30.01, N30.11, N30.21, N30.31, N30.41, N30.81, N30.91, N42.1, N83.6, N83.7, N85.7, N89.7, N92.0, N92.1, N92.3, N92.4, N93.0, N93.8, N93.9, N95.0, N99.510, N99.520, N99.530, R04.0, R04.1, R04.2, R04.89, R04.9, R31, R58, S06.340A, S06.341A, S06.342A, S06.343A, S06.344A, S06.345A, S06.346A, S06.347A, S06.348A, S06.349A, S06.350A, S06.351A, S06.352A, S06.353A, S06.354A, S06.355A, S06.356A, S06.357A, S06.358A, S06.359A, S06.360A, S06.361A, S06.362A, S06.363A, S06.364A, S06.365A, S06.366A, S06.367A, S06.368A, S06.369A, S06.4X0A, S06.4X1A, S06.4X2A, S06.4X3A, S06.4X4A, S06.4X5A, S06.4X6A, S06.4X7A, S06.4X8A, S06.4X9A, S06.5X0A, S06.5X1A, S06.5X2A, S06.5X3A, S06.5X4A, S06.5X5A, S06.5X6A, S06.5X7A, S06.5X8A, S06.5X9A, S06.6X0A, S06.6X1A, S06.6X2A, S06.6X3A, S06.6X4A, S06.6X5A, S06.6X6A, S06.6X7A, S06.6X8A, S06.6X9A, T79.2  **Medical procedure code:** 144605, 144620, 144642, 144664, 144686, 227441, 230403, 230425, 230440, 243600, 254940, 255242, 257445, 431620, 431944, 472124, 473686, 473782 |
| **MEDICATION HISTORY (≤6 months before index date)^4^** | | |
| Cardioselective beta-blockers | | **ATC:** C07AB, C07AG, C07BB, C07BG, C07CB, C07CG, C07DB, C07FB, C07FX03, C07FX04, C07FX05, C07FX06, C09BX02, C09BX04, C09BX05, C09DX05 |
| Verapamil, diltiazem | | **ATC:** C08D |
| Digoxin | | **ATC:** C01AA05, C01AA08 |
| Class I AAD | | **ATC:** C01BA, C01BB, C01BC |
| Class III AAD | | **ATC:** C01BD, C07AA07, C07BA07, C07FX02 |
| Acetylsalicylic acid | | **ATC:** B01AC56, B01AC06, C07FX02, C07FX03, C07FX04, C10BX01, C10BX02, C10BX04, C10BX05, C10BX06, C10BX08, C10BX12 |
| P2Y12-inhibitor | | **ATC:** B01AC04, B01AC05, B01AC22 , B01AC24 , B01AC25 |
| Proton pump inhibitor | | **ATC:** A02BC |
| NSAID | | **ATC:** M01AA, M01AB, M01AC, M01AE, M01AG, M01AH, N02AJ08, N02AJ14, N02AJ19, C08CA51 |
| Oral corticosteroid | | **ATC:** H02AB, H02BX01 |
| SSRI/SNRI | | **ATC**: N06AB, N06AX16, N06AX21, N06CA03 |
| **CLINICAL RISK SCORES** | | |
| CHA_2_DS_2_-VASc score^6^ | | - **Congestive heart failure:** 1 point (definition mentioned above: ‘Congestive heart failure’)  - **Hypertension**: 1 point (definition mentioned above: ‘Hypertension’)  - **Age ≥75 years**: 2 point  - **Diabetes mellitus**: 1 point (definition mentioned above: ‘Diabetes mellitus’)  - **Stroke or systemic embolism**: 2 point (definition mentioned above: ‘Thromboembolism (stroke and/or systemic embolism)’)  - **Vascular disease**: 1 point (definition mentioned above: ‘Coronary artery disease’ & ‘Peripheral artery disease’)  - **Age 65-74 years**: 1 point  - **Sex category (female)**: 1 point |
| HAS-BLED score^6^ | | - **Hypertension**: 1 point (definition mentioned above: ‘Hypertension’)  - **Abnormal renal function**: 1 point (definition mentioned above: ‘Chronic kidney disease’)  - **Abnormal liver function**: 1 point (definition mentioned above: ‘Chronic liver disease’)  - **Stroke**: 1 point (definition mentioned above: ‘Stroke’)  - **Bleeding history or predisposition**: 1 point (definition mentioned above: ‘Prior major or clinically relevant non-major bleeding’)  - **Labile** INR: not available  - **Elderly (>65 years or frailty):** 1 point  - **Drugs (antiplatelet, NSAID):** 1 point (definition mentioned above: ‘Antiplatelet’ & ‘NSAID’)  - **Excessive alcohol drinking**: 1 point:   - **ICD-9:** 265.1, 291, 303, 305.0, 357.5, 425.5, 535.3, 571.0-571.3, 980.0, E860.1, V11.3 - **ICD-10:** E51, F10, G31.2, G62.1, G72.1, I42.6, K29.2, K70, K85.2, K86, O35.4, T51.0, T51.9, Z71.4 - **ATC:** N07BB - **Medical procedure code:** 790090 |
| Frailty (John Hopkins Claims-based Frailty Indicator^5^) | | - **Impaired mobility**: beta coefficient 1.24:   - **ICD-9:** 334, 719.7, 781.2, V46.3, V49.84, V57.81 - **ICD-10:** G11, G32.81, M62.3, R26, R29.6, Z74.01, Z74.09, Z99.3 - **Medical procedure code:** N83, 643451, 643462, 653656, 653660, 770394, 770405, 770416, 770420   - **Depression**: beta coefficient 0.54:   - **ICD-9**: 293.83, 296, 300.4, 301.12, 309.0, 309.1, 309.28, 311 - **ICD-10**: F06.31, F06.32, F30, F31, F32, F33, F34.1, F43.21, F43.23 - **ATC:** N06A   - **Congestive heart failure**: beta coefficient 0.50: (definition mentioned above: ‘Congestive heart failure’)  - **Parkinson’s disease**: beta coefficient 0.50: (definition mentioned above: ‘Parkinson’s disease’)  - **White race**: beta coefficient -0.49: not available  - **Arthritis (any type):** beta coefficient 0.43:   - **ICD-9:** 099.3, 696.0, 711.1, 711.3, 713.1, 714.0, 714.1, 714.2, 714.3, 714.4, 714.8, 714.9, 716.5, 716.6, 720.0, 720.2, 720.89, 720.9, V13.4 - **ICD-10:** L40.5, M02.1, M02.3, M05, M06, M07, M08, M13.0, M13.1, M45, M46.1, M46.8, M46.9, Z87.39 - **ATC:** L04AA13, L04AA24, L04AA29, L04AA37 - **Medical procedure code:** 478030, 478041   - **Cognitive impairment**: beta coefficient 0.33:   - **ICD-9:** 331.2, 331.83, 331.89, 331.9, 797 - **ICD-10:** G31.1, G31.84, G31.89, G31.9, R41.81   - **Charlson comorbidity index** **(> 0)**: beta coefficient 0.31  - **Stroke**: beta coefficient 0.28: (definition mentioned above: ‘Stroke’)  - **Paranoia**: beta coefficient 0.24:   - **ICD-9:** 293.81, 293.82, 295, 297, 298 - **ICD-10:** F06.0, F06.2, F20, F22, F23, F24, F28, F29   - **Chronic skin ulcer**: beta coefficient 0.23:   - **ICD-9:** 707 - **ICD-10:** E08.621, E08.622, E09.621, E09.622, E10.621, E10.622, E11.621, E11.622, E13.621, E13.622, L89, L97, L98.4 - **Medical procedure code:** 114074, 114085   - **Pneumonia**: beta coefficient 0.21:   - **ICD-9:** 480, 481, 482, 483, 484, 485, 486, 487.0 - **ICD-10:** A48.1, J11.0, J12, J13, J14, J15, J16, J17, J18   - **Male sex**: beta coefficient -0.19  - **Skin and soft tissue infection**: beta coefficient 0.18:   - **ICD-9:** 680, 681, 682, 683, 684, 685, 686, 695.81 - **ICD-10:** L00, L01, L02, L03, L04, L05, L08   - **Mycoses**: beta coefficient 0.14:   - **ICD-9:** 110, 111, 112, 114, 115, 116, 117, 118 - **ICD-10:** B35, B36, B37, B38, B39, B40, B41, B42, B43, B44, B45, B46, B47, B48, B49   - **Age (in 5 year categories)**: beta coefficient 0.09  - **Admission in past 6 months**: beta coefficient 0.09  - **Gout or other crystal-induced arthropathy**: beta coefficient 0.08:   - **ICD-9:** 274, 712.3, 712.8, 712.90 - **ICD-10:** M10, M11, M1A - **ATC:** M04A   - **Falls**: beta coefficient 0.08: (definition mentioned above: ‘History of falls’)  - **Musculoskeletal problems**: beta coefficient 0.05:   - **ICD-9:** 713, 716.0, 716.2, 716.3, 716.4, 716.5, 716.6, 716.8, 716.9, 718.1, 718.2, 718.5, 718.6, 718.7, 718.8, 718.9, 719-724, 733.0, 733.1, 733.93, 733.94, 733.95, 733.96, 733.97, 733.98, V13.51, V13.52 - **ICD-10:** M07, M12.0, M12.1, M12.2, M12.3, M12.4, M12.8, M12.9, M13, M14, M24.0, M24.3, M24.6, M24.7, M24.8, M24.9, M25, M45, M46.0, M46.1, M46.4, M46.8, M46.9, M47-M51, M53, M54, M80, M81, M84.3, M84.4, M84.5, M84.6, Z87.31   - **Urinary tract infection**: beta coefficient 0.05:   - **ICD-9:** 590.1, 590.8, 590.9, 595.0, 595.4, 595.89, 595.9, 597, 599.0 - **ICD-10:** N30.0, N30.8, N30.9, N10, N12, N13.6, N15.9, N16, N34, N39.0   **ATC:** J01XE01, J01XX01 |
| Charlson Comorbidity Index^7,8^ | | - **Myocardial infarction:** 1 point:   - **ICD-9:** 410.00, 410.01, 410.10, 410.11, 410.20, 410.21, 410.30, 410.31, 410.40, 410.41, 410.50, 410.51, 410.60, 410.61, 410.70, 410.71, 410.80, 410.81, 410.90, 410.91 - **ICD-10:** I21, I22   - **Congestive heart failure**: 1 point (definition mentioned above: ‘Congestive heart failure’)  - **Peripheral vascular disease**: 1 point (definition mentioned above: ‘Peripheral artery disease’)  - **Cerebrovascular disease:** 1 point   - **ICD-9:** 362.34, 430, 431, 432, 433, 434, 435, 436, 437, 438 - **ICD-10:** I61, I62, I63, I65, I66, I67, I68, I69, G45, G46, H34.0   - **Dementia**: 1 point (definition mentioned above: ‘Dementia’)  - **Chronic pulmonary disease**: 1 point (definition mentioned above: ‘Chronic lung disease’)  - **Connective tissue disease**: 1 point:   - **ICD-9:** 136.1, 287.0, 446, 447.5, 447.6, 710, 711.2, 725, 728.5, 729.30 - **ICD-10:** D69.0, M30, M31, M32, M33, M34, M35, M36.0, M36.8   - **Peptic ulcer disease**: 1 point:   - **ICD-9:** 041.86, 530.2, 531, 532, 533, 534, V12.71 - **ICD-10:** B96.81, K22.1, K25, K26, K27, K28, Z87.11 - **ATC:** A02BD04, A02BD11 - **Medical procedure code:** 550093, 550104, 552370, 552381   - **Mild liver disease**: 1 point   - **ICD-9:** 070.3, 070.51, 070.52, 070.54, 070.70, 571, 572.8, 573.0, 573.8, 573.9 - **ICD-10:** B17.0, B17.10, B18, B19.10, B19.20, K70.0, K70.1, K70.2, K70.3, K70.9, K71.3, K71.4, K71.5, K71.6, K71.7, K71.8, K71.9, K73, K74, K75.3, K75.4, K75.8, K75.9, K76.0, K76.1, K76.2, K76.89, K76.9, K77 - **ATC:** J05AB04, J05AF05, J05AF07, J05AF08, J05AF10, J05AE11, J05AE12, J05AE14, J05AX15, J05AX65, J05AP - **Medical procedure code:** 556754, 556765   - **Diabetes without chronic complications**: 1 point   - **ICD-9:** 249.4-249.7, 249.9, 250.4-250.7, 250.9, 357.2, 362.0, 364.41 - **ICD-10:** E08.2-E08.5, E08.8, E09.2-E09.5, E09.8, E10.2-E10.5, E10.8, E11.2-E11.5, E11.8, E13.2-E13.5, E13.8 - **Medical procedure code:** 653671, 653682, 697093, 697104, 770070, 773393, 773496   - **Diabetes with chronic complications**: 2 points   - **ICD-9:** 249.0-249.3, 249.8, 250.0-250.3, 250.8 - **ICD-10:** E08.0, E08.1, E08.6, E08.9, E09.0, E09.1, E09.6, E09.9, E10.1, E10.6, E10.9, E11.0, E11.1, E11.6, E11.9, E13.0, E13.1, E13.6, E13.9   - **Hemiplegia or paraplegia**: 2 points:   - **ICD-9:** 334.1, 342, 343.0, 343.1, 343.2, 343.4, 344.0, 344.1, 344.2, 344.9, 438.2 - **ICD-10:** G04.1, G11.4, G80.0, G80.1, G80.2, G81, G82, G83.0, G86.9, I69.05, I69.15, I69.25, I69.35, I69.85, I69.95 - **Medical procedure code:** 643414, 643425   - **Renal disease:** 2 points   - **ICD-9:** 403.01, 403.11, 403.91, 404.02, 404.12, 404.92, 585.5, 585.6, 586, 996.81, V42.0, V45.11, V56 - **ICD-10:** N18.5, N18.6, N19, I12.0, I13.11, T86.1, Z49, Z94.0, Z99.2 - **Medical procedure code:** N81 (group code), 318010, 318021, 318290, 318301   - **Any malignancy, including leukemia and lymphoma:** 2 points:   - **ICD-9:** 140-195, 199.1, 199.2, 200-209, 223, 230-239, 258.0, V58.0, V58.11, V58.12 - **ICD-10:** C00-C76, C80.1, C80.2, C81-C96, D00-D09, D37-D49, E31.2, Z51.0, Z51.11, Z51.12 - **ATC:** L01 - **Medical procedure code:** 154873, 154884, 154895, 154906, 157231, 157242, 201191, 201202, 201213, 201224, 220275, 220286, 220371, 220382, 201213, 201224, 226914, 226925, 226936, 226940, 227216, 227220, 227275, 227286, 227636, 227640, 227651, 227662, 227673, 227684, 227695, 227706, 227710, 227721, 227732, 227743, 227754, 227765, 227776, 227780, 227791, 227802, 227813, 227824, 227835, 227846, 228012, 228023, 228174, 228185, 228233, 228244, 228255, 228266, 228270, 228281, 228292, 228303, 228314, 228325, 228336, 228340, 230473, 230484, 231033, 231044, 241231, 241242, 241415, 241426, 241430, 241441, 241452, 241463, 241555, 241566, 242012, 242023, 242034, 242045, 242292, 242303, 242314, 242325, 242830, 242841, 242852, 242863, 242874, 242885, 242896, 242900, 243051, 243062, 243073, 243084, 243235, 243246, 243736, 243740, 243751, 243762, 243773, 243784, 244016, 244020, 244031, 244042, 244075, 244086, 244790, 244801, 244856, 244860, 244893, 244904, 244915, 244926, 244930, 244941, 244952, 244963, 244974, 244985, 245512, 245523, 245534, 245545, 246050, 246061, 246072, 246083, 247111, 247122, 247133, 247144, 251753, 251764, 251775, 251786, 254892, 254903, 256115, 256126, 256336, 256340, 256572, 256583, 257191, 257202, 258355, 258366, 258370, 258381, 258392, 258403, 258451, 258462, 258554, 258565, 258856, 258860, 258871, 258882, 258893, 258904, 259033, 259044, 259114, 259125, 260190, 260201, 260411, 260422, 260433, 260444, 260551, 260562, 260654, 260665, 260750, 260761, 261111, 261122, 261391, 261402, 261472, 261483, 261671, 261682, 261774, 261785, 261796, 261800, 262334, 262345, 262570, 262581, 277756, 277760, 277771, 277782, 278795, 278806, 278810, 278821, 281831, 281842, 281956, 281960, 282310, 282321, 282671, 282682, 284056, 284060, 288455, 288466, 288470, 288481, 289892, 289903, 291056, 291060, 310494, 310505, 311312, 311323, 312550, 312561, 312572, 312583, 312594, 312605, 312653, 312664, 312970, 312981, 350114, 350125, 350136, 350140, 350276, 350280, 350291, 350302, 350372, 350383, 350674, 350685, 350696, 350700, 431174, 431185, 431336, 431340, 431351, 431362, 432294, 432305, 444113, 444124, 444135, 444146, 444150, 444161, 444172, 444183, 444194, 444205, 444216, 444220, 444231, 444242, 444253, 444264, 444275, 444286, 444290, 444301, 444312, 444323, 444334, 444345, 444474, 444485, 444592, 444603, 473970, 473981, 474795, 474806, 565073, 565084, 565095, 565106, 565110, 565121, 565132, 565143, 565154, 565165, 587834, 587845, 587871, 587882, 587893, 587904, 587915, 587926, 588431, 588442, 588453, 588464, 588475, 588486, 588490, 588501, 588512, 588523, 588534, 588545, 588556, 588560, 588571, 588582, 588593, 588604, 588770, 588781, 588976, 588980, 589691, 589702, 589713, 589724, 589831, 589842, 589875, 589886, 594016, 594020, 594031, 594042, 594053, 594064, 594075, 594086, 594090, 594101, 594112, 594123, 594252, 594263, 594274, 594285, 594296, 594300, 594311, 594322, 594333, 594344, 594355, 594366, 594370, 594381, 594392, 594403, 594414, 594425, 594436, 594440, 594451, 594462, 594495, 594506, 594510, 594521, 594532, 594543, 594554, 594565, 594576, 594580, 594591, 594602, 594613, 594624, 594635, 594646, 594694, 594705, 594716, 594720, 594753, 594764, 594775, 594786, 594790, 594801, 594812, 594823, 594834, 594845, 594856, 594860, 594871, 594882, 594893, 594904, 594915, 594926, 594930, 594941, 598581, 682636, 682640, 682732, 682743, 687934, 687945, 698051, 698062, 698095, 698106, 698390, 698401, 698456, 698460, 698471, 698482, 698493, 698504, 698530, 698541, 745010, 745021, 745032, 745043, 745113, 745124, 745135, 745146, 745150, 745161   - **Moderate or severe liver disease:** 3 points   - **ICD-9:** 070.0, 070.2, 070.4, 070.6, 070.71, 155.0, 155.1, 155.2, 197.7, 456.0, 456.1, 456.2, 567.23, 570, 571.2, 571.5, 571.6, 572.2, 572.3, 572.4, 573.0, 573.5, 789.59, V42.7 - **ICD-10:** B15.0, B16.0, B16.2, B17.11, B19.0, B19.11, B19.21, C22, C78.7, I85, I86.4, K65.2, K70.2, K70.3, K70.4, K71.1, K71.7, K72, K74, K76.1, K76.5, K76.6, K76.7, K76.81, R18.8, Z94.4 - **Medical procedure code:** 318076, 318080, 318334, 318345, 472113, 472124, 589352, 589363   - **Metastatic solid tumor:** 6 points   - **ICD-9:** 196-198, 199.0 - **ICD-10:** C77-C79, C80.0   - **AIDS/HIV:** 6 points   - **ICD-9:** 042, V08 - **ICD-10:** B20, Z21 - **ATC:** J05AE01, J05AE02, J05AE03, J05AE04, J05AE05, J05AE07, J05AE08, J05AE09, J05AE10, J05AF01, J05AF02, J05AF03, J05AF04, J05AF05, J05AF06, J05AF09, J05AF11, J05AF12, J05AF13, J05AG, J05AR, J05AX07, J05AX08, J05AX09, J05AX12   - **Age:**   - <50 years: 0 points - 50-59 years: 1 point - 60-69 years: 2 points - 70-79 years: 3 points - ≥80 years: 4 points   *The following comorbid conditions were mutually exclusive: diabetes with chronic complications and diabetes without chronic complications; mild liver disease and moderate or severe liver disease; and any malignancy and metastatic solid tumor*. |
| **LABORATORY TESTING** | | |
| INR measurement | | **Medical procedure code:** 554573, 554584 |

**eTable 2:** Definition of in- and exclusion criteria, comorbidities, medication history and clinical risk scores based on ICD-coded hospital discharge diagnoses (ICD-9-CM up to 2014 and ICD-10-BE from 2015 onward)^2^, medical procedure codes^3^ and/or ATC-coded prescription claims^4^.

AAD: antiarrhythmic drug; AF: Atrial fibrillation; ARNI: angiotensin receptor-neprilysin inhibitor; ATC: Anatomical Therapeutic Chemical Classification; CKD: Chronic kidney disease; ICD-9-CM: International Classification of Diseases (ICD) codes, 9^th^ revision, Clinical Modification; ICD-10-BE: International Classification of Diseases (ICD) codes, 10^th^ Revision, Belgian Modification; INR: International Normalized Ratio; NOAC: non-vitamin K antagonist oral anticoagulant; NSAID: non-steroidal anti-inflammatory drug; OAC: oral anticoagulant; VKA: vitamin K antagonist; y: year.

## eTable 3: Definition of outcomes

| **OUTCOME** | **ICD, ATC AND MEDICAL PROCEDURE CODES** |
| --- | --- |
| Stroke or systemic embolism | **ICD 9:** 431, 432.9, 433.01, 433.1, 433.21, 433.31, 433.81, 433.91, 434.01, 434.11, 434.91, 436, 444, 557.0, 593.81  **ICD 10:** D73.5, I61, I62.9, I63.0, I63.1, I63.2, I63.3, I63.4, I63.5, I63.8, I63.9, I67.89, I74, K55.01, K55.02, N28.0  **Medical procedure code:** 182136, 182140, 182151, 182162, 182173, 182184, 235130, 235141, 237112, 237123, 477724, 477746, 477761, 477783, 589175, 589186 |
| Ischemic stroke | **ICD 9:** 433.01, 433.1, 433.21, 433.31, 433.81, 433.91, 434.01, 434.11, 434.91  **ICD 10:** I63.0, I63.1, I63.2, I63.3, I63.4, I63.5, I63.8, I63.9  **Medical procedure code:** 182136, 182140, 182151, 182162, 182173, 182184 |
| All-cause mortality | Death of date, based on the year and month of death, not the exact date due to patient privacy |
| Major bleeding | **ICD 9:** 336.1, 376.32, 372.72, 364.41, 363.6, 363.72, 362.81, 362.43, 379.23, 360.43, 377.42, 423.0, 430, 431, 432.0, 432.1, 432.9, 455.8, 456.0, 456.20, 530.21, 530.7, 530.82, 531.00, 531.20, 531.40, 531.60, 532.00, 532.20, 532.40, 532.60, 533.00, 533.20, 533.40, 533.60, 534.00, 534.20, 534.40, 534.60, 535.01, 535.11, 535.21, 535.31, 535.41, 535.51, 535.61, 535.71, 537.83, 537.84, 562.02, 562.03, 562.12, 562.13, 568.81, 569.3, 569.85, 569.86, 578.0, 578.1, 578.9, 719.1, 852.0, 852.2, 852.4, 853.0  **ICD 10:** H05.23, H11.3, H21.0, H31.3, H31.41, H35.6, H35.73, H43.1, H44.81, H47.02, I23.0, I31.2, I60, I61, I62.0, I62.1, I62.9, I85.01, I85.11, J94.2, K22.11, K22.6, K22.8, K25.0, K25.2, K25.4, K25.6, K26.0, K26.2, K26.4, K26.6, K27.0, K27.2, K27.4, K27.6, K28.0, K28.2, K28.4, K28.6, K29.01, K29.21, K29.31, K29.41, K29.51, K29.61, K29.71, K29.81, K29.91, K31.811, K31.82, K50.011, K50.111, K50.811, K50.911, K51.011, K51.211, K51.311, K51.411, K51.511, K51.811, K51.911, K55.21, K57.01, K57.11, K57.13, K57.21, K57.31, K57.33, K57.41, K57.51, K57.53, K57.81, K57.91, K57.93, K62.5, K63.81, K64.9, K66.1, K92.0, K92.1, K92.2, K94.01, K94.11, K94.21, K94.31, G95.19, M25.0, S06.340A, S06.341A, S06.342A, S06.343A, S06.344A, S06.345A, S06.346A, S06.347A, S06.348A, S06.349A, S06.350A, S06.351A, S06.352A, S06.353A, S06.354A, S06.355A, S06.356A, S06.357A, S06.358A, S06.359A, S06.360A, S06.361A, S06.362A, S06.363A, S06.364A, S06.365A, S06.366A, S06.367A, S06.368A, S06.369A, S06.4X0A, S06.4X1A, S06.4X2A, S06.4X3A, S06.4X4A, S06.4X5A, S06.4X6A, S06.4X7A, S06.4X8A, S06.4X9A, S06.5X0A, S06.5X1A, S06.5X2A, S06.5X3A, S06.5X4A, S06.5X5A, S06.5X6A, S06.5X7A, S06.5X8A, S06.5X9A, S06.6X0A, S06.6X1A, S06.6X2A, S06.6X3A, S06.6X4A, S06.6X5A, S06.6X6A, S06.6X7A, S06.6X8A, S06.6X9A  **Medical procedure code (hospitalized):** 227441, 230403, 230425, 230440, 243600, 431620, 472124, 473686, 473782  ***OR*** Clinically relevant non-major bleeding with blood transfusion or death within 10 days after admission^9,10^: **Medical procedure code (blood transfusion):** 752124, 752463, 752581 |
| Intracranial bleeding | **ICD 9:** 430, 431, 432.0, 432.1, 432.9, 852.0, 852.2, 852.4, 853.0  **ICD 10:** I60, I61, I62.0, I62.1, I62.9, S06.340A, S06.341A, S06.342A, S06.343A, S06.344A, S06.345A, S06.346A, S06.347A, S06.348A, S06.349A, S06.350A, S06.351A, S06.352A, S06.353A, S06.354A, S06.355A, S06.356A, S06.357A, S06.358A, S06.359A, S06.360A, S06.361A, S06.362A, S06.363A, S06.364A, S06.365A, S06.366A, S06.367A, S06.368A, S06.369A, S06.4X0A, S06.4X1A, S06.4X2A, S06.4X3A, S06.4X4A, S06.4X5A, S06.4X6A, S06.4X7A, S06.4X8A, S06.4X9A, S06.5X0A, S06.5X1A, S06.5X2A, S06.5X3A, S06.5X4A, S06.5X5A, S06.5X6A, S06.5X7A, S06.5X8A, S06.5X9A, S06.6X0A, S06.6X1A, S06.6X2A, S06.6X3A, S06.6X4A, S06.6X5A, S06.6X6A, S06.6X7A, S06.6X8A, S06.6X9A  **Medical procedure (hospitalized):** 230403, 230425, 230440 |
| Gastrointestinal bleeding | **ICD 9:** 455.8, 456.0, 456.20, 530.21, 530.7, 530.82, 531.00, 531.20, 531.40, 531.60, 532.00, 532.20, 532.40, 532.60, 533.00, 533.20, 533.40, 533.60, 534.00, 534.20, 534.40, 534.60, 535.01, 535.11, 535.21, 535.31, 535.41, 535.51, 535.61, 535.71, 537.83, 537.84, 562.02, 562.03, 562.12, 562.13, 569.3, 569.85, 569.86, 578.0, 578.1, 578.9  **ICD 10:** I85.01, I85.11, K22.11, K22.6, K22.8, K25.0, K25.2, K25.4, K25.6, K26.0, K26.2, K26.4, K26.6, K27.0, K27.2, K27.4, K27.6, K28.0, K28.2, K28.4, K28.6, K29.01, K29.21, K29.31, K29.41, K29.51, K29.61, K29.71, K29.81, K29.91, K31.811, K31.82, K50.011, K50.111, K50.811, K50.911, K51.011, K51.211, K51.311, K51.411, K51.511, K51.811, K51.911, K55.21, K57.01, K57.11, K57.13, K57.21, K57.31, K57.33, K57.41, K57.51, K57.53, K57.81, K57.91, K57.93, K62.5, K63.81, K64.9, K92.0, K92.1, K92.2, K94.01, K94.11, K94.21, K94.31  **Medical procedure (hospitalized):** 472124, 473686, 473782 |
| Fall | **ICD-9:** E804, E833, E834, E835, E843, E880-E886, E888, E917.5-E917.9, E987  **ICD-10:** V00.141, V00.811, V00.831, V81.5, V81.6, V82.5, V82.6, V92.0, V93.3, V94.0, V97.0, W00, W01, W03, W05-W15, W16.0-W16.4, W17, W18, W19, Y21.1, Y21.3, Y30 |

**eTable 3:** Definition of outcomes based on ICD-coded hospital discharge diagnoses (ICD-9-CM up to 2014 and ICD-10-BE from 2015 onward)^2^, medical procedure codes^3^ and/or ATC-coded prescription claims^4^. ATC: Anatomical Therapeutic Chemical Classification; ICD-9-CM: International Classification of Diseases (ICD) codes, 9^th^ revision, Clinical Modification; ICD-10-BE: International Classification of Diseases (ICD) codes, 10^th^ Revision, Belgian Modification.

eTable 4: Baseline characteristics of NOAC users with history of falls

| **Patient characteristics** | | **Dabigatran**  **(n = 1705)** | **Rivaroxaban**  **(n = 4896)** | **Apixaban**  **(n = 6536)** | **Edoxaban**  **(n = 2134)** |
| --- | --- | --- | --- | --- | --- |
|  | Age (years) | 80.0 ± 9.1 | 81.0 ± 9.2 | 82.3 ± 8.7 | 81.5 ± 9.3 |
|  | Female | 994 (58.3%) | 2982 (60.9%) | 3946 (60.4%) | 1213 (56.8%) |
|  | Reduced dose NOAC | 1201 (70.4%) | 2643 (54.0%) | 3178 (48.6%) | 1098 (51.5%) |
|  | Follow-up (years) | 1.1 ± 1.3 | 1.1 ± 1.3 | 0.9 ± 1.0 | 0.6 ± 0.5 |
| **Comorbidities** | | | | | |
|  | Hypertension | 1376 (80.7%) | 3920 (80.1%) | 5361 (82.0%) | 1706 (79.9%) |
|  | Coronary artery disease | 483 (28.3%) | 1368 (27.9%) | 1926 (29.5%) | 604 (28.3%) |
|  | Congestive heart failure | 474 (27.8%) | 1537 (31.4%) | 2204 (33.7%) | 696 (32.6%) |
|  | Valvular heart disease | 433 (25.4%) | 1340 (27.4%) | 1883 (28.8%) | 603 (28.3%) |
|  | Peripheral artery disease | 254 (14.9%) | 756 (15.4%) | 1097 (16.8%) | 293 (13.7%) |
|  | Dyslipidemia | 1029 (60.4%) | 2655 (54.2%) | 3779 (57.8%) | 1235 (57.9%) |
|  | Chronic kidney disease | 355 (20.8%) | 1364 (27.9%) | 2177 (33.3%) | 720 (33.7%) |
|  | Chronic liver disease | 150 (8.8%) | 515 (10.5%) | 575 (8.8%) | 190 (8.9%) |
|  | Chronic lung disease | 403 (23.7%) | 1279 (26.1%) | 1637 (25.0%) | 502 (23.5%) |
|  | Obstructive sleep apnea | 64 (3.7%) | 229 (4.7%) | 247 (3.8%) | 68 (3.2%) |
|  | Cancer | 261 (15.3%) | 897 (18.3%) | 1176 (18.0%) | 454 (21.3%) |
|  | Upper GI tract disorder^**^ | 325 (19.1%) | 1006 (20.5%) | 1265 (19.4%) | 320 (15.0%) |
|  | Lower GI tract disorder^**^ | 216 (12.6%) | 610 (12.5%) | 795 (12.2%) | 262 (12.3%) |
|  | Diabetes mellitus | 1311 (76.9%) | 3855 (78.7%) | 4743 (72.6%) | 1499 (70.2%) |
|  | Anemia | 418 (24.5%) | 1389 (28.4%) | 1916 (29.3%) | 608 (28.5%) |
|  | Thyroid disease | 366 (21.5%) | 1112 (22.7%) | 1469 (22.5%) | 374 (17.5%) |
|  | Depression | 667 (39.1%) | 2111 (43.1%) | 2603 (39.8%) | 723 (33.9%) |
|  | Dementia | 341 (20.0%) | 1171 (23.9%) | 1460 (22.3%) | 400 (18.7%) |
|  | Parkinson’s disease | 131 (7.7%) | 339 (6.9%) | 517 (7.9%) | 137 (6.4%) |
|  | Frailty | 1151 (67.5%) | 3562 (72.7%) | 4970 (76.0%) | 1501 (70.3%) |
|  | Prior stroke/SE | 648 (38.0%) | 1380 (28.2%) | 2075 (31.8%) | 472 (22.1%) |
|  | Prior MB/CRNMB | 316 (18.5%) | 853 (17.4%) | 1184 (18.1%) | 352 (16.5%) |
| **Medication history** | | | | | |
|  | Number of concomitant drugs | 8.9 ± 5.1 | 9.6 ± 5.3 | 9.8 ± 5.2 | 9.7 ± 5.1 |
|  | Beta blockers | 1165 (68.3%) | 3227 (65.9%) | 4573 (70.0%) | 1516 (71.0%) |
|  | Verapamil, diltiazem | 66 (3.9%) | 219 (4.5%) | 264 (4.0%) | 67 (3.1%) |
|  | Digoxin | 248 (14.5%) | 777 (15.9%) | 1072 (16.4%) | 380 (17.8%) |
|  | Class I AAD | 100 (5.9%) | 367 (7.5%) | 346 (5.3%) | 140 (6.6%) |
|  | Class III AAD | 478 (28.0%) | 1456 (29.7%) | 1865 (28.5%) | 580 (27.2%) |
|  | Acetylsalicylic acid | 675 (39.6%) | 1958 (40.0%) | 2746 (42.0%) | 880 (41.2%) |
|  | P2Y12 inhibitor | 101 (5.9%) | 292 (6.0%) | 517 (7.9%) | 196 (9.2%) |
|  | Proton pump inhibitor | 906 (53.1%) | 2748 (56.1%) | 3729 (57.1%) | 1238 (58.0%) |
|  | NSAID | 422 (24.8%) | 1195 (24.4%) | 1456 (22.3%) | 485 (22.7%) |
|  | Oral corticosteroids | 462 (27.1%) | 1418 (29.0%) | 1879 (28.7%) | 642 (30.1%) |
|  | SSRI/SNRI | 377 (22.1%) | 1095 (22.4%) | 1362 (20.8%) | 370 (17.3%) |
| **Clinical risk score** | | | | | |
|  | CHA_2_DS_2_-VASc score | 5.2 ± 1.6 | 5.1 ± 1.7 | 5.3 ± 1.6 | 4.9 ± 1.6 |
|  | HAS-BLED score | 3.5 ± 1.3 | 3.5 ± 1.4 | 3.6 ± 1.3 | 3.4 ± 1.3 |
|  | Charlson Comorbidity Index | 6.6 ± 2.6 | 6.7 ± 2.9 | 6.7 ± 2.6 | 6.5 ± 2.4 |

**eTable 4:** Baseline characteristics of NOAC-treated AF patients with a history of falls.

Data shown as mean ± standard deviation, or counts and percentages. ^*^Upper and lower gastrointestinal tract disorders were defined as gastroesophageal reflux disease or peptic ulcer disease; and diverticulosis, angiodysplasia, colorectal polyposis or hemorrhoids, respectively. AAD: antiarrhythmic drug; AF: atrial fibrillation; CRNMB: clinically relevant non-major bleeding; GI: gastrointestinal; MB: major bleeding; NA: not applicable; NOAC: non-vitamin K antagonist oral anticoagulant; NSAID: non-steroidal anti-inflammatory drug; OAC: oral anticoagulant; SE: systemic embolism; SMD: standardized mean difference; SNRI: serotonin and norepinephrine reuptake inhibitor; SSRI: selective serotonin reuptake inhibitor.

## eTable 5: Number of events and crude event rates (main analysis)

| **Outcome** | **No previous falls**  **(n = 235,531)** | **History of falls** | | | | | | |
| --- | --- | --- | --- | --- | --- | --- | --- | --- |
|  |  | **Overall history of falls (n = 18,947)** | **VKA**  **(n = 3676)** | **NOAC**  **(n = 15,271)** | **Dabigatran**  **(n = 1705)** | **Rivaroxaban**  **(n = 4896)** | **Apixaban**  **(n = 6536)** | **Edoxaban**  **(n = 2134)** |
|  | **Events (per 100 PY)** | **Events (per 100 PY)** | **Events (per 100 PY)** | **Events (per 100 PY)** | **Events (per 100 PY)** | **Events (per 100 PY)** | **Events (per 100 PY)** | **Events (per 100 PY)** |
| **Effectiveness** |  |  |  |  |  |  |  |  |
| Stroke/SE | 6694 (2.18) | 686 (4.26) | 136 (5.88) | 550 (3.99) | 72 (4.02) | 196 (3.89) | 239 (4.13) | 43 (3.60) |
| Ischemic stroke | 3495 (1.13) | 400 (2.45) | 84 (3.58) | 316 (2.26) | 45 (2.49) | 119 (2.34) | 132 (2.25) | 20 (1.66) |
| All-cause mortality | 21154 (6.78) | 3699 (22.30) | 574 (23.98) | 3125 (22.02) | 313 (16.96) | 1094 (21.13) | 1414 (23.73) | 304 (25.09) |
| **Safety** |  |  |  |  |  |  |  |  |
| Major bleeding | 13372 (4.44) | 1344 (8.51) | 229 (10.11) | 1115 (8.24) | 140 (8.06) | 428 (8.71) | 412 (7.22) | 135 (11.57) |
| Intracranial bleeding | 3465 (1.12) | 337 (2.06) | 56 (2.40) | 281 (2.01) | 33 (1.83) | 120 (2.36) | 97 (1.64) | 31 (2.58) |
| Gastrointestinal bleeding | 6944 (2.26) | 709 (4.37) | 105 (4.46) | 604 (4.36) | 90 (5.02) | 237 (4.70) | 204 (3.50) | 73 (6.15) |
| **Other** |  |  |  |  |  |  |  |  |
| New fall | 14436 (4.82) | 2346 (15.68) | 343 (15.99) | 2003 (15.63) | 231 (14.12) | 689 (14.71) | 855 (15.86) | 228 (20.58) |

**eTable 5:** The number of events and crude event rates per 100 person-years of outcomes.

NOAC: non-vitamin K antagonist oral anticoagulant; PY: person-year; SE: systemic embolism; VKA: vitamin K antagonist.

## eTable 6: NOAC versus VKA (main analysis)

|  | **NOAC vs VKA** | | **Dabigatran vs VKA** | | **Rivaroxaban vs VKA** | | **Apixaban vs VKA** | | **Edoxaban vs VKA** | |
| --- | --- | --- | --- | --- | --- | --- | --- | --- | --- | --- |
|  | **aHR^*^ (95%CI)** | **p-value** | **aHR^*^ (95%CI)** | **p-value** | **aHR^*^ (95%CI)** | **p-value** | **aHR^*^ (95%CI)** | **p-value** | **aHR^*^ (95%CI)** | **p-value** |
| **Effectiveness** |  |  |  |  |  |  |  |  |  |  |
| Stroke/SE | 0.70 (0.57-0.87) | <0.001 | 0.75 (0.55-1.02) | 0.071 | 0.79 (0.63-0.99) | 0.049 | 0.71 (0.55-0.91) | 0.008 | 0.60 (0.33-1.08) | 0.089 |
| Ischemic stroke | 0.59 (0.45-0.77) | <0.001 | 0.71 (0.47-1.05) | 0.085 | 0.76 (0.56-1.02) | 0.065 | 0.60 (0.42-0.84) | 0.003 | 0.39 (0.17-0.91) | 0.030 |
| All-cause mortality | 0.83 (0.75-0.92) | <0.001 | 0.81 (0.70-0.95) | 0.009 | 0.87 (0.78-0.97) | 0.012 | 0.86 (0.76-0.96) | 0.009 | 0.75 (0.60-0.95) | 0.016 |
| **Safety** |  |  |  |  |  |  |  |  |  |  |
| Major bleeding | 0.89 (0.76-1.05) | 0.158 | 0.93 (0.74-1.17) | 0.519 | 1.04 (0.87-1.23) | 0.670 | 0.77 (0.63-0.94) | 0.009 | 0.89 (0.63-1.27) | 0.536 |
| Intracranial bleeding | 0.86 (0.62-1.19) | 0.376 | 0.91 (0.57-1.45) | 0.691 | 1.14 (0.81-1.61) | 0.448 | 0.72 (0.48-1.09) | 0.124 | 0.93 (0.45-1.95) | 0.856 |
| Gastrointestinal bleeding | 1.03 (0.81-1.30) | 0.809 | 1.28 (0.94-1.74) | 0.114 | 1.22 (0.95-1.56) | 0.115 | 0.78 (0.58-1.03) | 0.080 | 1.03 (0.61-1.74) | 0.914 |

**eTable 6:** Adjusted hazard ratios with 95% confidence intervals of outcomes compared between (individual) NOACs and VKAs in AF patients with a history of falls after IPTW.

^*^ Propensity scores used for stabilized IPTW were calculated with logistic regression models including 39 confounding covariates (age, sex, baseline comorbidities, medication history, clinical risk scores), stratified by calendar year.

aHR: adjusted hazard ratio; CI: confidence interval; IPTW: inverse probability of treatment weighting; NOAC: non-vitamin K antagonist oral anticoagulant; SE: systemic embolism; VKA: vitamin K antagonist; vs: versus.

eTable 7: NOAC versus NOAC (main analysis)

|  | **Dabigatran vs Rivaroxaban** | | **Apixaban vs Rivaroxaban** | | **Edoxaban vs Rivaroxaban** | | **Apixaban vs Dabigatran** | | **Dabigatran vs Edoxaban** | | **Apixaban vs Edoxaban** | |
| --- | --- | --- | --- | --- | --- | --- | --- | --- | --- | --- | --- | --- |
|  | **aHR^*^ (95%CI)** | **p-value** | **aHR^*^ (95%CI)** | **p-value** | **aHR^*^ (95%CI)** | **p-value** | **aHR^*^ (95%CI)** | **p-value** | **aHR^*^ (95%CI)** | **p-value** | **aHR^*^ (95%CI)** | **p-value** |
| **Effectiveness** |  |  |  |  |  |  |  |  |  |  |  |  |
| Stroke/SE | 0.96 (0.72-1.28) | 0.795 | 0.99 (0.80-1.22) | 0.904 | 0.68 (0.45-1.03) | 0.067 | 1.08 (0.78-1.49) | 0.633 | 0.89 (0.47-1.67) | 0.722 | 1.29 (0.90-1.85) | 0.172 |
| Ischemic stroke | 0.93 (0.65-1.33) | 0.676 | 0.92 (0.70-1.22) | 0.566 | 0.68 (0.37-1.24) | 0.206 | 1.03 (0.68-1.55) | 0.892 | 1.47 (0.67-3.26) | 0.340 | 1.56 (0.92-2.64) | 0.099 |
| All-cause mortality | 0.86 (0.76-0.98) | 0.028 | 0.97 (0.89-1.05) | 0.456 | 0.80 (0.68-0.94) | 0.007 | 1.25 (1.07-1.45) | 0.004 | 0.79 (0.59-1.04) | 0.098 | 1.24 (1.08-1.42) | 0.002 |
| **Safety** |  |  |  |  |  |  |  |  |  |  |  |  |
| Major bleeding | 0.97 (0.80-1.19) | 0.799 | 0.78 (0.68-0.91) | <0.001 | 0.98 (0.76-1.27) | 0.900 | 0.78 (0.62-0.98) | 0.035 | 1.09 (0.75-1.59) | 0.648 | 0.74 (0.59-0.92) | 0.008 |
| Intracranial bleeding | 0.81 (0.54-1.22) | 0.318 | 0.72 (0.54-0.96) | 0.026 | 1.03 (0.61-1.74) | 0.905 | 0.86 (0.53-1.39) | 0.545 | 1.22 (0.54-2.75) | 0.640 | 0.80 (0.50-1.26) | 0.330 |
| Gastrointestinal bleeding | 1.12 (0.87-1.45) | 0.380 | 0.68 (0.56-0.84) | <0.001 | 0.93 (0.66-1.31) | 0.677 | 0.56 (0.42-0.75) | <0.001 | 1.55 (0.98-2.45) | 0.060 | 0.67 (0.50-0.91) | 0.011 |

**eTable 7:** Adjusted hazard ratios with 95% confidence intervals of outcomes compared between individual NOAC types in AF patients with a history of falls after IPTW.

^*^ Propensity scores used for stabilized IPTW were calculated with logistic regression models including 39 confounding covariates (age, sex, baseline comorbidities, medication history, clinical risk scores), stratified by calendar year.

aHR: adjusted hazard ratio; CI: confidence interval; IPTW: inverse probability of treatment weighting; NOAC: non-vitamin K antagonist oral anticoagulant; SE: systemic embolism; vs: versus.

eTable 8: Intention-to-treat analysis (sensitivity analysis)

**A)**

| **Outcome** | **No previous falls**  **(n = 235,531)** | **History of falls** | | | | | | |
| --- | --- | --- | --- | --- | --- | --- | --- | --- |
|  |  | **Overall history of falls (n = 18,947)** | **VKA**  **(n = 3676)** | **NOAC**  **(n = 15,271)** | **Dabigatran**  **(n = 1705)** | **Rivaroxaban**  **(n = 4896)** | **Apixaban**  **(n = 6536)** | **Edoxaban**  **(n = 2134)** |
|  | **Events (per 100 PY)** | **Events (per 100 PY)** | **Events (per 100 PY)** | **Events (per 100 PY)** | **Events (per 100 PY)** | **Events (per 100 PY)** | **Events (per 100 PY)** | **Events (per 100 PY)** |
| **Effectiveness** |  |  |  |  |  |  |  |  |
| Stroke/SE | 12685 (2.09) | 1257 (3.96) | 337 (4.48) | 920 (3.79) | 142 (4.08) | 353 (3.66) | 363 (3.83) | 62 (3.76) |
| Ischemic stroke | 6866 (1.11) | 749 (2.32) | 190 (2.47) | 559 (2.27) | 94 (2.66) | 223 (2.28) | 210 (2.18) | 32 (1.92) |
| All-cause mortality | 46111 (7.36) | 7237 (21.76) | 1691 (21.17) | 5546 (21.94) | 678 (18.54) | 2209 (21.88) | 2246 (22.83) | 413 (24.57) |
| **Safety** |  |  |  |  |  |  |  |  |
| Major bleeding | 23857 (4.05) | 2325 (7.64) | 553 (7.70) | 1772 (7.62) | 244 (7.32) | 723 (7.90) | 627 (6.81) | 178 (11.29) |
| Intracranial bleeding | 6124 (1.00) | 578 (1.78) | 158 (2.04) | 420 (1.70) | 55 (1.55) | 178 (1.81) | 148 (1.53) | 39 (2.35) |
| Gastrointestinal bleeding | 12684 (2.09) | 1250 (3.93) | 273 (3.58) | 977 (4.04) | 152 (4.36) | 402 (4.20) | 322 (3.38) | 101 (6.23) |
| **Other** |  |  |  |  |  |  |  |  |
| New fall | 27570 (4.68) | 4004 (14.08) | 866 (12.86) | 3138 (14.46) | 393 (12.70) | 1144 (13.32) | 1311 (15.40) | 290 (19.18) |

**B)**

|  | **NOAC vs VKA** | | **Dabigatran vs VKA** | | **Rivaroxaban vs VKA** | | **Apixaban vs VKA** | | **Edoxaban vs VKA** | |
| --- | --- | --- | --- | --- | --- | --- | --- | --- | --- | --- |
|  | **aHR^*^ (95%CI)** | **p-value** | **aHR^*^ (95%CI)** | **p-value** | **aHR^*^ (95%CI)** | **p-value** | **aHR^*^ (95%CI)** | **p-value** | **aHR^*^ (95%CI)** | **p-value** |
| **Effectiveness** |  |  |  |  |  |  |  |  |  |  |
| Stroke/SE | 0.80 (0.70-0.92) | 0.002 | 0.91 (0.73-1.13) | 0.379 | 0.85 (0.73-0.99) | 0.045 | 0.76 (0.64-0.91) | 0.003 | 0.73 (0.47-1.15) | 0.178 |
| Ischemic stroke | 0.78 (0.65-0.94) | 0.009 | 0.98 (0.74-1.29) | 0.886 | 0.93 (0.76-1.14) | 0.470 | 0.71 (0.56-0.90) | 0.005 | 0.57 (0.30-1.06) | 0.077 |
| All-cause mortality | 0.90 (0.85-0.96) | <0.001 | 0.90 (0.82-0.99) | 0.041 | 0.95 (0.89-1.02) | 0.149 | 0.89 (0.83-0.96) | 0.004 | 0.86 (0.72-1.03) | 0.094 |
| **Safety** |  |  |  |  |  |  |  |  |  |  |
| Major bleeding | 0.97 (0.87-1.08) | 0.569 | 1.05 (0.88-1.24) | 0.610 | 1.09 (0.96-1.22) | 0.170 | 0.84 (0.73-0.97) | 0.015 | 0.92 (0.70-1.21) | 0.559 |
| Intracranial bleeding | 0.78 (0.64-0.96) | 0.017 | 0.82 (0.58-1.16) | 0.271 | 0.93 (0.74-1.16) | 0.512 | 0.73 (0.56-0.96) | 0.024 | 0.94 (0.54-1.66) | 0.839 |
| Gastrointestinal bleeding | 1.10 (0.94-1.27) | 0.236 | 1.31 (1.05-1.63) | 0.019 | 1.23 (1.04-1.45) | 0.014 | 0.84 (0.69-1.02) | 0.080 | 0.98 (0.66-1.45) | 0.927 |

**C)**

|  | **Dabigatran vs Rivaroxaban** | | **Apixaban vs Rivaroxaban** | | **Edoxaban vs Rivaroxaban** | | **Apixaban vs Dabigatran** | | **Dabigatran vs Edoxaban** | | **Apixaban vs Edoxaban** | |
| --- | --- | --- | --- | --- | --- | --- | --- | --- | --- | --- | --- | --- |
|  | **aHR^*^ (95%CI)** | **p-value** | **aHR^*^ (95%CI)** | **p-value** | **aHR^*^ (95%CI)** | **p-value** | **aHR^*^ (95%CI)** | **p-value** | **aHR^*^ (95%CI)** | **p-value** | **aHR^*^ (95%CI)** | **p-value** |
| **Effectiveness** |  |  |  |  |  |  |  |  |  |  |  |  |
| Stroke/SE | 1.01 (0.83-1.25) | 0.892 | 0.94 (0.80-1.11) | 0.468 | 0.80 (0.56-1.14) | 0.216 | 0.96 (0.76-1.22) | 0.756 | 0.71 (0.42-1.19) | 0.196 | 1.17 (0.86-1.59) | 0.312 |
| Ischemic stroke | 1.01 (0.79-1.31) | 0.921 | 0.87 (0.70-1.07) | 0.183 | 0.85 (0.51-1.40) | 0.515 | 0.86 (0.64-1.16) | 0.334 | 1.03 (0.54-1.95) | 0.935 | 1.26 (0.82-1.92) | 0.286 |
| All-cause mortality | 0.90 (0.82-0.98) | 0.015 | 0.94 (0.88-1.00) | 0.062 | 0.87 (0.75-0.99) | 0.048 | 1.10 (0.99-1.22) | 0.064 | 0.87 (0.70-1.08) | 0.194 | 1.12 (1.00-1.26) | 0.051 |
| **Safety** |  |  |  |  |  |  |  |  |  |  |  |  |
| Major bleeding | 0.98 (0.84-1.14) | 0.811 | 0.79 (0.70-0.88) | <0.001 | 0.96 (0.77-1.20) | 0.735 | 0.83 (0.70-1.00) | 0.051 | 1.13 (0.83-1.54) | 0.446 | 0.73 (0.60-0.89) | <0.001 |
| Intracranial bleeding | 0.88 (0.64-1.21) | 0.424 | 0.81 (0.64-1.03) | 0.088 | 1.14 (0.71-1.82) | 0.585 | 0.91 (0.62-1.34) | 0.634 | 1.25 (0.63-2.48) | 0.519 | 0.87 (0.59-1.30) | 0.503 |
| Gastrointestinal bleeding | 1.09 (0.90-1.33) | 0.368 | 0.71 (0.60-0.83) | <0.001 | 0.97 (0.73-1.30) | 0.845 | 0.69 (0.54-0.87) | 0.002 | 1.36 (0.93-2.01) | 0.115 | 0.63 (0.49-0.82) | <0.001 |

**eTable 8:** The **A)** number of events and crude event rates per 100 person-years of outcomes in AF patients with and without a history of falls, and adjusted hazard ratios with 95% confidence intervals of outcomes compared between **B)** (individual) NOACs and VKAs, and **C)** between individual NOAC types in AF patients with a history of falls after IPTW using an intention-to-treatment analysis. In this approach, the end of follow-up was defined as the first occurrence of an outcome, death, emigration or end of study period, whichever occurred first.

^*^ Propensity scores used for stabilized IPTW were calculated with logistic regression models including 39 confounding covariates (age, sex, baseline comorbidities, medication history, clinical risk scores), stratified by calendar year. aHR: adjusted hazard ratio; CI: confidence interval; IPTW: inverse probability of treatment weighting; MB: major bleeding; NOAC: non-vitamin K antagonist oral anticoagulant; PY: person-year; SE: systemic embolism; VKA: vitamin K antagonist; vs: versus.

eTable 9: ICD-coded hospital discharge diagnosis of AF (sensitivity analysis)

**A)**

| **Outcome** | **No previous falls**  **(n = 110,329)** | **History of falls** | | | | | | |
| --- | --- | --- | --- | --- | --- | --- | --- | --- |
|  |  | **Overall history of falls**  **(n = 14,980)** | **VKA**  **(n = 2278)** | **NOAC**  **(n = 12,702)** | **Dabigatran**  **(n = 1387)** | **Rivaroxaban**  **(n = 3948)** | **Apixaban**  **(n = 5621)** | **Edoxaban**  **(n = 1746)** |
|  | **Events (per 100 PY)** | **Events (per 100 PY)** | **Events (per 100 PY)** | **Events (per 100 PY)** | **Events (per 100 PY)** | **Events (per 100 PY)** | **Events (per 100 PY)** | **Events (per 100 PY)** |
| **Effectiveness** |  |  |  |  |  |  |  |  |
| Stroke/SE | 3948 (2.80) | 580 (4.39) | 98 (6.02) | 482 (4.16) | 63 (4.29) | 158 (3.85) | 223 (4.42) | 38 (3.99) |
| Ischemic stroke | 2216 (1.56) | 347 (2.60) | 68 (4.13) | 279 (2.38) | 39 (2.62) | 101 (2.43) | 121 (2.36) | 18 (1.88) |
| All-cause mortality | 12843 (8.90) | 3168 (23.27) | 428 (25.39) | 2740 (22.97) | 266 (17.46) | 919 (21.75) | 1283 (24.62) | 272 (28.15) |
| **Safety** |  |  |  |  |  |  |  |  |
| Major bleeding | 8049 (5.85) | 1177 (9.11) | 179 (11.23) | 998 (8.81) | 132 (9.28) | 366 (9.15) | 378 (7.59) | 122 (13.19) |
| Intracranial bleeding | 2132 (1.50) | 289 (2.16) | 41 (2.49) | 248 (2.11) | 32 (2.16) | 100 (2.41) | 89 (1.73) | 27 (2.82) |
| Gastrointestinal bleeding | 4081 (2.89) | 623 (4.69) | 82 (4.97) | 541 (4.66) | 83 (5.63) | 205 (4.99) | 185 (3.63) | 68 (7.20) |
| **Other** |  |  |  |  |  |  |  |  |
| New fall | 8447 (6.16) | 2033 (16.66) | 262 (17.35) | 1771 (16.56) | 199 (14.84) | 581 (15.31) | 786 (16.77) | 205 (23.48) |

**B)**

|  | **NOAC vs VKA** | | **Dabigatran vs VKA** | | **Rivaroxaban vs VKA** | | **Apixaban vs VKA** | | **Edoxaban vs VKA** | |
| --- | --- | --- | --- | --- | --- | --- | --- | --- | --- | --- |
|  | **aHR^*^ (95%CI)** | **p-value** | **aHR^*^ (95%CI)** | **p-value** | **aHR^*^ (95%CI)** | **p-value** | **aHR^*^ (95%CI)** | **p-value** | **aHR^*^ (95%CI)** | **p-value** |
| **Effectiveness** |  |  |  |  |  |  |  |  |  |  |
| Stroke/SE | 0.69 (0.54-0.87) | 0.002 | 0.72 (0.51-1.03) | 0.073 | 0.72 (0.55-0.95) | 0.018 | 0.70 (0.52-0.95) | 0.020 | 0.56 (0.25-1.22) | 0.143 |
| Ischemic stroke | 0.52 (0.39-0.70) | <0.001 | 0.57 (0.37-0.88) | 0.011 | 0.64 (0.46-0.88) | 0.007 | 0.51 (0.35-0.74) | <0.001 | 0.33 (0.12-0.94) | 0.038 |
| All-cause mortality | 0.88 (0.79-0.98) | 0.026 | 0.83 (0.70-0.98) | 0.030 | 0.91 (0.80-1.03) | 0.129 | 0.88 (0.77-1.01) | 0.069 | 0.82 (0.62-1.09) | 0.168 |
| **Safety** |  |  |  |  |  |  |  |  |  |  |
| Major bleeding | 0.86 (0.72-1.03) | 0.112 | 0.92 (0.72-1.18) | 0.508 | 0.98 (0.81-1.19) | 0.831 | 0.73 (0.59-0.91) | 0.006 | 0.88 (0.58-1.34) | 0.548 |
| Intracranial bleeding | 0.82 (0.57-1.19) | 0.296 | 0.87 (0.52-1.43) | 0.573 | 1.01 (0.68-1.50) | 0.956 | 0.66 (0.42-1.05) | 0.081 | 0.88 (0.35-2.20) | 0.778 |
| Gastrointestinal bleeding | 1.03 (0.79-1.34) | 0.849 | 1.29 (0.92-1.81) | 0.144 | 1.19 (0.91-1.57) | 0.209 | 0.75 (0.54-1.05) | 0.090 | 1.10 (0.60-2.03) | 0.765 |

**C)**

|  | **Dabigatran vs Rivaroxaban** | | **Apixaban vs Rivaroxaban** | | **Edoxaban vs Rivaroxaban** | | **Apixaban vs Dabigatran** | | **Dabigatran vs Edoxaban** | | **Apixaban vs Edoxaban** | |
| --- | --- | --- | --- | --- | --- | --- | --- | --- | --- | --- | --- | --- |
|  | **aHR^*^ (95%CI)** | **p-value** | **aHR^*^ (95%CI)** | **p-value** | **aHR^*^ (95%CI)** | **p-value** | **aHR^*^ (95%CI)** | **p-value** | **aHR^*^ (95%CI)** | **p-value** | **aHR^*^ (95%CI)** | **p-value** |
| **Effectiveness** |  |  |  |  |  |  |  |  |  |  |  |  |
| Stroke/SE | 0.98 (0.72-1.34) | 0.915 | 1.09 (0.87-1.37) | 0.445 | 0.81 (0.50-1.30) | 0.384 | 1.13 (0.79-1.60) | 0.501 | 0.94 (0.49-1.80) | 0.849 | 1.22 (0.83-1.79) | 0.320 |
| Ischemic stroke | 0.89 (0.60-1.31) | 0.557 | 0.93 (0.69-1.24) | 0.603 | 0.73 (0.37-1.41) | 0.345 | 1.10 (0.70-1.74) | 0.668 | 1.52 (0.67-3.49) | 0.318 | 1.40 (0.81-2.45) | 0.230 |
| All-cause mortality | 0.86 (0.74-0.99) | 0.034 | 0.98 (0.89-1.07) | 0.626 | 0.85 (0.71-1.02) | 0.087 | 1.23 (1.05-1.45) | 0.013 | 0.71 (0.53-0.96) | 0.028 | 1.19 (1.03-1.38) | 0.019 |
| **Safety** |  |  |  |  |  |  |  |  |  |  |  |  |
| Major bleeding | 1.05 (0.85-1.30) | 0.637 | 0.78 (0.67-0.91) | 0.002 | 0.98 (0.75-1.29) | 0.882 | 0.72 (0.57-0.91) | 0.007 | 1.16 (0.77-1.73) | 0.476 | 0.70 (0.55-0.88) | 0.003 |
| Intracranial bleeding | 0.90 (0.59-1.39) | 0.647 | 0.71 (0.52-0.97) | 0.032 | 1.05 (0.58-1.92) | 0.865 | 0.82 (0.51-1.33) | 0.418 | 1.37 (0.55-3.43) | 0.496 | 0.79 (0.48-1.28) | 0.335 |
| Gastrointestinal bleeding | 1.20 (0.92-1.58) | 0.183 | 0.67 (0.54-0.83) | <0.001 | 0.94 (0.66-1.34) | 0.732 | 0.53 (0.39-0.71) | <0.001 | 1.60 (0.98-2.61) | 0.059 | 0.61 (0.44-0.84) | 0.002 |

**eTable 9:** The **A)** number of events and crude event rates per 100 person-years of outcomes in AF patients with and without a history of falls, and adjusted hazard ratios with 95% confidence intervals of outcomes compared between **B)** (individual) NOACs and VKAs, and **C)** between individual NOAC types in AF patients with a history of falls after IPTW, when restricting the study population to recently hospitalized OAC-naïve subjects with an ICD-coded hospital discharge diagnosis of AF.

^*^ Propensity scores used for stabilized IPTW were calculated with logistic regression models including 39 confounding covariates (age, sex, baseline comorbidities, medication history, clinical risk scores), stratified by calendar year. AF: atrial fibrillation; aHR: adjusted hazard ratio; CI: confidence interval; ICD: International Classification of Diseases; IPTW: inverse probability of treatment weighting; MB: major bleeding; NOAC: non-vitamin K antagonist oral anticoagulant; PY: person-year; SE: systemic embolism; VKA: vitamin K antagonist; vs: versus.

eTable 10: October 1^st^, 2016 – January 1^st^, 2019 (sensitivity analysis)

**A)**

| **Outcome** | **No previous falls**  **(n = 83,924)** | **History of falls** | | | | | | |
| --- | --- | --- | --- | --- | --- | --- | --- | --- |
|  |  | **Overall history of falls**  **(n = 9618)** | **VKA**  **(n = 1106)** | **NOAC**  **(n = 8512)** | **Dabigatran**  **(n = 657)** | **Rivaroxaban**  **(n = 1750)** | **Apixaban**  **(n = 3971)** | **Edoxaban**  **(n = 2134)** |
|  | **Events (per 100 PY)** | **Events (per 100 PY)** | **Events (per 100 PY)** | **Events (per 100 PY)** | **Events (per 100 PY)** | **Events (per 100 PY)** | **Events (per 100 PY)** | **Events (per 100 PY)** |
| **Effectiveness** |  |  |  |  |  |  |  |  |
| Stroke/SE | 1498 (2.52) | 241 (4.30) | 23 (5.27) | 218 (4.22) | 15 (3.66) | 52 (4.72) | 108 (4.39) | 43 (3.60) |
| Ischemic stroke | 725 (1.21) | 124 (2.19) | 10 (2.26) | 114 (2.19) | 11 (2.66) | 24 (2.16) | 59 (2.38) | 20 (1.66) |
| All-cause mortality | 4999 (8.32) | 1553 (27.23) | 127 (28.51) | 1426 (27.12) | 72 (17.28) | 307 (27.37) | 743 (29.62) | 304 (25.09) |
| **Safety** |  |  |  |  |  |  |  |  |
| Major bleeding | 3250 (5.53) | 569 (10.36) | 60 (13.97) | 509 (10.06) | 46 (11.59) | 124 (11.48) | 204 (8.44) | 135 (11.57) |
| Intracranial bleeding | 588 (0.98) | 129 (2.28) | 11 (2.49) | 118 (2.26) | 11 (2.66) | 28 (2.52) | 48 (1.93) | 31 (2.58) |
| Gastrointestinal bleeding | 1796 (3.03) | 308 (5.50) | 27 (6.17) | 281 (5.45) | 33 (8.20) | 72 (6.54) | 103 (4.18) | 73 (6.15) |
| **Other** |  |  |  |  |  |  |  |  |
| New fall | 3604 (6.18) | 1048 (20.19) | 100 (25.13) | 948 (19.78) | 72 (19.14) | 204 (20.03) | 444 (19.38) | 228 (20.58) |

**B)**

|  | **NOAC vs VKA** | | **Dabigatran vs VKA** | | **Rivaroxaban vs VKA** | | **Apixaban vs VKA** | | **Edoxaban vs VKA** | |
| --- | --- | --- | --- | --- | --- | --- | --- | --- | --- | --- |
|  | **aHR^*^ (95%CI)** | **p-value** | **aHR^*^ (95%CI)** | **p-value** | **aHR^*^ (95%CI)** | **p-value** | **aHR^*^ (95%CI)** | **p-value** | **aHR^*^ (95%CI)** | **p-value** |
| **Effectiveness** |  |  |  |  |  |  |  |  |  |  |
| Stroke/SE | 0.77 (0.44-1.36) | 0.371 | 0.50 (0.23-1.08) | 0.077 | 1.16 (0.68-1.97) | 0.594 | 0.74 (0.41-1.32) | 0.306 | 0.60 (0.33-1.08) | 0.089 |
| Ischemic stroke | 0.61 (0.28-1.32) | 0.207 | 0.76 (0.28-2.07) | 0.592 | 1.00 (0.45-2.22) | 0.991 | 0.62 (0.28-1.38) | 0.239 | 0.39 (0.17-0.91) | 0.030 |
| All-cause mortality | 0.76 (0.62-0.94) | 0.011 | 0.68 (0.47-0.99) | 0.043 | 0.84 (0.67-1.06) | 0.148 | 0.81 (0.65-1.01) | 0.066 | 0.75 (0.60-0.95) | 0.016 |
| **Safety** |  |  |  |  |  |  |  |  |  |  |
| Major bleeding | 0.71 (0.50-0.99) | 0.041 | 0.88 (0.56-1.37) | 0.562 | 0.92 (0.65-1.29) | 0.617 | 0.60 (0.42-0.85) | 0.004 | 0.89 (0.63-1.27) | 0.536 |
| Intracranial bleeding | 0.67 (0.32-1.37) | 0.271 | 0.86 (0.35-2.12) | 0.741 | 0.90 (0.43-1.90) | 0.786 | 0.61 (0.29-1.30) | 0.202 | 0.93 (0.45-1.95) | 0.856 |
| Gastrointestinal bleeding | 0.81 (0.49-1.33) | 0.407 | 1.28 (0.71-2.32) | 0.406 | 1.07 (0.65-1.76) | 0.798 | 0.61 (0.36-1.03) | 0.064 | 1.03 (0.61-1.74) | 0.914 |

**C)**

|  | **Dabigatran vs Rivaroxaban** | | **Apixaban vs Rivaroxaban** | | **Edoxaban vs Rivaroxaban** | | **Apixaban vs Dabigatran** | | **Dabigatran vs Edoxaban** | | **Apixaban vs Edoxaban** | |
| --- | --- | --- | --- | --- | --- | --- | --- | --- | --- | --- | --- | --- |
|  | **aHR^*^ (95%CI)** | **p-value** | **aHR^*^ (95%CI)** | **p-value** | **aHR^*^ (95%CI)** | **p-value** | **aHR^*^ (95%CI)** | **p-value** | **aHR^*^ (95%CI)** | **p-value** | **aHR^*^ (95%CI)** | **p-value** |
| **Effectiveness** |  |  |  |  |  |  |  |  |  |  |  |  |
| Stroke/SE | 0.59 (0.32-1.08) | 0.088 | 0.84 (0.59-1.18) | 0.316 | 0.68 (0.45-1.03) | 0.067 | 1.44 (0.81-2.56) | 0.212 | 0.89 (0.47-1.67) | 0.722 | 1.29 (0.90-1.85) | 0.172 |
| Ischemic stroke | 0.86 (0.40-1.84) | 0.696 | 0.96 (0.59-1.56) | 0.856 | 0.68 (0.37-1.24) | 0.206 | 1.09 (0.55-2.15) | 0.809 | 1.47 (0.67-3.26) | 0.340 | 1.56 (0.92-2.64) | 0.099 |
| All-cause mortality | 0.68 (0.51-0.91) | 0.008 | 0.99 (0.86-1.14) | 0.881 | 0.80 (0.68-0.94) | 0.007 | 1.47 (1.12-1.93) | 0.006 | 0.79 (0.59-1.04) | 0.098 | 1.24 (1.08-1.42) | 0.002 |
| **Safety** |  |  |  |  |  |  |  |  |  |  |  |  |
| Major bleeding | 1.01 (0.69-1.46) | 0.965 | 0.75 (0.59-0.94) | 0.013 | 0.98 (0.76-1.27) | 0.900 | 0.72 (0.50-1.04) | 0.076 | 1.09 (0.75-1.59) | 0.648 | 0.74 (0.59-0.92) | 0.008 |
| Intracranial bleeding | 0.83 (0.39-1.78) | 0.636 | 0.78 (0.49-1.26) | 0.315 | 1.03 (0.61-1.74) | 0.905 | 0.84 (0.41-1.73) | 0.640 | 1.22 (0.54-2.75) | 0.640 | 0.80 (0.50-1.26) | 0.330 |
| Gastrointestinal bleeding | 1.32 (0.84-2.08) | 0.226 | 0.66 (0.48-0.90) | 0.009 | 0.93 (0.66-1.31) | 0.677 | 0.47 (0.30-0.73) | <0.001 | 1.55 (0.98-2.45) | 0.060 | 0.67 (0.50-0.91) | 0.011 |

**eTable 10:** The **A)** number of events and crude event rates per 100 person-years of outcomes in AF patients with and without a history of falls, and adjusted hazard ratios with 95% confidence intervals of outcomes compared between **B)** (individual) NOACs and VKAs, and **C)** between individual NOAC types in AF patients with a history of falls after IPTW in subgroup of subjects having initiated treatment between October 1^st^, 2016 and January 1^st^, 2019.

^*^ Propensity scores used for stabilized IPTW were calculated with logistic regression models including 39 confounding covariates (age, sex, baseline comorbidities, medication history, clinical risk scores), stratified by calendar year. aHR: adjusted hazard ratio; CI: confidence interval; IPTW: inverse probability of treatment weighting; MB: major bleeding; NOAC: non-vitamin K antagonist oral anticoagulant; PY: person-year; SE: systemic embolism; VKA: vitamin K antagonist; vs: versus.

# Supplemental figures

## eFigure 1: Overview of study design

**
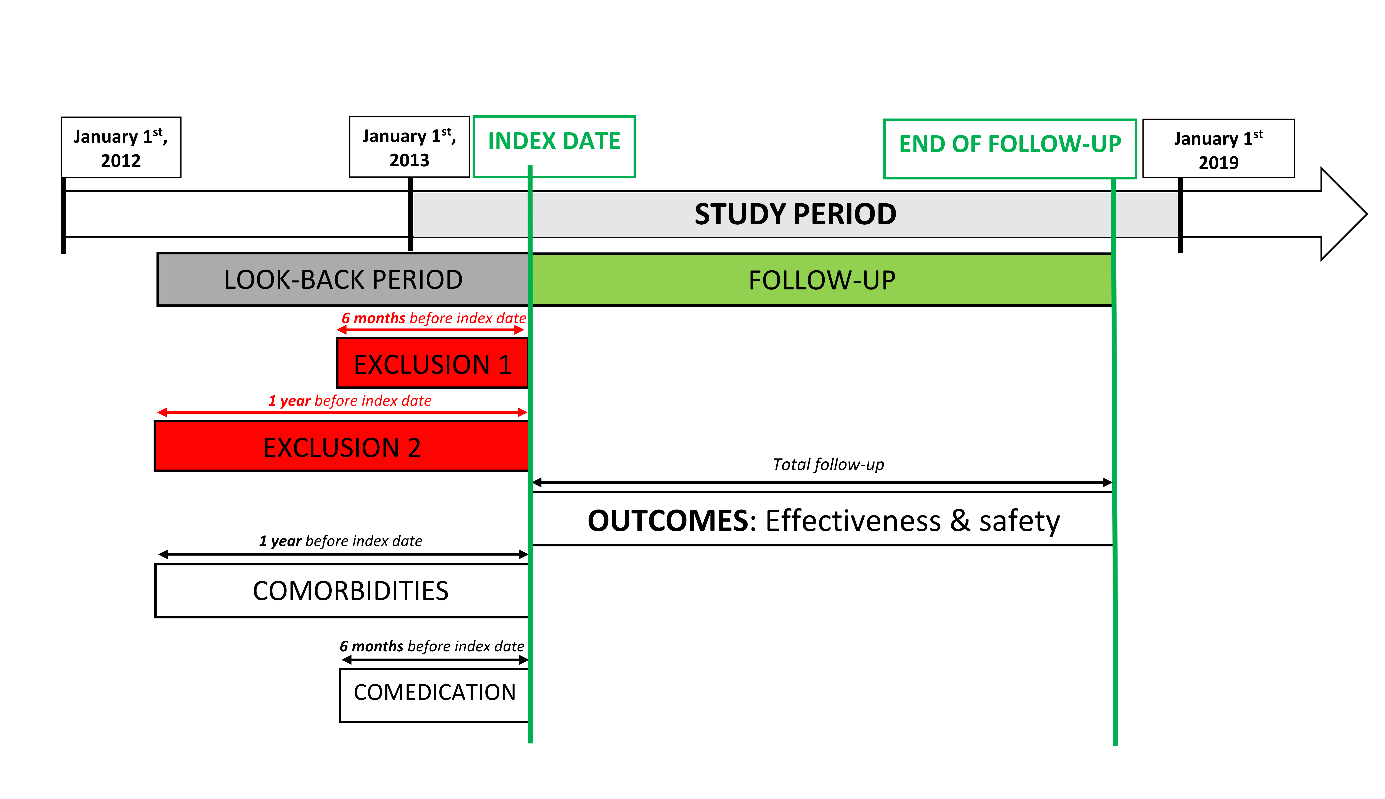
**

**eFigure 1:** Overview of study design.

**Study period:** January 1^st^, 2013 – January 1^st^, 2019. **Look-back period:** Up to 1 year before index date (maximum up until January 1^st^, 2012). **Index date:** First dispensing of oral anticoagulant (NOAC or VKA) during study period to subjects ≥45 years old. **End of follow-up:** Patients were followed from OAC initiation until the first occurrence of the investigated outcome, discontinuation (>60-day gap of drug supply) or switch (dispensing of another OAC) of treatment, death, emigration or end of the study period (January 1^st^, 2019), whichever came first (on-treatment analysis). **Exclusion 1:** Patients with total hip or knee replacement surgery, or diagnosis of deep vein thrombosis or pulmonary embolism ≤6 months before the index date were excluded. **Exclusion 2:** Only OAC-naïve subjects were included, excluding OAC-experienced subjects with an OAC prescription filled ≤1 year before the index date. Moreover, subjects with valvular AF (mechanical prosthetic heart valve or moderate/severe mitral stenosis), end-stage renal disease (chronic kidney disease stage V and/or dialysis), subjects with ≥2 prescription claims of different oral anticoagulant types or dosages on the index date, or subjects treated with NOAC dosages not approved for stroke prevention in AF (e.g. rivaroxaban 10 mg) were excluded. **Comorbidities:** Comorbidities were identified using specific ICD-coded diagnoses (e.g. cancer) from the MHD, medical procedure codes (e.g. cancer-related surgery) from the IMA database and/or ATC-coded prescription claims (e.g. antineoplastic drugs) from the IMA database ≤1 year before the index date. **Comedication use:** Comedication dispensed up to 6 months before the index date. **Outcome:** Effectiveness and safety of oral anticoagulants in patients with AF and a history of falls.

AF: Atrial fibrillation; ATC: Anatomical Therapeutic Chemical Classification; IMA: InterMutualistic Agency; MHD: Minimal Hospital Dataset; NOAC: non-vitamin K antagonist oral anticoagulant; OAC: oral anticoagulant; vitamin K antagonist.

## eFigure 2: Flowchart of study population


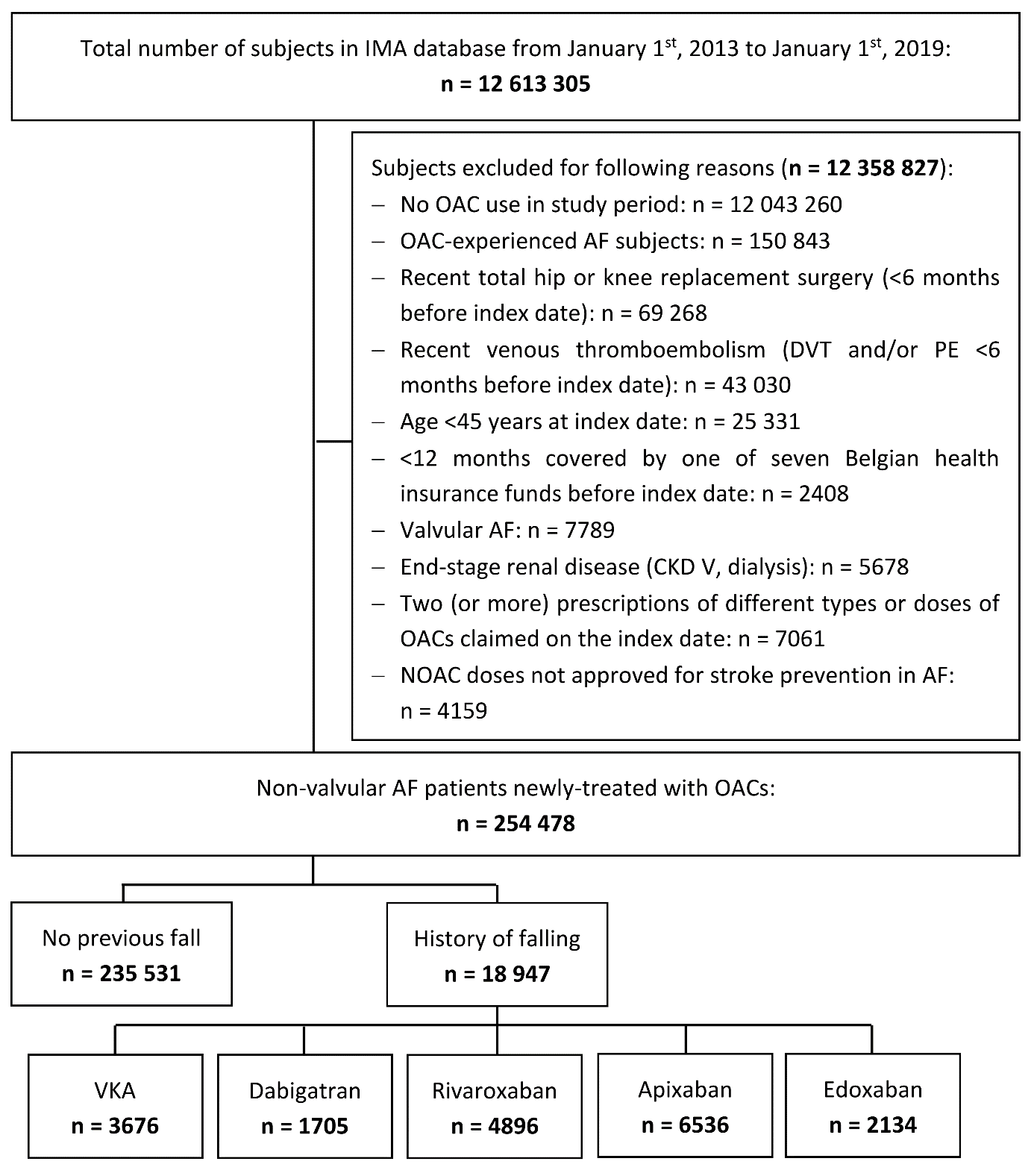


**eFigure 2:** Flowchart of study population.

AF: atrial fibrillation; CKD: chronic kidney disease; DVT: deep vein thrombosis; IMA: InterMutualistic Agency; NOAC: non-vitamin K antagonist oral anticoagulant; OAC: oral anticoagulant; PE: pulmonary embolism; VKA: vitamin K antagonist.

## eFigure 3: Love plots (main analysis)

**A)**

**
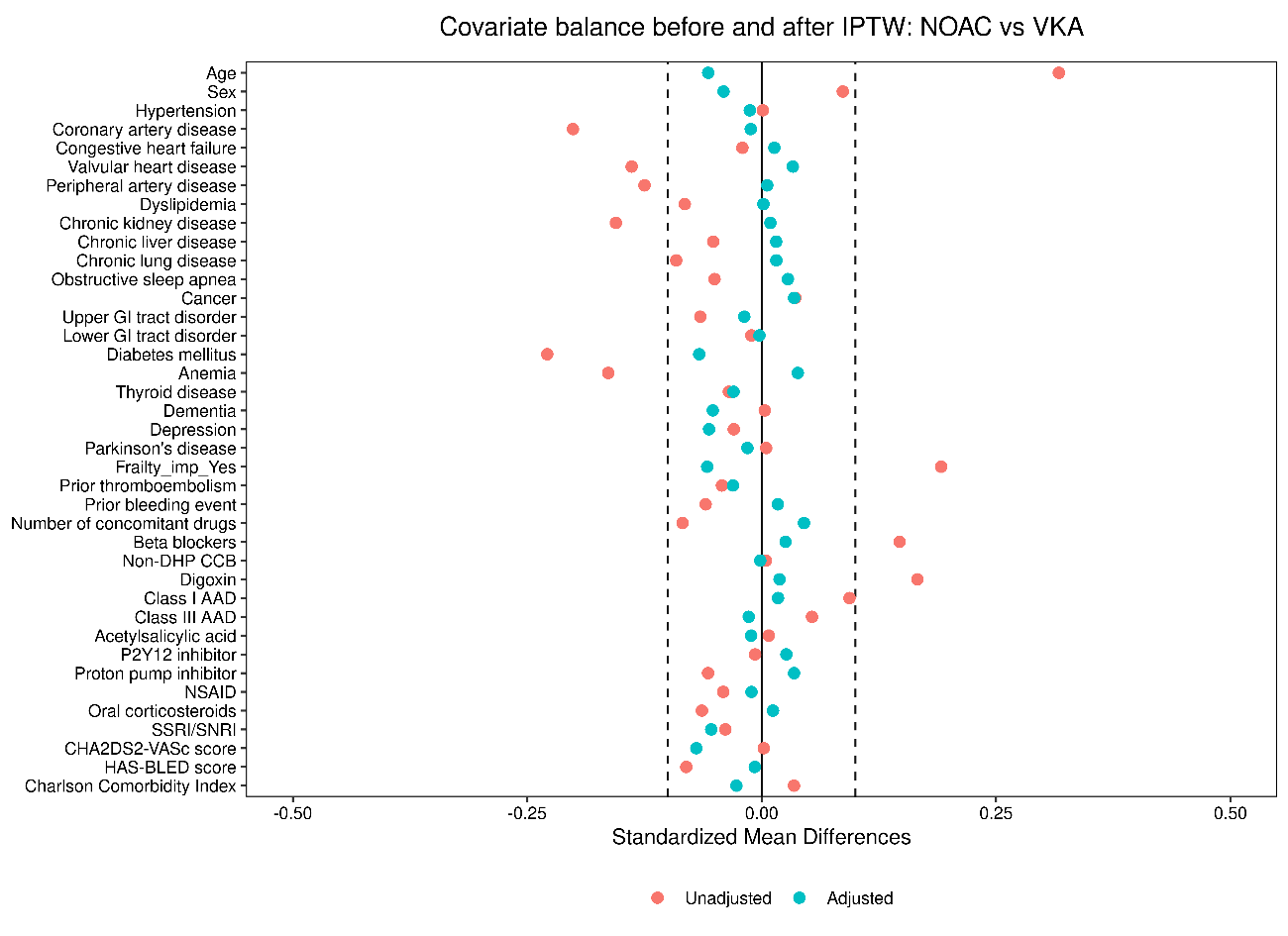
**

**B)**

**
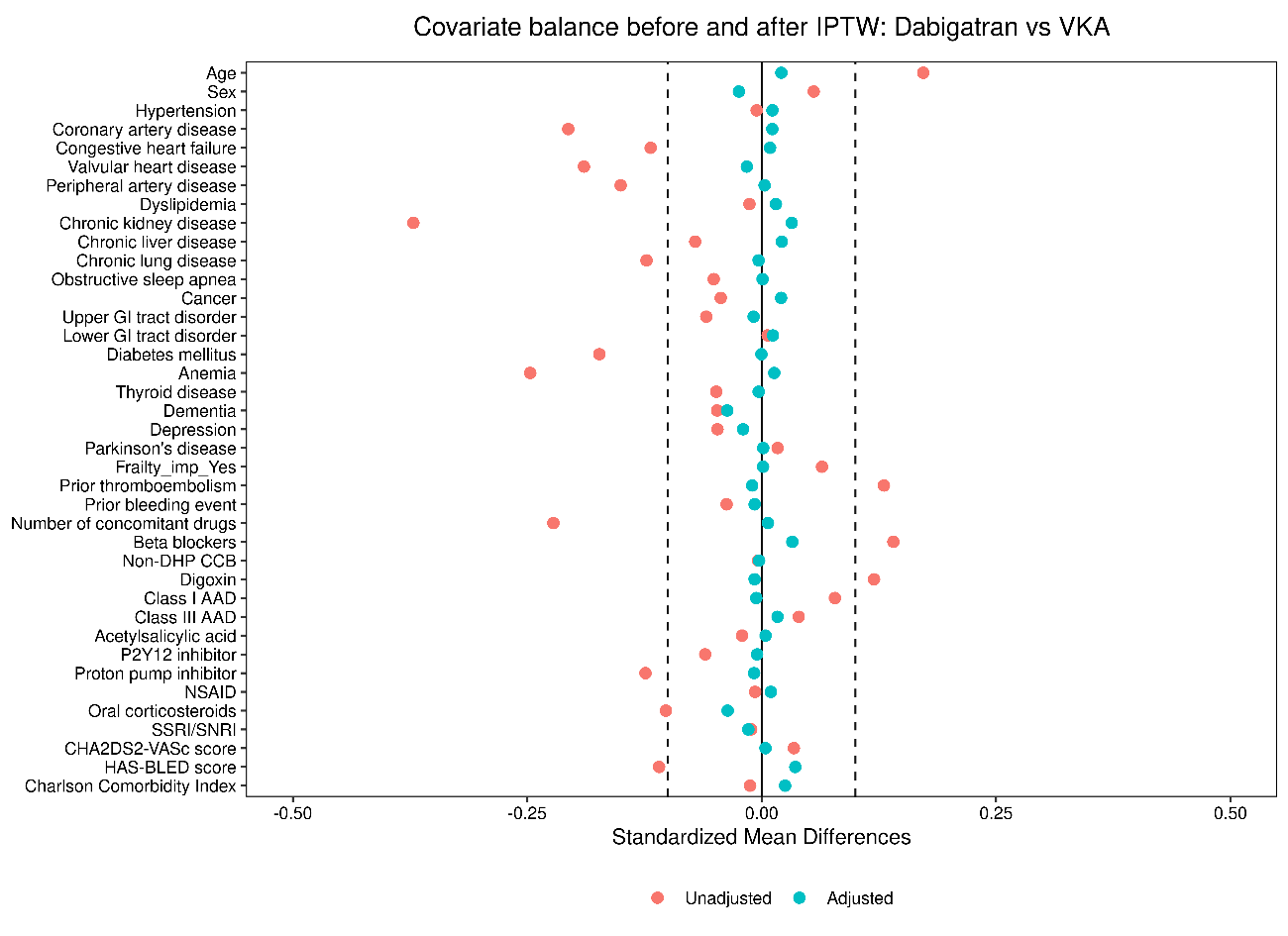
**

**C)**

**
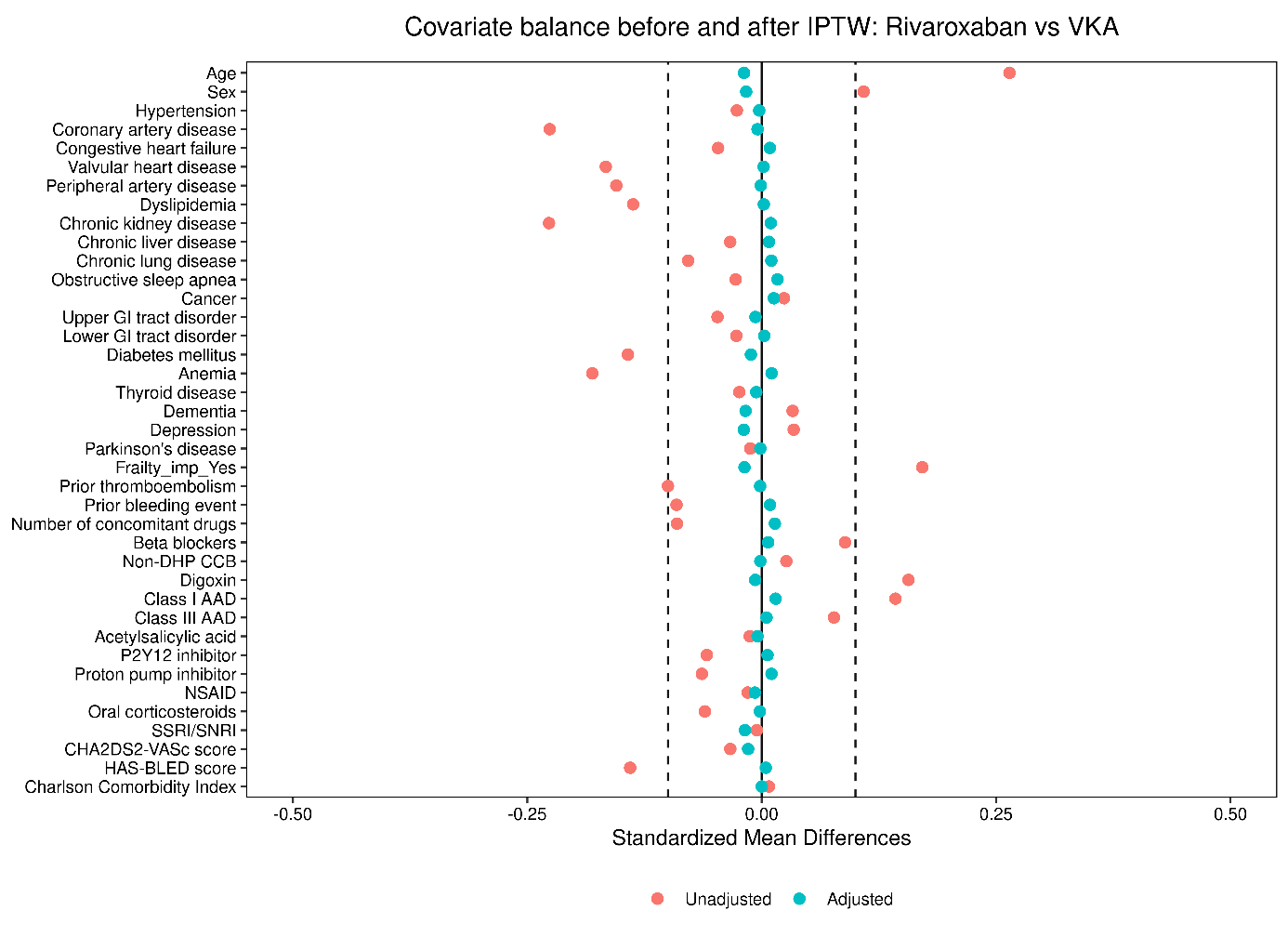
**

**D)**

**
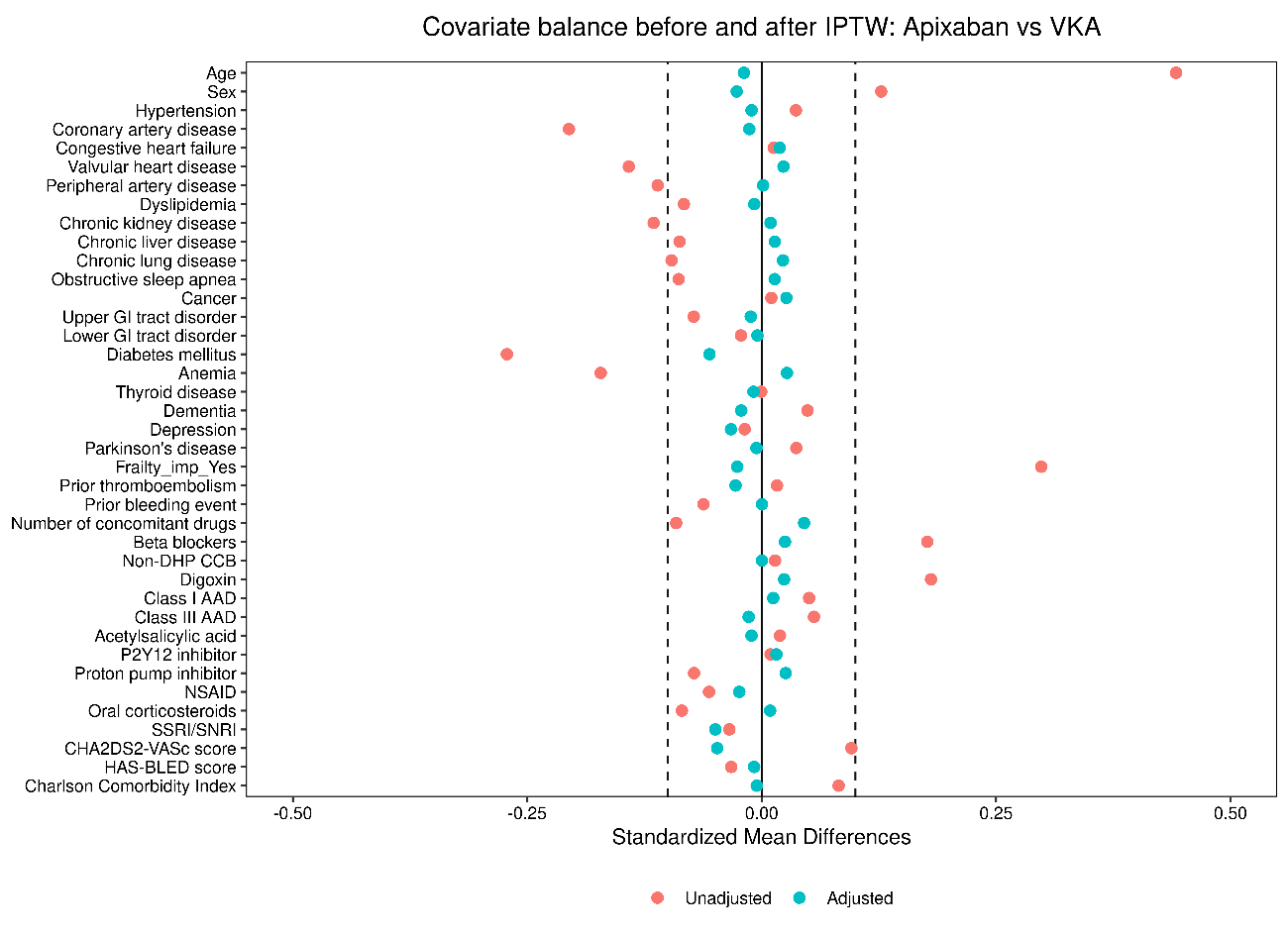
**

**E)**


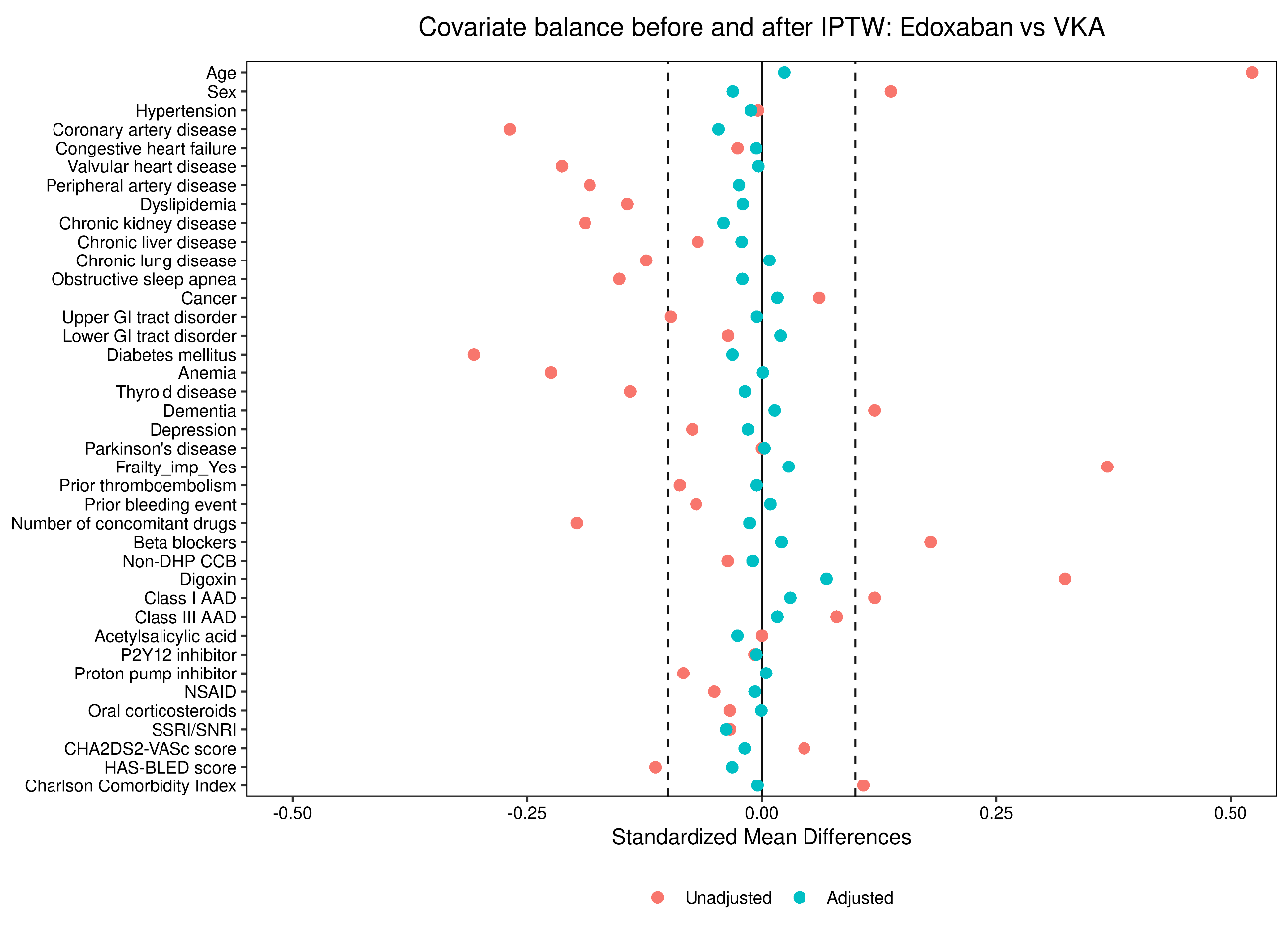


**F)**

**
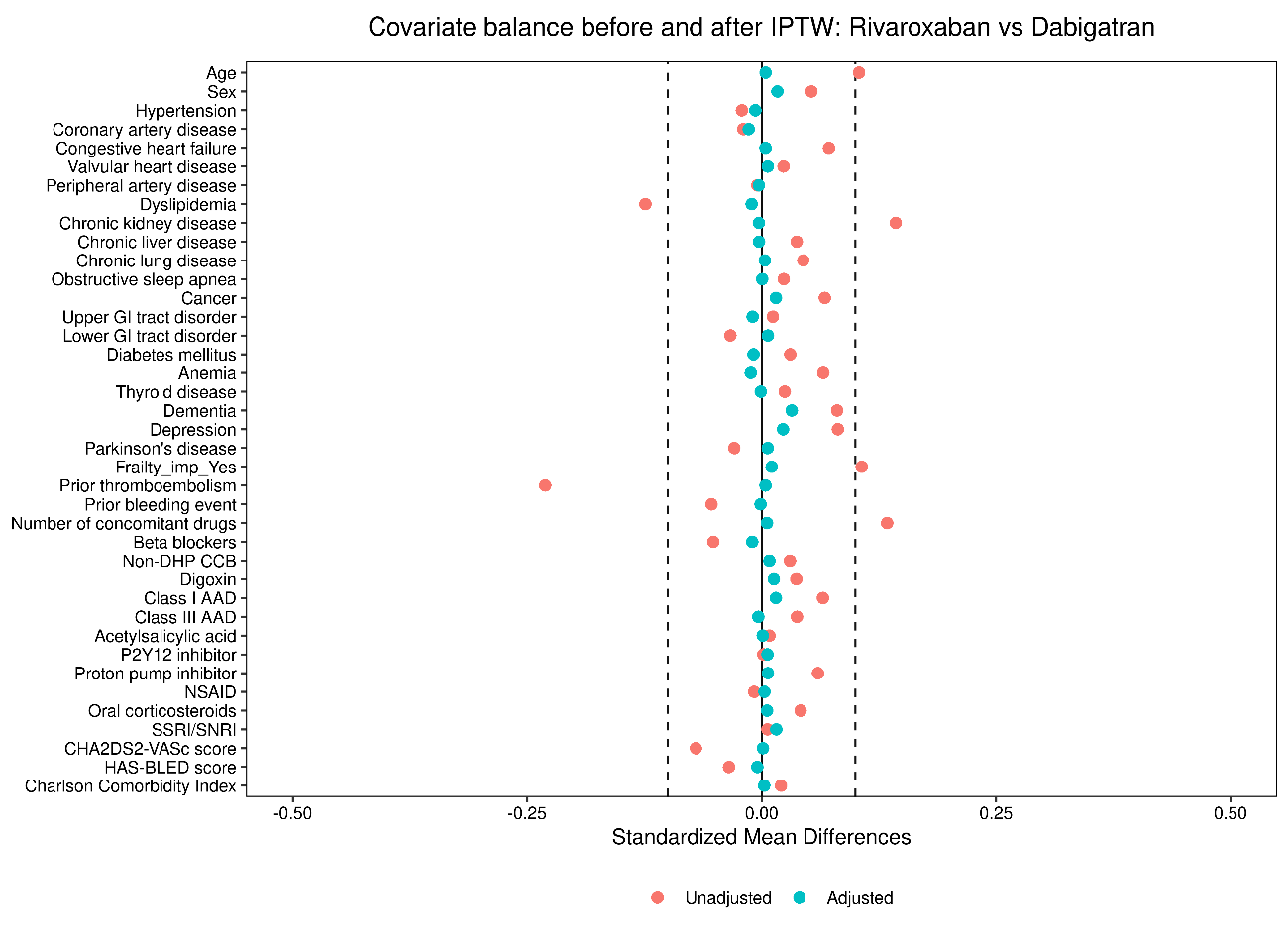
**

**G)**


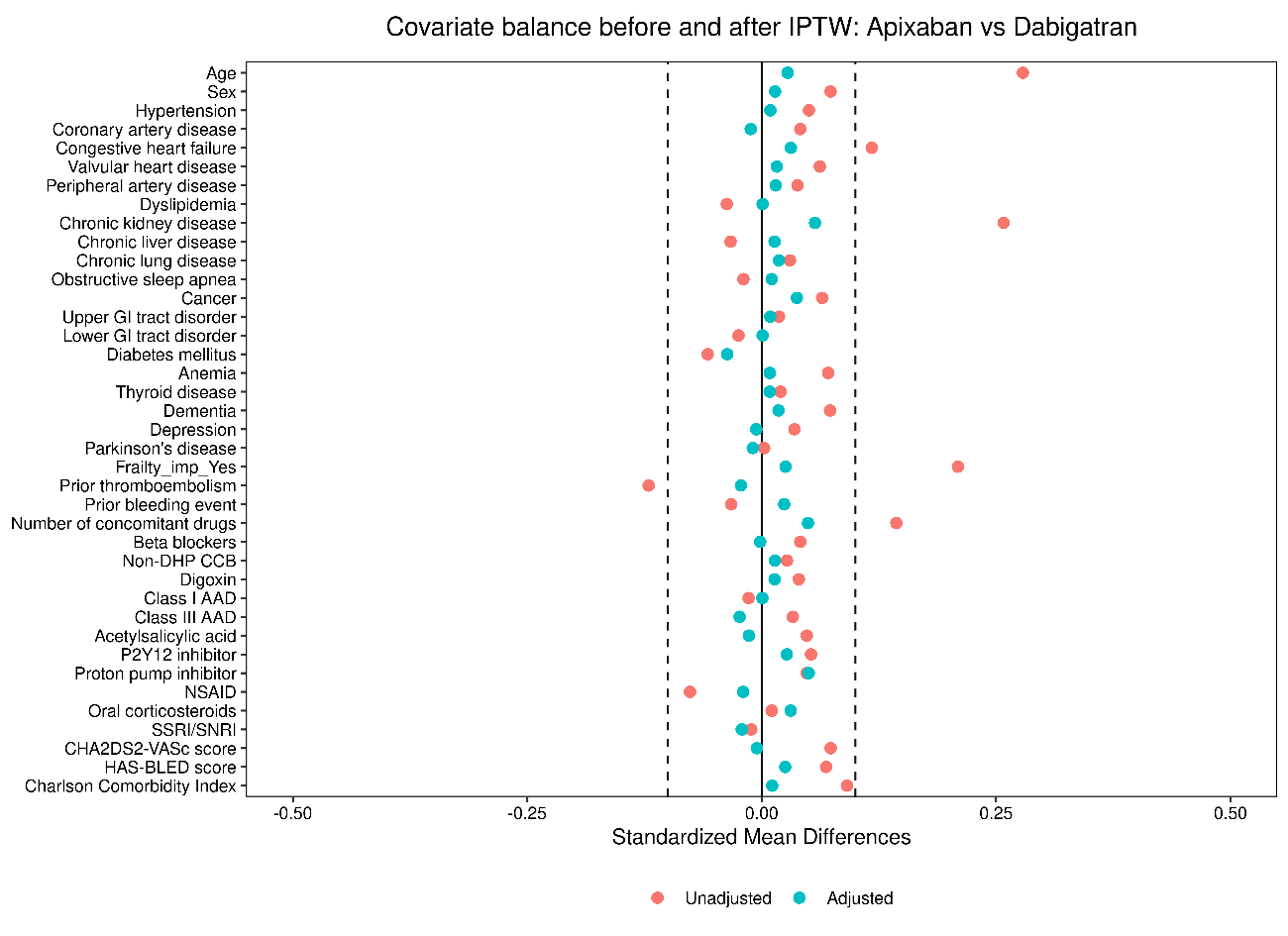


**H)**

**
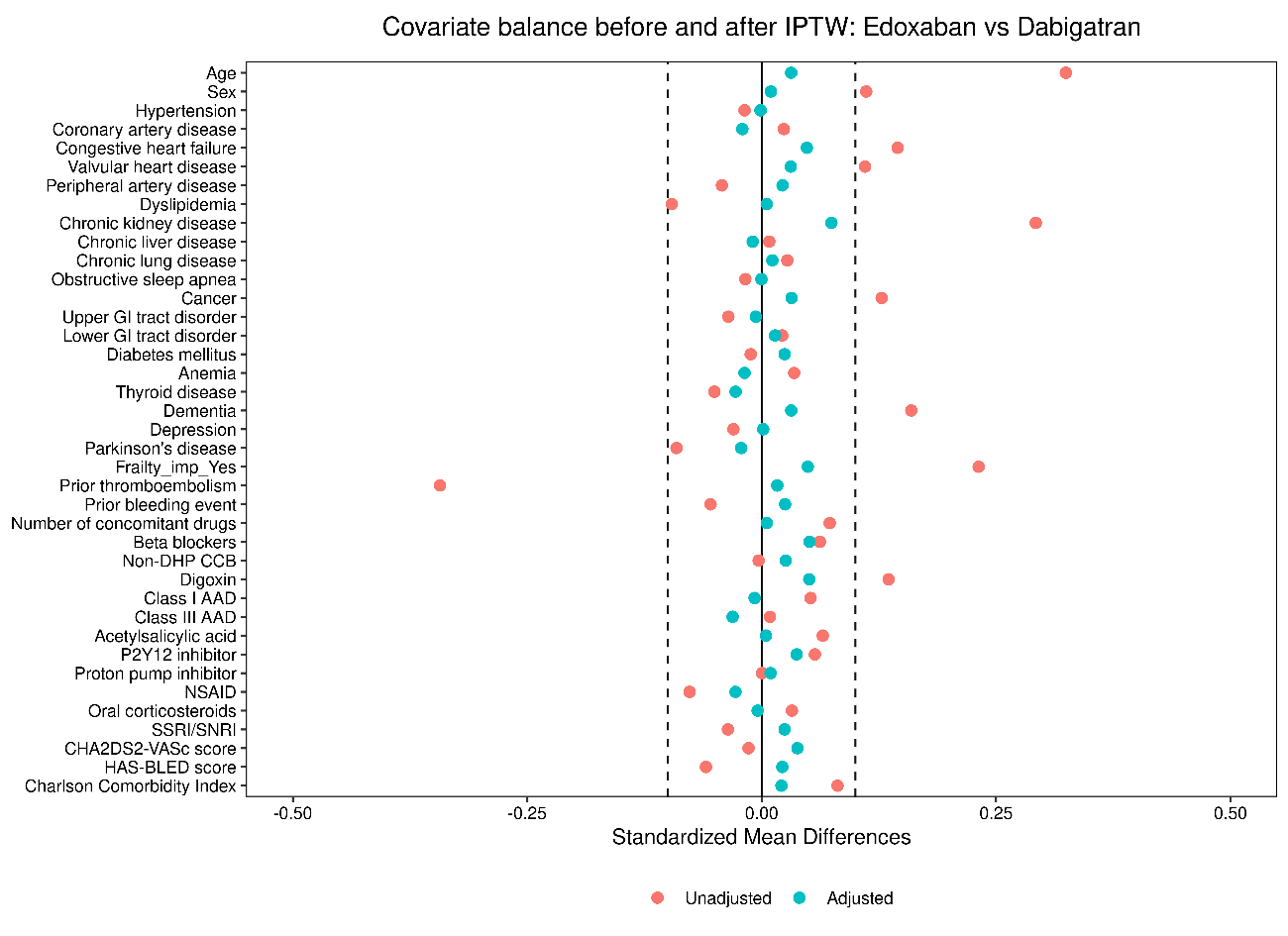
**

**I)**

**
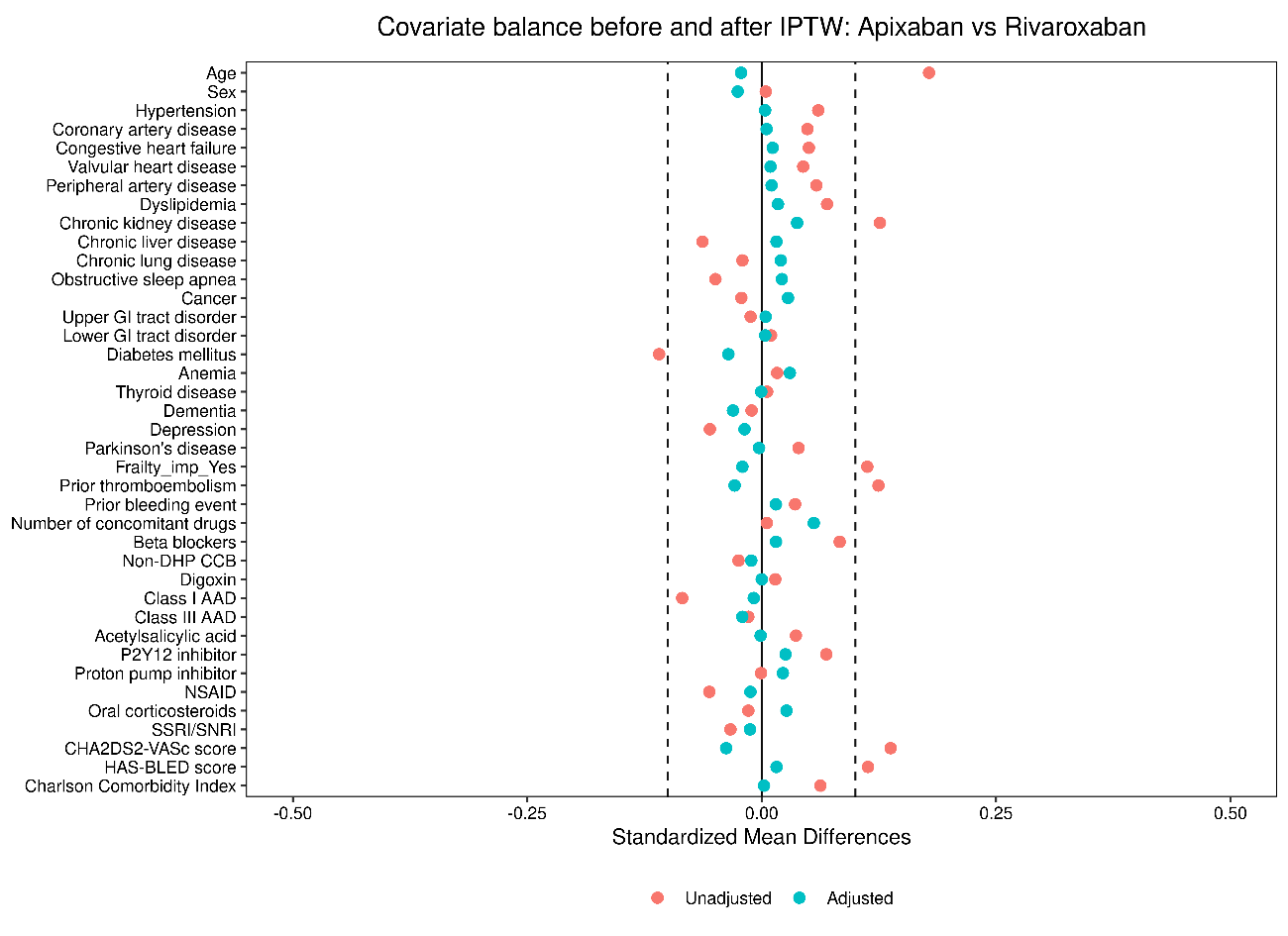
**

**J)**


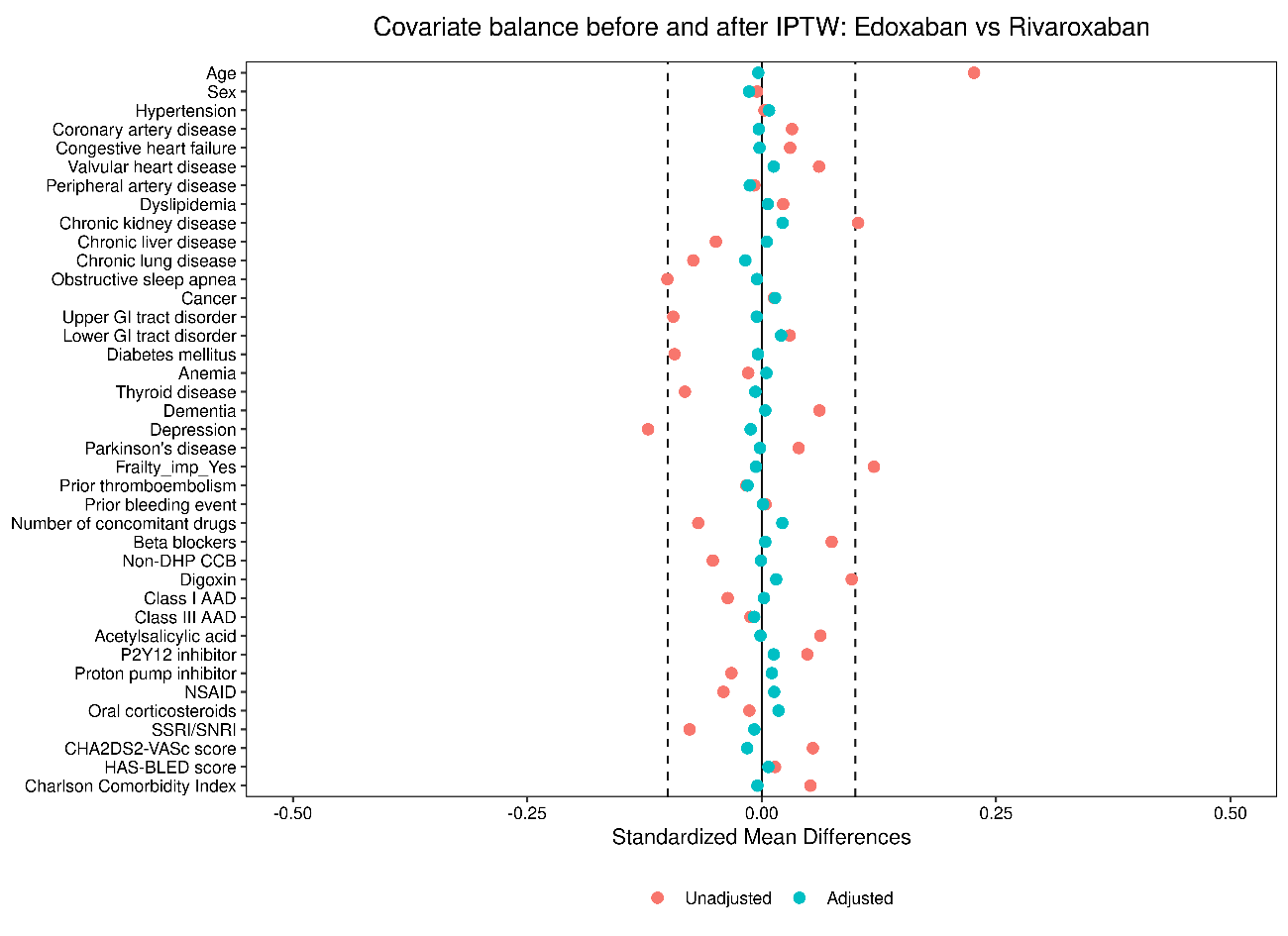


**K)**

**
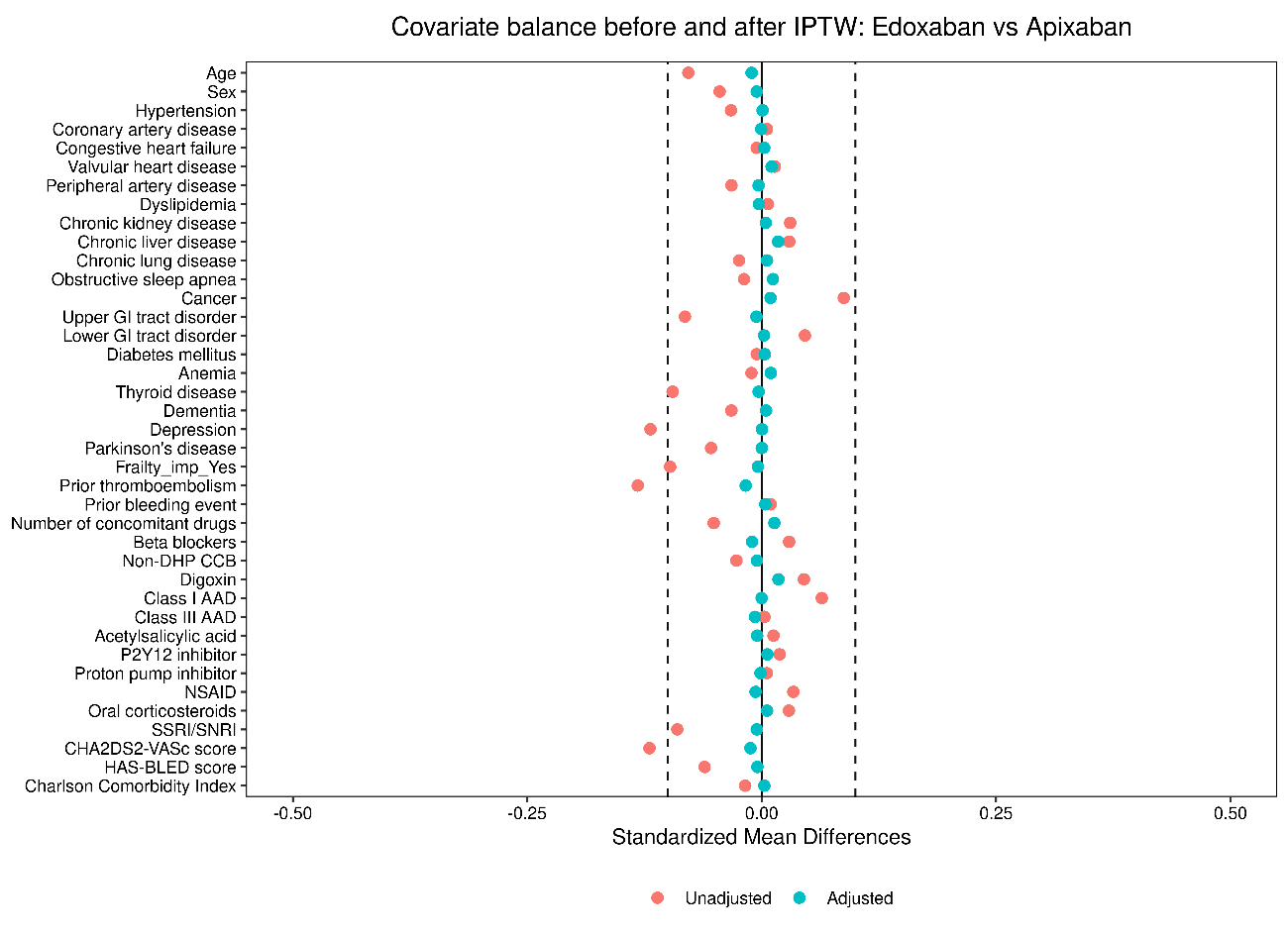
**

**eFigure 3:** Love plots illustrating covariate balance before and after IPTW when comparing **A)** NOACs to VKAs, **B)** dabigatran to VKAs, **C)** rivaroxaban to VKAs, **D)** apixaban to VKAs, **E)** edoxaban to VKAs, **F)** rivaroxaban to dabigatran, **G)** apixaban to dabigatran, **H)** edoxaban to dabigatran, **I)** apixaban to rivaroxaban, **J)** edoxaban to rivaroxaban, and **K)** edoxaban to apixaban.

AAD: antiarrhythmic drug; GI: gastrointestinal; IPTW: inverse probability of treatment weighting; NOAC: non-vitamin K antagonist oral anticoagulant; NSAID: non-steroidal anti-inflammatory drug; OAC: oral anticoagulant; SE: systemic embolism; SNRI: serotonin and norepinephrine reuptake inhibitor; SSRI: selective serotonin reuptake inhibitor; VKA: vitamin K antagonist; vs: versus.

eFigure 4: Forest plot (intention-to-treat analysis)

**A)**

**
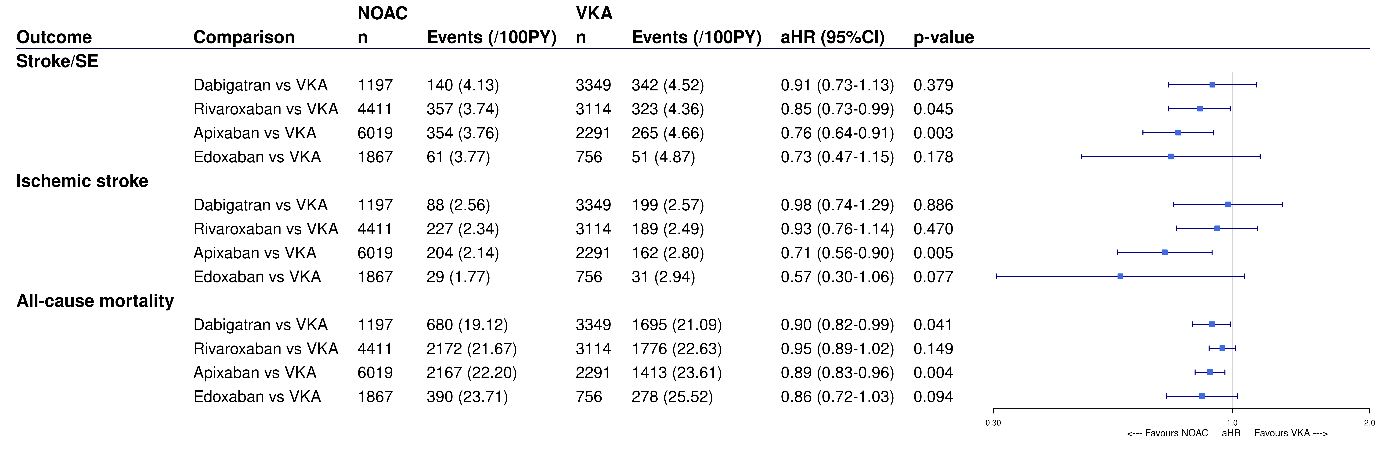
**

**
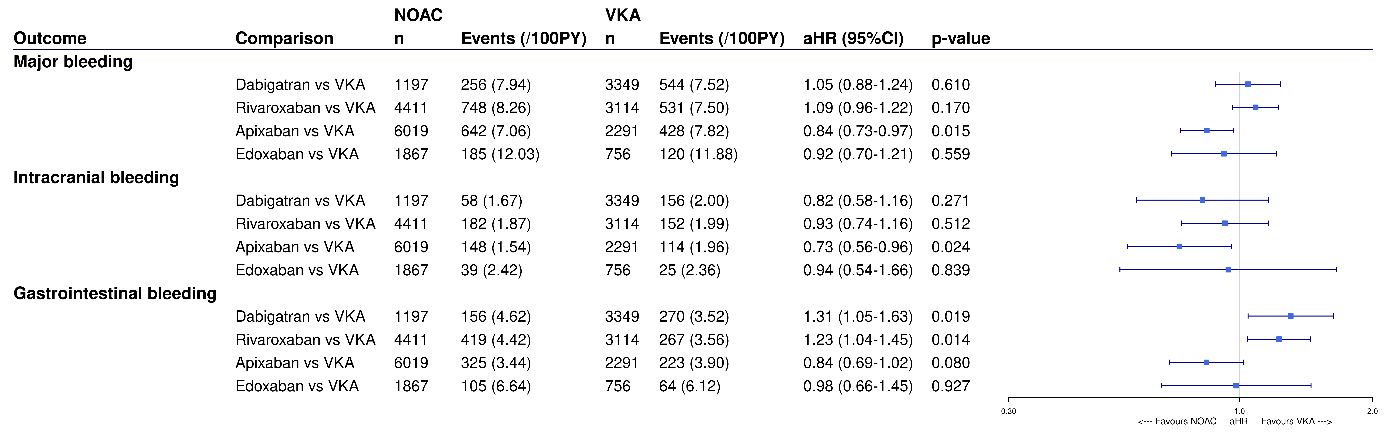
**

**B)**

**
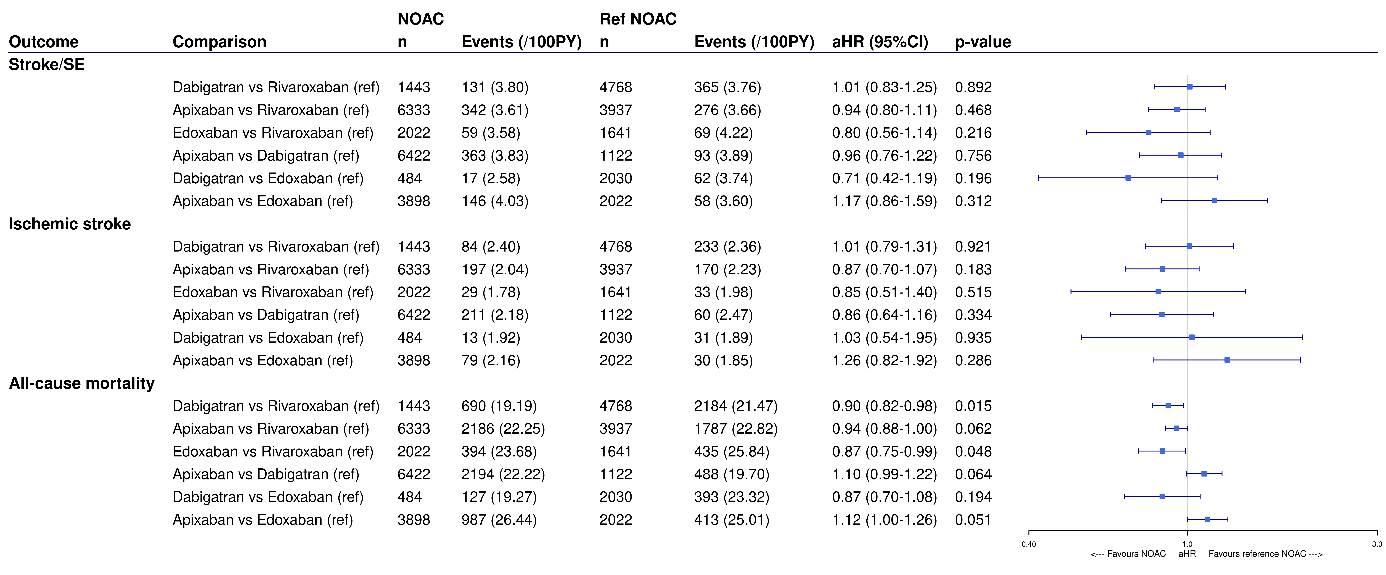
**

**
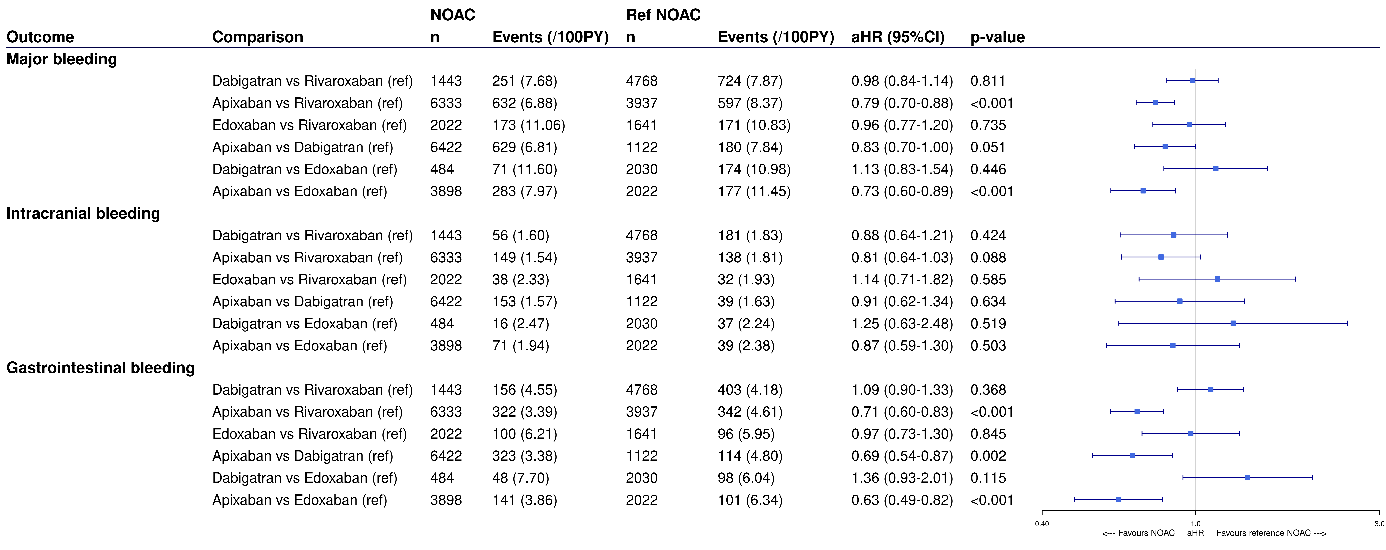
**

**eFigure 4:** The effectiveness and safety of **A)** NOACs versus VKAs, and **B)** between individual NOAC types in AF patients with a history of falls after IPTW using an intention-to-treatment analysis. In this approach, the end of follow-up was defined as the first occurrence of an outcome, death, emigration or end of study period, whichever occurred first. The weighted number of subjects at risk in the pseudopopulation, weighted number of events, weighted event rates per 100 PY and adjusted HRs with 95%CIs after IPTW are illustrated. aHR: adjusted hazard ratio; CI: confidence interval; IPTW: inverse probability of treatment weighting; NOAC: non-vitamin K antagonist oral anticoagulant; PY: person-years; Ref: reference category; SE: systemic embolism; VKA: vitamin K antagonist; vs: versus.

eFigure 5: Forest plot (patients with ICD-coded hospital discharge diagnosis of AF)

**A)**

**
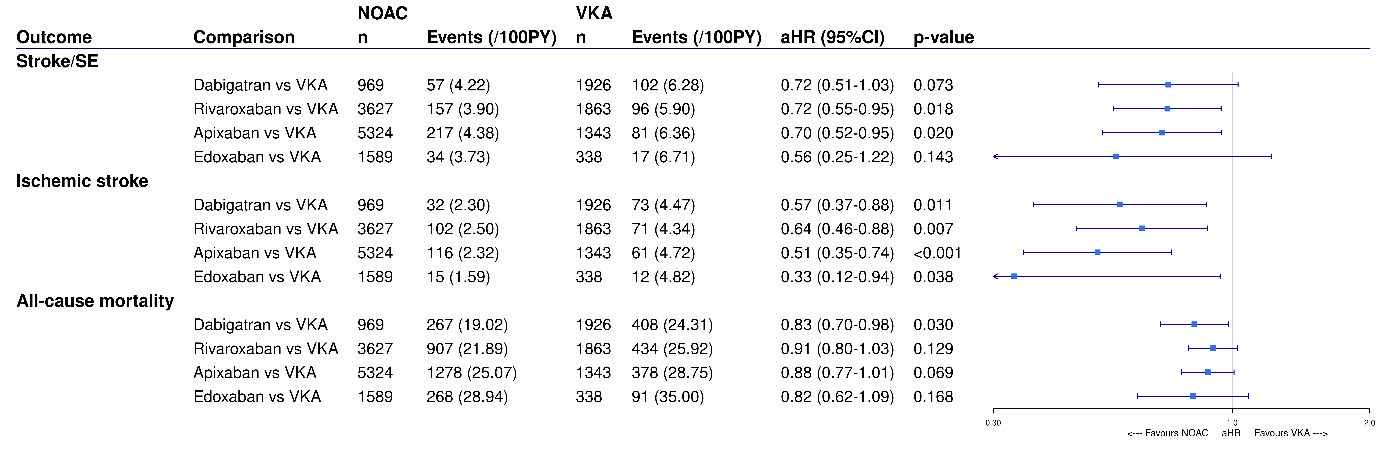
**

**
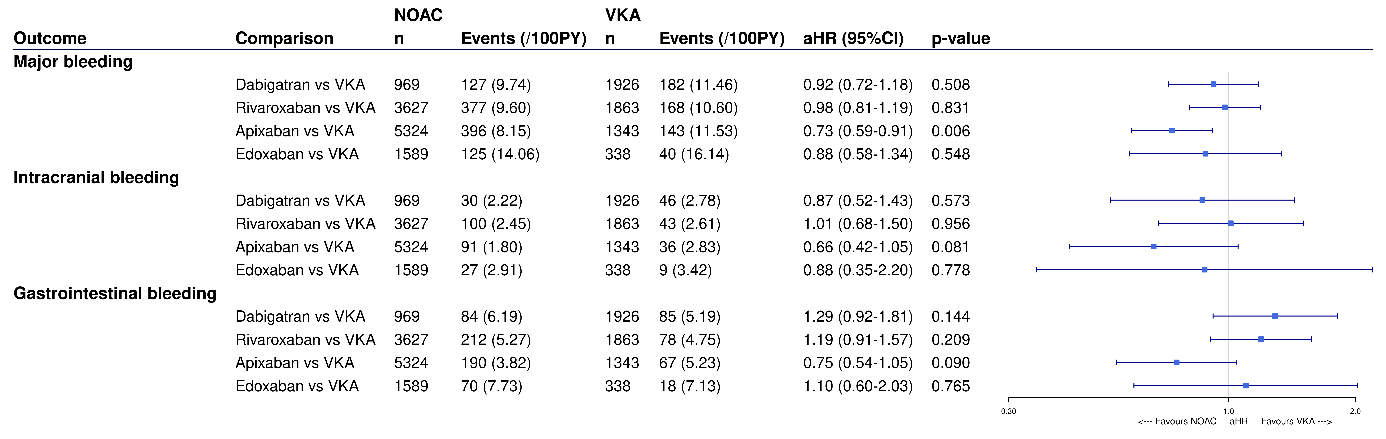
**

**B)**

**
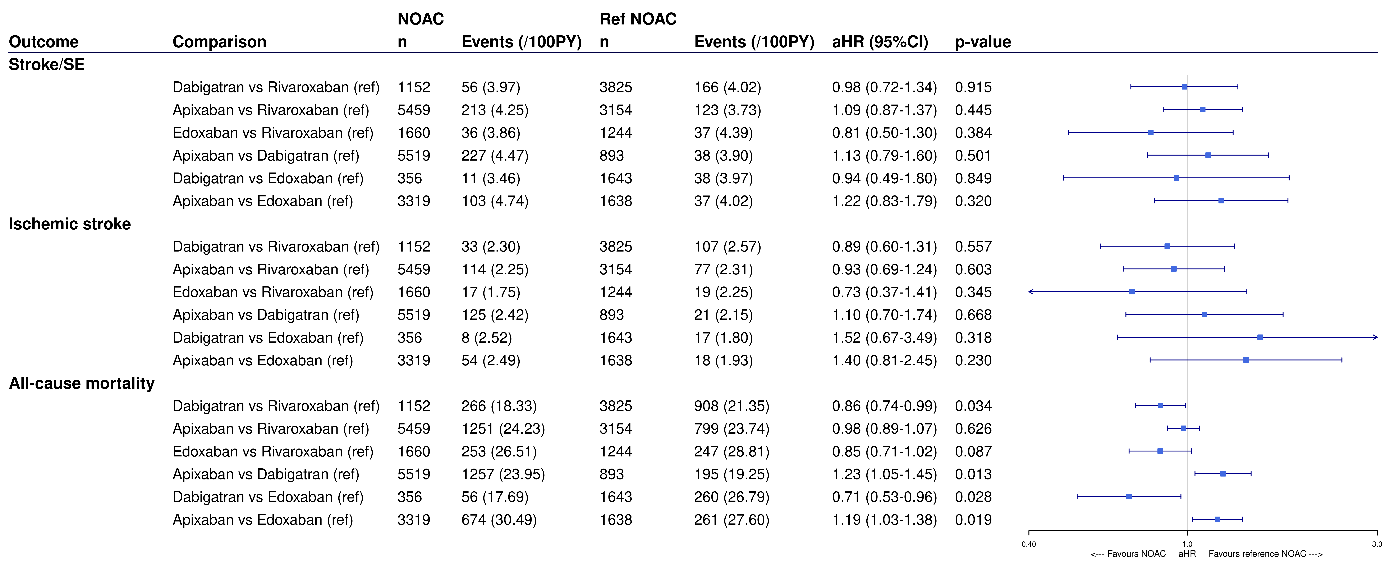
**

**
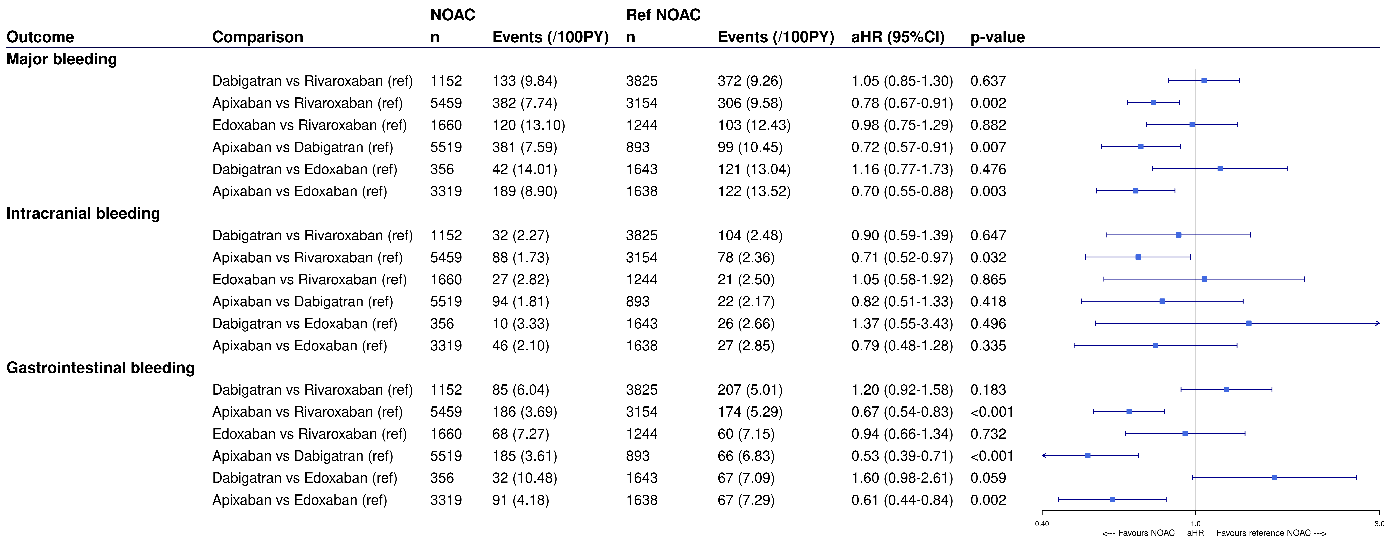
**

**eFigure 5:** The effectiveness and safety of **A)** NOACs versus VKAs, and **B)** between individual NOAC types in AF patients with a history of falls after IPTW, when restricting the study population to recently hospitalized OAC-naïve subjects with an ICD-coded hospital discharge diagnosis of AF. The weighted number of subjects at risk in the pseudopopulation, weighted number of events, weighted event rates per 100 PY and adjusted HRs with 95%CIs after IPTW are illustrated. AF: atrial fibrillation; aHR: adjusted hazard ratio; CI: confidence interval; ICD: International Classification of Diseases; IPTW: inverse probability of treatment weighting; NOAC: non-vitamin K antagonist oral anticoagulant; PY: person-years; Ref: reference category; SE: systemic embolism; VKA: vitamin K antagonist; vs: versus.

eFigure 6: Forest plot (October 1^st^, 2016 – January 1^st^, 2019)

**A)**

**
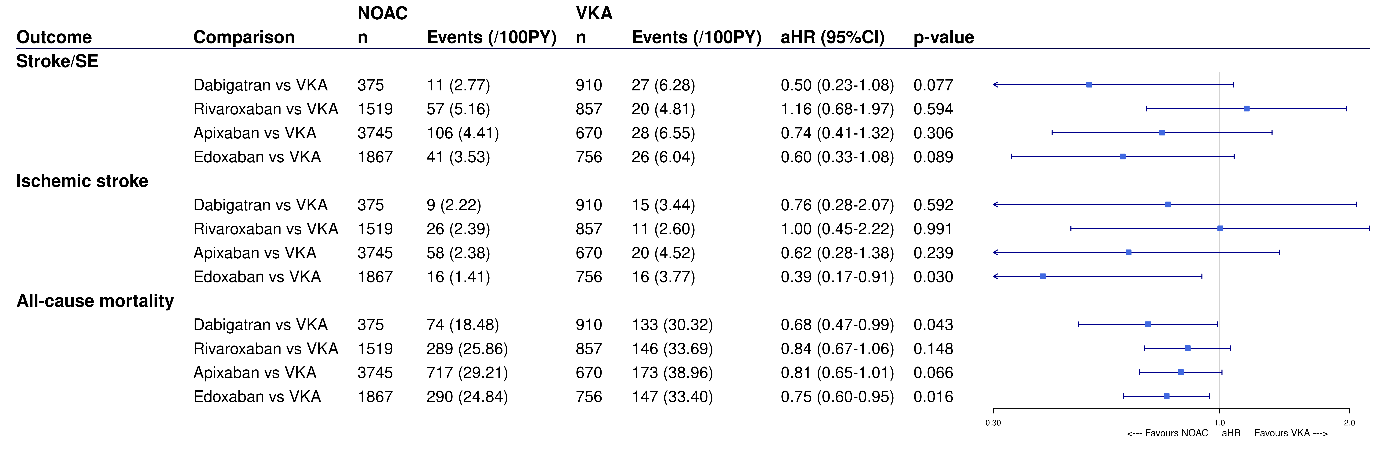
**

**
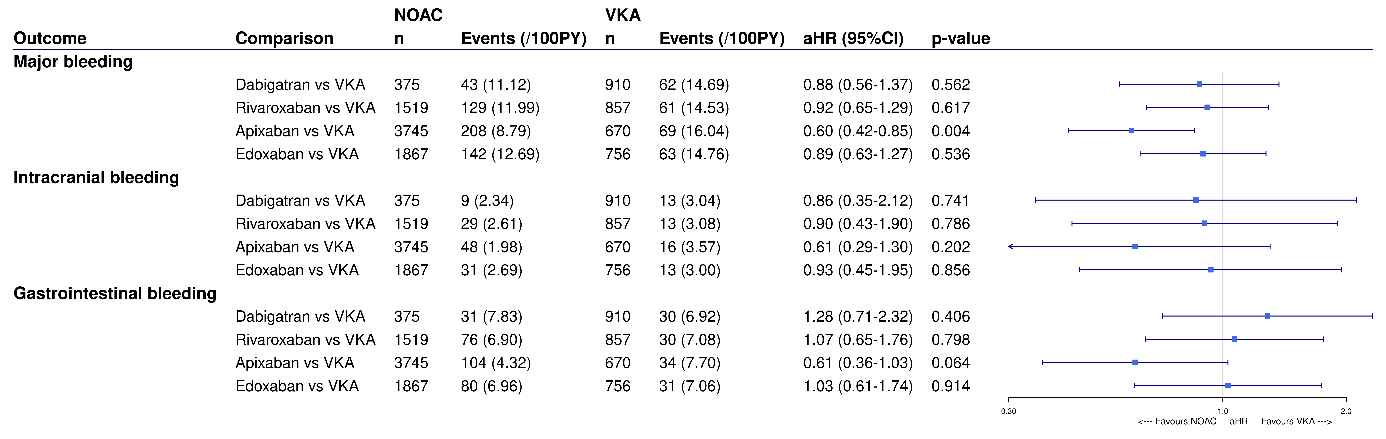
**

**B)**


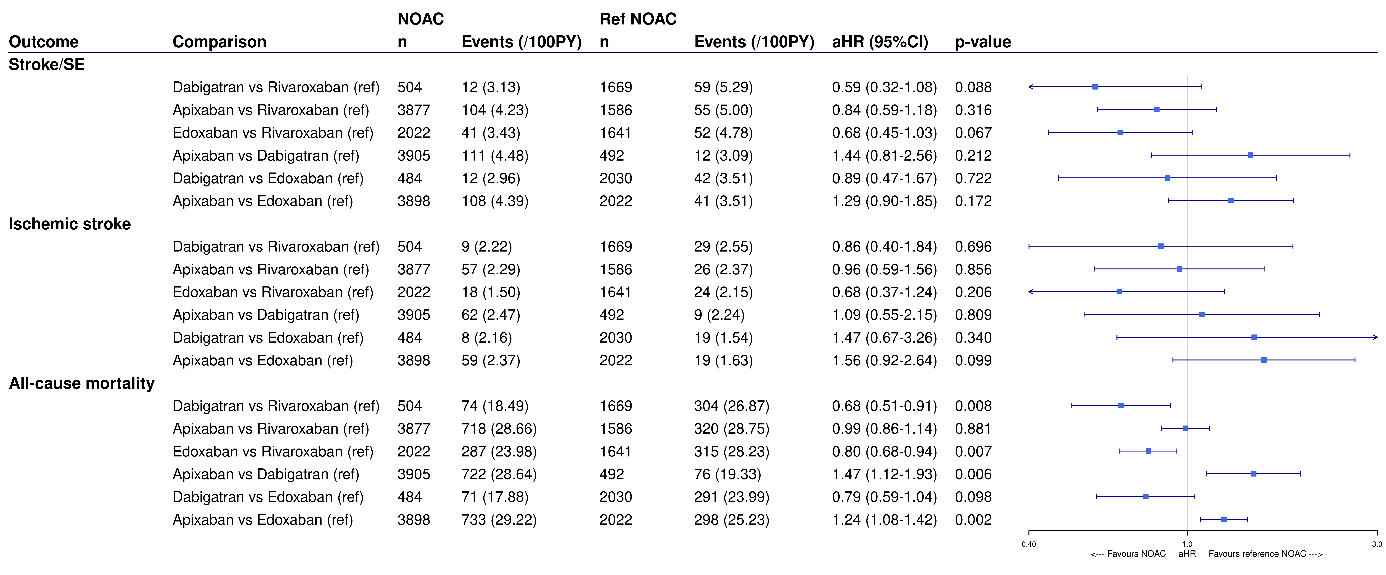


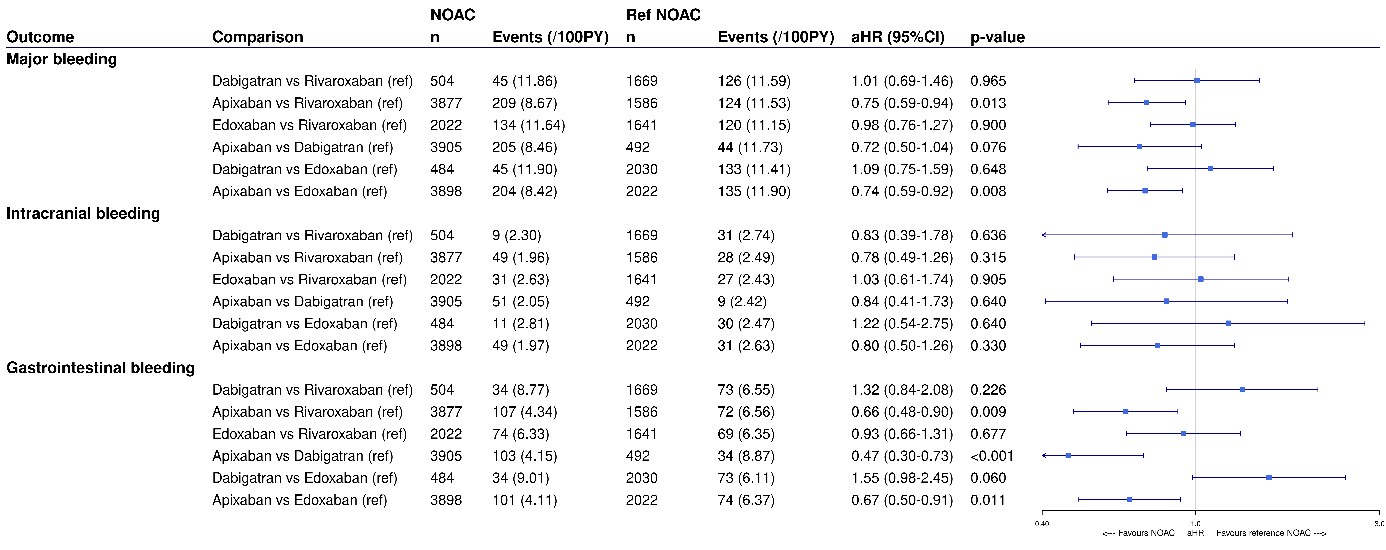


**eFigure 6:** The effectiveness and safety of **A)** NOACs versus VKAs, and **B)** between individual NOAC types in AF patients with a history of falls after IPTW in subgroup of subjects having initiated treatment between October 1^st^, 2016 and January 1^st^, 2019. The weighted number of subjects at risk in the pseudopopulation, weighted number of events, weighted event rates per 100 PY and adjusted HRs with 95%CIs after IPTW are illustrated. aHR: adjusted hazard ratio; CI: confidence interval; IPTW: inverse probability of treatment weighting; NOAC: non-vitamin K antagonist oral anticoagulant; PY: person-years; Ref: reference category; SE: systemic embolism; VKA: vitamin K antagonist; vs: versus.

# References

1. von Elm E, Altman DG, Egger M, Pocock SJ, Gøtzsche PC, Vandenbroucke JP. The Strengthening the Reporting of Observational Studies in Epidemiology (STROBE) statement: guidelines for reporting observational studies. Lancet (London, England). 2007;370(9596):1453-7.

2. The International Classification of Diseases (ICD), Clinical Modification. Available from: <https://www.cdc.gov/nchs/icd/index.htm>. Accessed 25 November 2021.

3. RIZIV/INAMI (Rijksinstituut voor ziekte- en invaliditeitsverzekering/Institut national d'assurance maladie-invalidité) medical procedure codes for claims of ambulatory and hospital care. Available from: <https://www.riziv.fgov.be/nl/nomenclatuur/Paginas/default.aspx> (in Dutch/French). Accessed 25 November 2021.

4. WHO Collaborating Centre for Drug Statistics Methodology. Available from: <https://www.whocc.no/>. Accessed 25 November 2021.

5. Segal JB, Chang HY, Du Y, Walston JD, Carlson MC, Varadhan R. Development of a Claims-based Frailty Indicator Anchored to a Well-established Frailty Phenotype. Medical care. 2017;55(7):716-22.

6. Hindricks G, Potpara T, Dagres N, Arbelo E, Bax JJ, Blomström-Lundqvist C, et al. 2020 ESC Guidelines for the diagnosis and management of atrial fibrillation developed in collaboration with the European Association of Cardio-Thoracic Surgery (EACTS). Eur Heart J. 2021;42(5):373-498.

7. Quan H, Li B, Couris CM, Fushimi K, Graham P, Hider P, et al. Updating and validating the Charlson comorbidity index and score for risk adjustment in hospital discharge abstracts using data from 6 countries. American journal of epidemiology. 2011;173(6):676-82.

8. Charlson ME, Pompei P, Ales KL, MacKenzie CR. A new method of classifying prognostic comorbidity in longitudinal studies: development and validation. Journal of chronic diseases. 1987;40(5):373-83.

9. Halvorsen S, Ghanima W, Fride Tvete I, Hoxmark C, Falck P, Solli O, et al. A nationwide registry study to compare bleeding rates in patients with atrial fibrillation being prescribed oral anticoagulants. European heart journal Cardiovascular pharmacotherapy. 2017;3(1):28-36.

10. Rutherford OW, Jonasson C, Ghanima W, Söderdahl F, Halvorsen S. Comparison of dabigatran, rivaroxaban, and apixaban for effectiveness and safety in atrial fibrillation: a nationwide cohort study. European heart journal Cardiovascular pharmacotherapy. 2020;6(2):75-85.
